# l-Glutamine in combination with first-line gemcitabine and nab-paclitaxel in advanced pancreatic ductal adenocarcinoma: an open-label, single-arm, phase 1 GlutaPanc trial

**DOI:** 10.1038/s43018-026-01225-z

**Published:** 2026-08-31

**Authors:** Jun Gong, Hayato Muranaka, So Yung Choi, Mourad Tighiouart, Shrikant Bhute, Ezinne R. Aja, Jonathan P. Jacobs, Aleksandr Stotland, Jennifer Van Eyk, Omer H. M. Elmadbouh, Mouad Edderkaoui, Sunao Tanaka, Hideki Furuya, Arsen Osipov, Jeremy Lorber, Sandrine Billet, Alexzandra Morris, Johanna ten Hoeve-Scott, Thomas Graeber, Stephen J. Pandol, Andrew Hendifar, Neil A. Bhowmick

**Affiliations:** 1 Department of Medicine, Division of Medical Oncology, Cedars-Sinai Medical Center, Los Angeles, CA USA; 2 Biostatistics Shared Resource, Cedars-Sinai Cancer Institute, Cedars-Sinai Medical Center, Los Angeles, CA USA; 3 The Vatche and Tamar Manoukian Division of Digestive Diseases, Department of Medicine, David Geffen School of Medicine, University of California, Los Angeles, CA USA; 4 Advanced Clinical Biosystems Research Institute, Cedars-Sinai Medical Center, Los Angeles, CA USA; 5 Department of Biomedical Science, Cedars-Sinai Medical Center, Los Angeles, CA USA; 6 Department of Molecular and Medical Pharmacology, Metabolomics Center, University of California, Los Angeles, CA USA; 7 Department of Research, VA Greater Los Angeles Healthcare System, Los Angeles, CA USA

**Keywords:** Cancer therapy, Pancreatic cancer, Cancer, Adaptive clinical trial

## Abstract

Exogenous l-glutamine has preclinical antitumor activity although formal clinical translation has not been attempted. We conducted a single-arm phase 1 trial to assess the safety and preliminary efficacy of clinical-grade, US Food and Drug Administration-approved l-glutamine therapy with gemcitabine and nab-paclitaxel (GA) in participants with treatment-naive, advanced pancreatic cancer (*n* = 16). The primary endpoint was to determine the recommended phase 2 dose (RP2D) by adaptive Bayesian design across standard doses of GA and a dose range of 0.1–0.3 g kg^−1^ twice-daily oral l-glutamine. Secondary endpoints included safety and preliminary efficacy of the study combination. The primary endpoint was met with the RP2D reached at maximum doses of l-glutamine and GA. The grade ≥3 treatment-related adverse event rate was 66.7%, primarily from GA. Addition of l-glutamine to GA induced tumor shrinkage in 94% of subjects with a best overall response rate (ORR) of 44% (12.5% complete response). Median progression-free survival and overall survival (OS) were 8.5 months (95% confidence interval (CI) 6–not reached (NR)) and 22 months (95% CI 11–NR), respectively. l-Glutamine induced distinct metagenomic and metabolomic signatures on exploratory analyses in glutamine-treated subjects as a single agent, while the combination of l-glutamine and GA nearly doubled the ORR and tripled the OS compared to historical GA alone (ClinicalTrials.gov registration: NCT04634539).

## Abstract (web summary)

In this open-label, single-arm, phase 1 GlutaPanc trial, Gong and colleagues report the safety and feasibility of l-glutamine in participants with advanced pancreatic ductal adenocarcinoma and determine the recommended phase 2 dose of l-glutamine in combination with gemcitabine and nab-paclitaxel.

## Main

Pancreatic ductal adenocarcinoma (PDAC) has a dismal 5-year survival rate of 3% with stage IV or metastatic disease^1^. A foundation of metastatic PDAC treatment is cytotoxic chemotherapy combinations inclusive of gemcitabine; however, second and third-line chemotherapy administration rates approximate 45% and 21%, respectively^2^. These findings reinforce the importance of optimizing first-line chemotherapy given the aggressive nature of advanced PDAC that is often underscored by progressive therapeutic resistance.

l-Glutamine is the most abundant amino acid in humans and, although considered nonessential, it can be essential for growth and survival for PDAC tumors^3^. The disruption of l-glutamine uptake and metabolism is reported to sensitize PDAC cells to chemotherapy^4^. Unfortunately, therapeutic effects are short lived as scavenger pathways seem to compensate and result in treatment failure^5^. On the other hand, l-glutamine supplementation is shown to support improved gut health and even therapeutic benefit in preclinical cancer models for which the mechanism is yet to be understood^6–11^. Adjunctive therapy of l-glutamine has been used to mitigate toxicities in persons with cancer receiving chemotherapy and radiation therapy^12^. However, none of these prior trials used a clinical-grade formulation of l-glutamine, approved by the US Food and Drug Administration (FDA) for persons with sickle cell anemia^13^.

To this end, we conducted a clinical trial investigating the safety and feasibility of l-glutamine (Endari, Emmaus Medical) in human subjects with predominantly metastatic PDAC. The primary endpoint of this dose-finding phase 1 trial was to determine the recommended phase 2 dose (RP2D) using escalating doses of l-glutamine in combination with standard gemcitabine and nab-paclitaxel (GA). Secondary objectives included assessing early signals of efficacy and impact on the gut microbiome composition, inflammatory response and metabolomic profiles.

## Results

### Participant characteristics

**Fig. 1:**
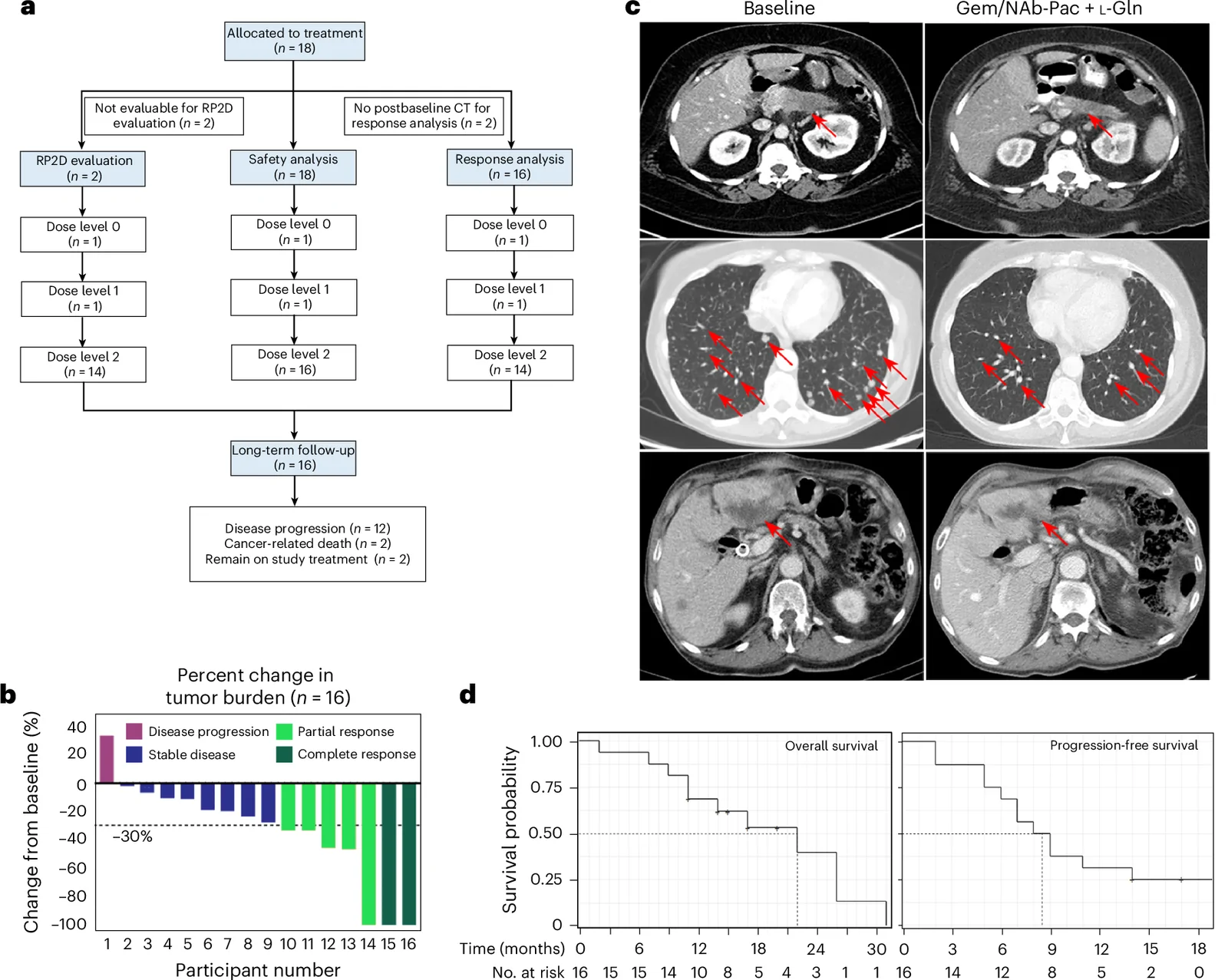
CONSORT study flowchart of the phase 1 GlutaPanc trial and efficacy outcomes of oral l-glutamine in combination with GA. **a**, Dosing levels for this phase 1 trial were as follows: dose level −2 consisting of gemcitabine 600 mg m^−2^ on days 1, 8 and 15, nab-paclitaxel 75 mg m^−2^ on days 1, 8 and 15 and l-glutamine 0.1 g kg^−1^ oral twice daily; dose level −1 consisting of gemcitabine 800 mg m^−2^ on days 1, 8 and 15, nab-paclitaxel 100 mg m^−2^ on days 1, 8 and 15 and l-glutamine 0.1 g kg^−1^ oral twice daily; dose level 0 consisting of gemcitabine 1,000 mg m^−2^ on days 1, 8 and 15, nab-paclitaxel 125 mg m^−2^ on days 1, 8 and 15 and l-glutamine 0.1 g kg^−1^ oral twice daily; dose level 1 consisting of gemcitabine 1,000 mg m^−2^ on days 1, 8 and 15, nab-paclitaxel 125 mg m^−2^ on days 1, 8 and 15 and l-glutamine 0.2 g kg^−1^ oral twice daily; dose level 2 consisting of gemcitabine 1,000 mg m^−2^ on days 1, 8 and 15, nab-paclitaxel 125 mg m^−2^ on days 1, 8 and 15 and l-glutamine 0.3 g kg^−1^ oral twice daily. The starting dose level was 0 and there was an allocation of *n* = 1 subject each into dose levels 0 and 1, followed by escalation to dose level 2 for the remaining *n* = 14 subjects. There were no deescalations to dose levels −1 and −2. The *n* = 16 subjects in long-term follow-up comprised the same 16 subjects in the evaluable for RP2D and response analysis cohorts. **b**, Waterfall plot indicating best change in tumor target lesions from baseline and best ORR by category. Tumor shrinkage was observed in 15/16 (94%) of subjects with an ORR of 44% (7/16 subjects). There were two CRs (12.5%) and five PRs (31.3%), along with eight cases of SD (50%) and one case of PD (6.2%), as the best overall response. **c**, Computed tomography imaging of subjects with indicated primary and metastatic PDAC lesions before and after l-glutamine plus GA treatment. Tumor shrinkage was defined as any reduction in tumor target lesions from baseline (arrows). **d**, Kaplan–Meier curves of OS and PFS of subjects treated with l-glutamine combined with GA (*n* = 16). Source data

A final cohort of 16 advanced PDAC subjects (15 metastatic and one locally advanced or unresectable) were enrolled from May 13, 2021 to June 2, 2023, with the most common metastatic sites being the liver, lung, peritoneum and lymph nodes (Fig. 1 and Table 1). Most subjects met consensus criteria for cachexia (81%) at baseline, with 25% having undergone prior surgery for localized PDAC.

**Table 1:** Safety, treatment characteristics and participant disposition of the phase 1 GlutaPanc clinical trial

Characteristic (*n* = 16)	*n* (%)
Age, median (range)	71 (55–92)
Gender	
Female	11 (69%)
Male	5 (31%)
ECOG performance status	
0	4 (25%)
1	11 (69%)
2	1 (6%)
Cachexia at study entry^a^	
Yes	13 (81%)
No	3 (19%)
Weight (kg) at study entry, mean (s.d.)	73.1 (21.3)
BMI (kg m^−2^) at study entry, mean (s.d.)	26.2 (6.4)
Prior surgery	
Distal	1 (6%)
Whipple	3 (19%)
None	12 (75%)
Sites of metastases^b^	
Liver	8/19 (42%)
Lung	5/19 (26%)
Peritoneum	3/19 (16%)
Lymph nodes (nonregional)	3/19 (16%)
Median number of cycles (range)	8 (2–15)
Treatment discontinuation	14
Disease progression	12
Death	2
DLTs	3
Grade 3 febrile neutropenia	1
Grade 4 thrombocytopenia	2
Dose level allocation^c^	
0	1
1	1
2	14

^a^Defined as >5% unexplained weight loss within 6 months before screening.

^b^Subjects may have had more than one site of metastasis at study entry.

^c^At every 28-day cycles, dose level 0 corresponded to 1,000 mg m^−2^ gemcitabine intravenous (IV) D1, 8, 15/125 mg m^−2^ nab-paclitaxel IV D1, 8, 15/ʟ-glutamine 0.1 g kg^−1^ oral twice daily; dose level 1 corresponded to 1,000 mg m^−2^ gemcitabine IV D1, 8, 15/125 mg m^−2^ nab-paclitaxel IV D1, 8, 15/ʟ-glutamine 0.2 g kg^−1^ oral twice daily; dose level 2 corresponded to 1,000 mg m^−2^ gemcitabine IV D1, 8, 15/125 mg m^−2^ nab-paclitaxel IV D1, 8, 15/ʟ-glutamine 0.3 g kg^−1^ oral twice daily.

ECOG, Eastern Cooperative Oncology Group; BMI, body mass index.

All enrolled subjects with treatment-naive, advanced PDAC receiving one dose of GA were evaluable for safety (NCI CTCAE version 5.0). DLTs were evaluated during the first 28-day cycle of GA in combination with oral l-glutamine.

### Dose finding and safety

**Extended Data Fig. 1:**
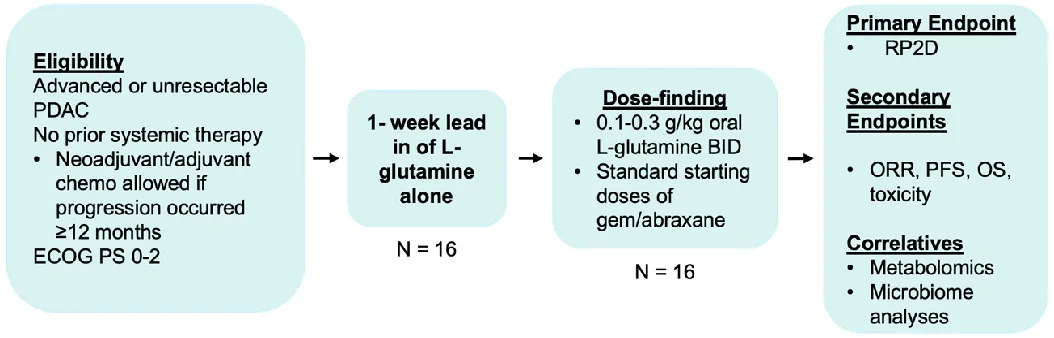
Study schema. Fasted blood samples by peripheral venous draw were collected at pretreatment (baseline), after the 1-week lead-in phase of oral L-glutamine alone (C1D1), and after the first dose of gemcitabine and nab-paclitaxel while on oral L-glutamine (C1D8). Stool samples were collected at baseline and after oral L-glutamine alone (C1D1). PDAC, pancreatic ductal adenocarcinoma; ECOG PS, Eastern Cooperative Oncology Group performance status; BID, twice daily; RP2D, recommended phase II dose; ORR, overall response rate; PFS, progression-free survival; OS, overall survival.

This was a single-arm, dose-finding phase 1 trial where all 16 subjects completed a 1-week oral l-glutamine lead-in before receiving a median of eight cycles (range 2–15) of GA and l-glutamine for the evaluation of RP2D (Extended Data Fig. 1 and Table 1). At the time of data cutoff, the median follow-up was 20 months (95% confidence interval (CI) 17–not reached (NR)) and two subjects remained on treatment. Of the 14 subjects who discontinued the study, 12 were because of disease progression and two were because of cancer-related death (Fig. 1a). There were no treatment-related study discontinuations. An initial 18 subjects were evaluable for safety but two were deemed unevaluable for the dose-finding primary endpoint. Dose finding to determine the RP2D involved five dose levels of l-glutamine in combination with GA following the extended escalation with overdose control (EWOC) algorithm based on dose-limiting toxicities (DLTs) in the first 28-day cycle^14^. The starting doses (dose level 0) for GA were the standard maximum of gemcitabine 1,000 mg m^−2^ and nab-paclitaxel 125 mg m^−2^ on days 1, 8 and 15, with deescalation permitted to 800 mg m^−2^ (dose level −1) or 600 mg m^−2^ (dose level −2) for gemcitabine and 100 mg m^−2^ (dose level −1) or 75 mg m^−2^ (dose level −2) for nab-paclitaxel on the basis of toxicity (Supplementary Table 1). Starting doses for oral l-glutamine were 0.1 g kg^−1^ twice daily with escalation to maximum 0.3 g kg^−1^ twice daily, as tolerated. Of note, escalation beyond standard doses for GA and deescalation below starting doses for l-glutamine were not allowed. Beginning at dose level 0, the majority (*n* = 14) of subjects were escalated to the highest dose level 2. During the DLT assessment window, a total of three DLTs were encountered all deemed treatment-related to GA and not oral l-glutamine. The trial was closed once 16 participants were evaluable for DLT and the RP2D was estimated as the median of the posterior distribution of the maximum tolerated dose (MTD), which was found to be dose level 2. This corresponded to maximum doses of oral l-glutamine (30 g per day orally) with full standard doses of gemcitabine (1,000 mg m^−2^) and nab-paclitaxel (125 mg m^−2^). l-Glutamine was generally well tolerated with most treatment-related adverse events (AEs) consistent with the chemotherapy backbone (Supplementary Tables 2 and 3).

### Efficacy outcomes

Of 16 evaluable subjects, there were two complete responses (CRs) and five partial responses (PRs), corresponding to a best overall response rate (ORR) of 44% and a CR rate of 12.5% (Fig. 1b). Tumor reduction was observed in 94% of subjects (15 of 16), with a mean reduction in tumor target lesions from baseline of −34.1% ± 37.7% (Fig. 1b,c). As of May 2024, five subjects remained alive. Using the reverse Kaplan–Meier method, the median follow-up at the time of data cutoff was 20 months (95% CI 17–NR). There were eight subjects experiencing stable disease (SD) and one subject with progressive disease (PD) as the best response. The median overall survival (OS) was 22 months (95% CI 11–NR) with 12-month and 24-month OS rates of 69% (95% CI 49–96%) and 40% (95% CI 19–84%), respectively (Fig. 1d). The median progression-free survival (PFS) was 8.5 months (95% CI 6–NR) with 6-month and 12-month PFS rates of 69% (95% CI 49–96%) and 25% (95% CI 11–58%), respectively (median follow-up: 17 months, 95% CI 14–NR). Postprogression therapies such as FOLFIRINOX and 5-fluorouracil/liposomal irinotecan were among the predominant second-line regimens providing a median PFS of 2 months (Supplementary Table 4).

### Glutamine effects on intermediary metabolism

**Fig. 2:**
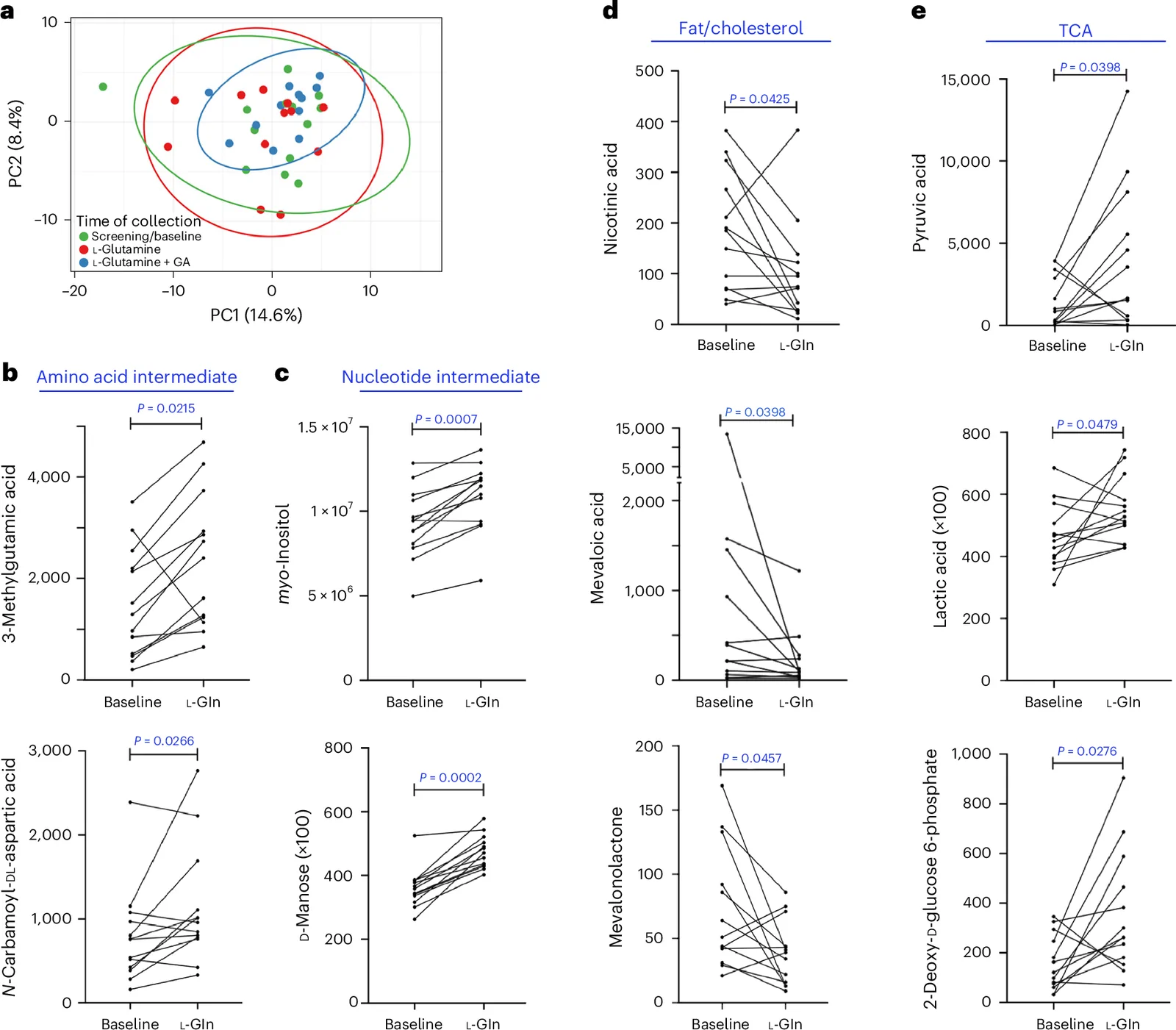
Pharmacodynamic effects of l-glutamine treatment on circulating glutamine intermediary metabolism. Blood samples collected at fasting (same time point at baseline) after the l-glutamine lead-in period with GA were subjected to plasma metabolomics. **a**, Principal component analysis of the 219 polar metabolites for the three time points illustrated. **b**–**e**, The paired relative plasma levels of the metabolites 3-methylgutamic acid and *N*-carbamoyl-ᴅʟ-aspartic acid (**b**); *myo*-inositol and ᴅ-manose (**c**); nicotinic acid, mevaloic acid and mevalonolactone (**d**); and pyruvic acid, lactic acid and 2-deoxy-ᴅ-glucose 6-phosphate (**e**) in the respective pathways altered by l-glutamine treatment compared to baseline were subjected to a Wilcoxon two-sided test with *P* < 0.05 being statistically significant. Each individual line plot represents *n* = 1 subject for each metabolite comparison or *n* = 13 for **b**–**e** comparisons. Source data

**Fig. 3:**
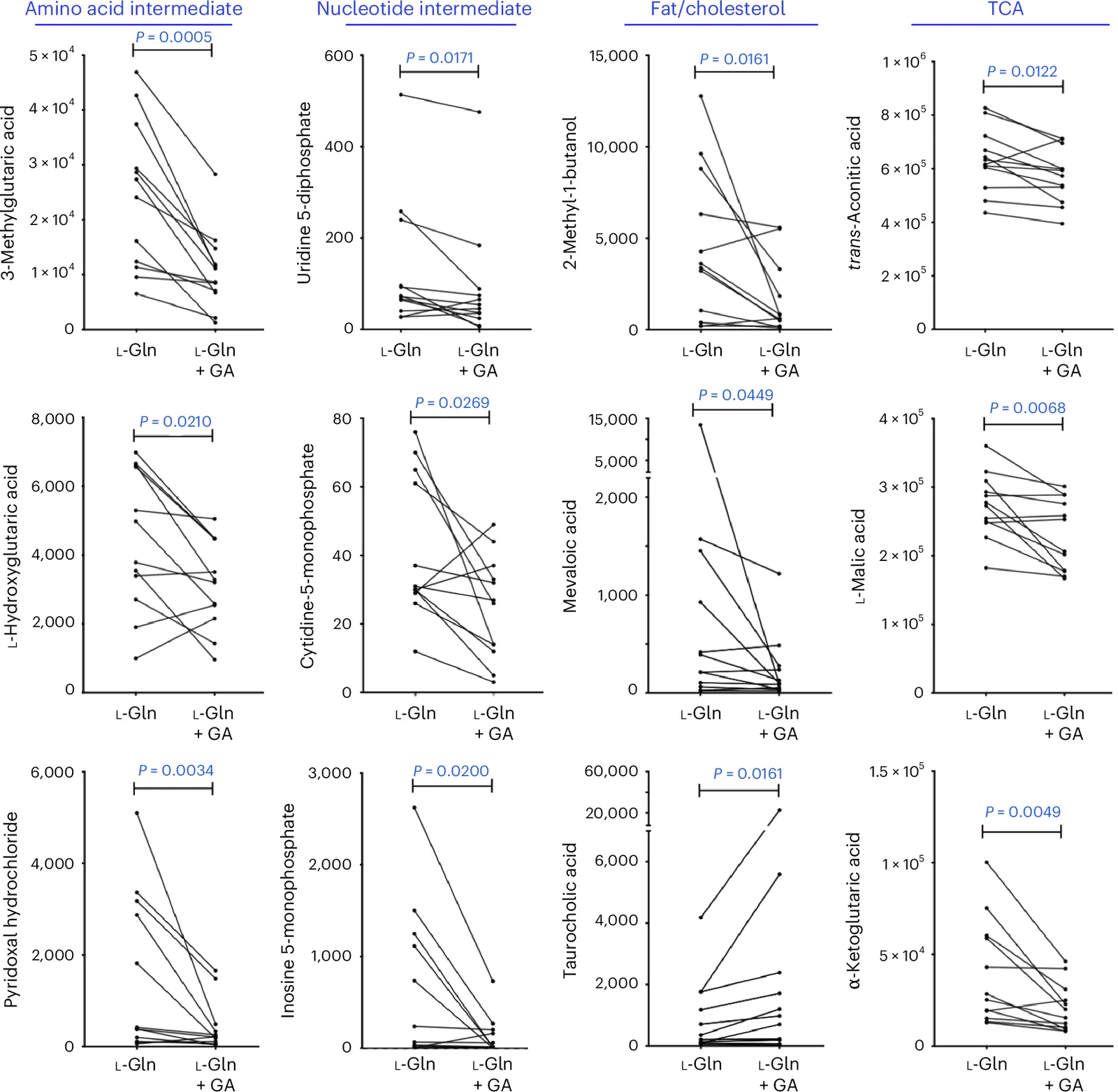
Metabolite changes following the introduction of the first dose of GA. Blood samples collected at fasting (same time point following l-glutamine treatment) and after the first dose of GA while on oral l-glutamine were similarly subjected to plasma metabolomics using a dedicated triple-quadrupole LC–MS system where up to 219 polar metabolites of central carbon metabolism. The paired relative plasma levels of metabolites altered by l-glutamine in the presence or absence of chemotherapy treatment were subjected to a two-sided Wilcoxon test with *P* < 0.05 being statistically significant. Each individual line plot represents *n* = 1 subject for each metabolite comparison or *n* = 12 for all comparisons. Source data

**Fig. 4:**
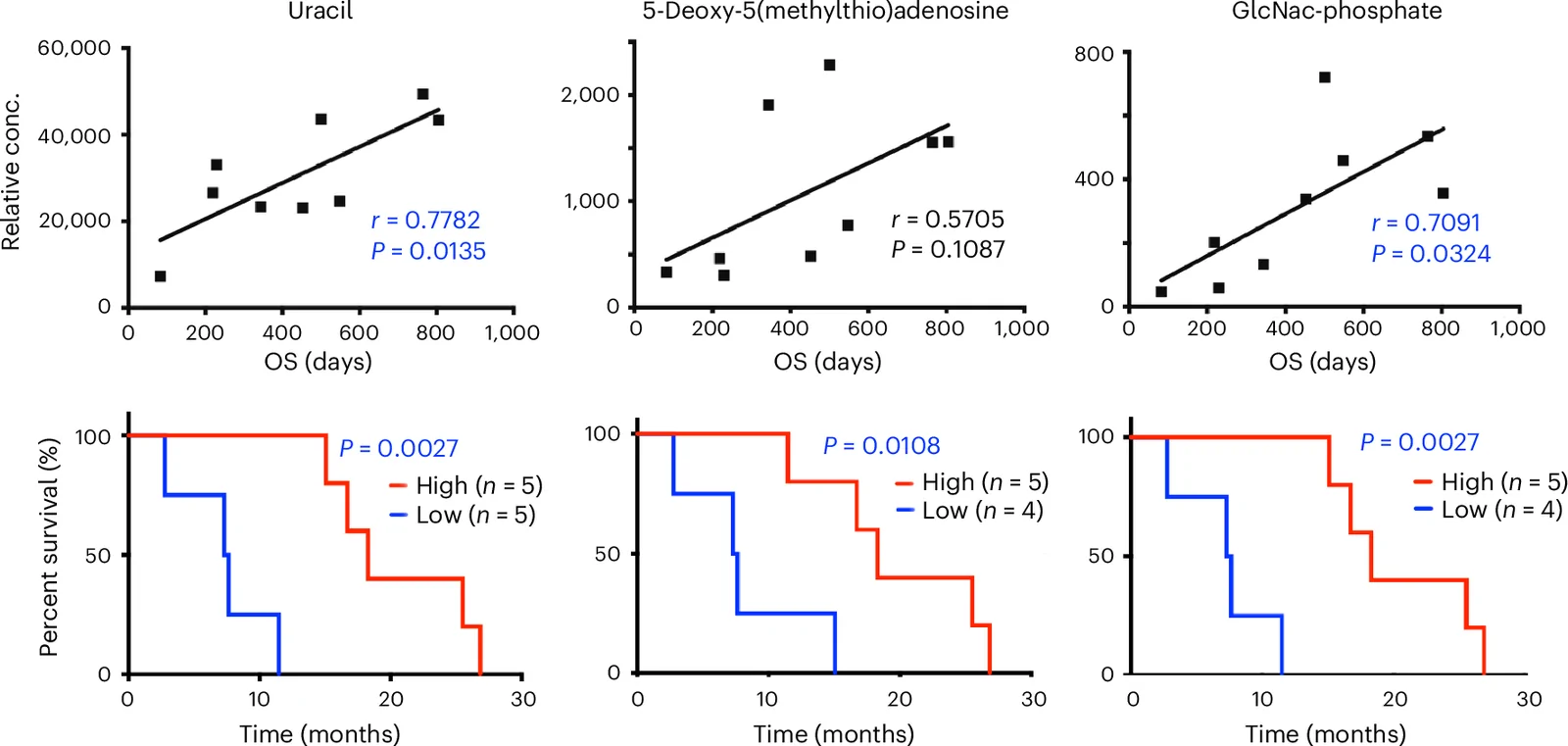
Plasma metabolites associated with OS and time to survival in participants with metastatic PDAC at baseline. Top: the indicated plasma nucleotide biosynthesis metabolites of participants were correlated at baseline with OS. Each dot in scatter plot represents *n* = 1 subject for each metabolite. Bottom: Kaplan–Meier OS curves were generated for indicated plasma metabolites, where high (red) and low (blue) levels were plotted by the log-rank (Mantel–Cox) test in participants treated with oral l-glutamine and GA. Statistical significance at *P* < 0.05 is indicated in blue text. Source data

**Extended Data Fig. 2:**
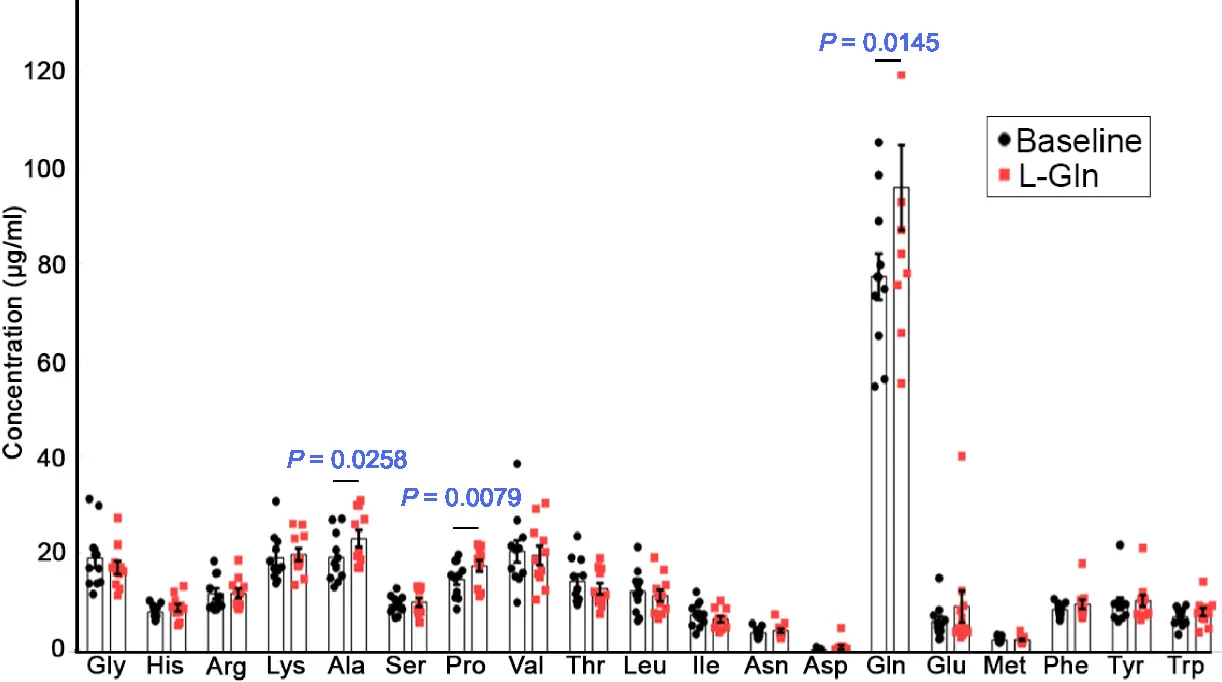
Plasma amino acid concentrations in response to L-glutamine lead-in period. Fasted blood samples collected at the same time point before at baseline (gray) and after the oral L-glutamine lead-in period (red) were subjected to plasma metabolomics and represent pre- and post-L-glutamine samples from n = 11 subjects. The paired relative plasma levels of metabolites altered by L-glutamine treatment (red data points) were compared to baseline (black data points) by two-sided Student’s t-test. Standard deviation indicated by error bars. P-values provided when statistically significant with a *P* < 0.05. Source Data

To better understand the clinical observations, preplanned correlatives of blood and stool were collected at baseline and after the l-glutamine lead-in (C1D1), while an additional blood sample was collected after the first dose of GA (C1D8; Supplementary Table 5). Plasma glutamine concentrations were significantly elevated with l-glutamine treatment, when compared to baseline, confirming the on-target effects of l-glutamine supplementation in the absence of GA chemotherapy (Extended Data Fig. 2). l-Alanine and l-proline levels were also significantly increased from baseline following l-glutamine treatment, suggestive of downstream pharmacodynamic effects on intermediary metabolism, given that glutamine serves as a precursor for both alanine and proline synthesis. Anaplerosis and nucleotide biosynthesis were expectedly elevated in the pentose phosphate pathway and UDP-GlcNAc pathway according to plasma metabolomics, compared to baseline levels (Fig. 2). However, fatty acid and cholesterol metabolites were depleted under the same conditions. The subsequent addition of GA to l-glutamine treatment caused a general reduction in amino acid intermediates, nucleotides and TCA cycle, fatty acid and cholesterol metabolites (Fig. 3). At the time of screening, elevated levels of circulatory nucleotide biosynthesis metabolites had a positive correlation with OS, being among determinants of longer survival than the median historical OS of GA (Fig. 4)^15^. The exploratory and outcome-informed nature of selecting and stratifying nucleotide biosynthesis metabolites warrants further evaluation of these hypothesis-generating findings in a larger study for validation as potential biomarkers. In short, on-target effects on glutamine concentrations with subsequent uptake into intermediary pathways were observed following glutamine treatment and independent of GA chemotherapy.

### Glutamine effects on bacterial metabolism

**Fig. 5:**
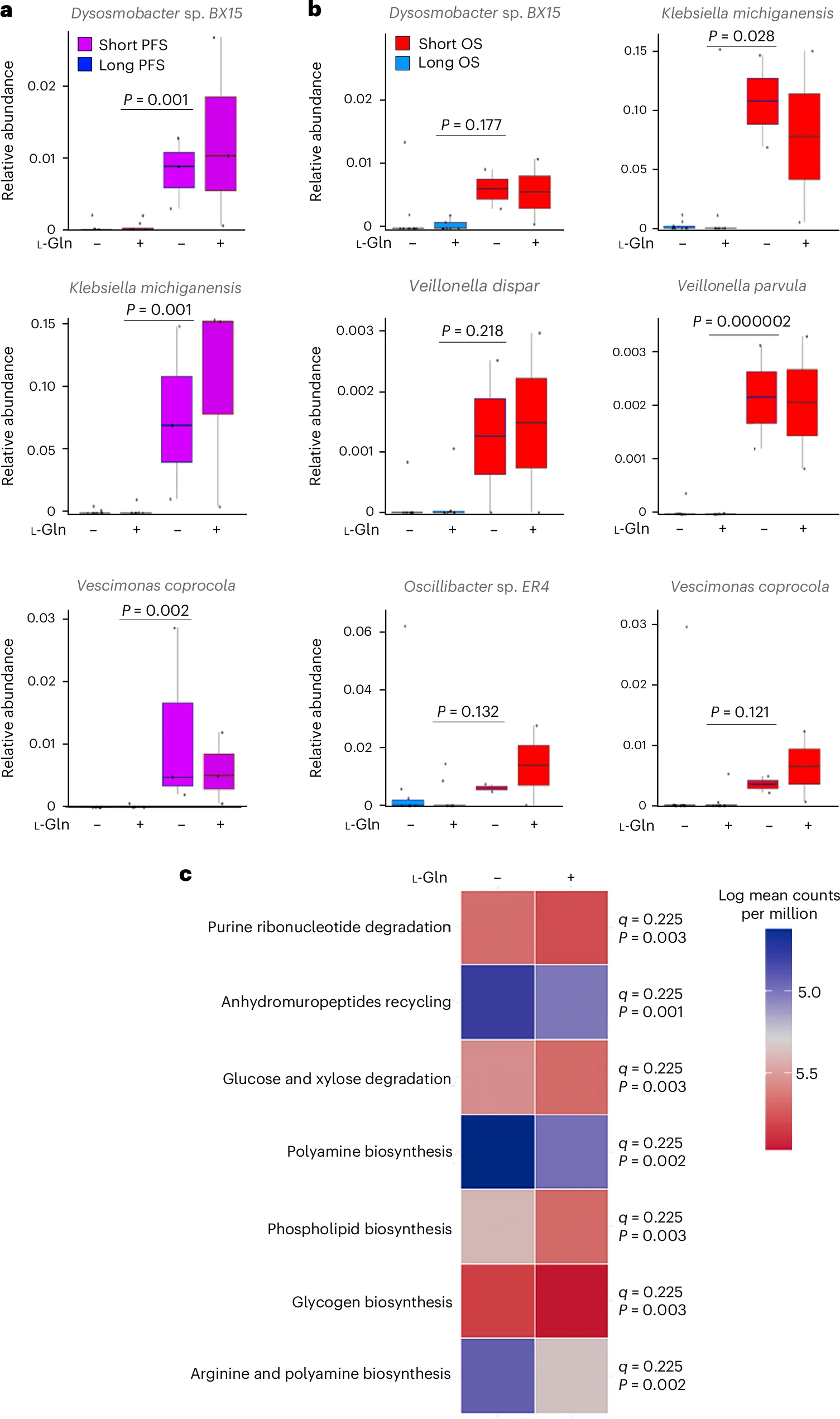
Differentially abundant taxa associated with OS in participants with metastatic PDAC before and after oral l-glutamine treatment (*n* = 12). Differential abundance of bacterial species and metabolic pathways was determined using stool samples collected at baseline and after completion of the l-glutamine treatment. **a**, Bacterial species that significantly differed between those who achieved a prolonged versus those who had a short PFS were delineated, where long PFS was defined by those participants having surpassed the historical median PFS (165 days) of first-line GA. Differential abundance was evaluated using linear mixed-effects models implemented in MaAsLin2, with short versus long OS and time point as fixed effects and subject as a random effect. The *P* values reported were calculated directly by MaAsLin2 using its internal multivariable linear modeling framework. Box plots indicate the center line (median), the bounds of the box (25th and 75th percentiles) and whiskers extending to the minima and maxima within 1.5× the interquartile range (*n* = 11 baseline (−) and *n* = 12 l-glutamine (+) for PFS comparisons). **b**, Bacterial species that significantly differed between those who achieved a prolonged OS versus those who had a short OS, where long OS was defined by those participants having surpassed the historical median OS (258 days) of first-line GA. MaAsLin2 models included short versus long OS and time point as fixed effects and subject as a random effect. The *P* values reported were calculated directly by MaAsLin2 using its internal multivariable linear modeling framework. Box plots indicate the center line (median), the bounds of the box (25th and 75th percentiles) and whiskers extending to the minima and maxima within 1.5× the interquartile range (*n* = 12 baseline (−) and *n* = 11 l-glutamine (+) for OS comparisons). **c**, Heat map of the abundance of bacterial MetaCyc pathways that significantly differed in stool samples collected before initiation of the oral l-glutamine lead-in phase (baseline) compared to after completion of the l-glutamine lead-in period. Differential abundance of bacterial metabolic pathways was evaluated using two-sided linear mixed-effects models in MaAsLin2 with time point as a fixed effect and subject as a random effect. Multiple-comparison adjustments across features were performed natively within MaAsLin2 using the Benjamini–Hochberg FDR method. Associations with a *q* value ≤ 0.25, following FDR correction, were considered significant. Source data

**Extended Data Fig. 3:**
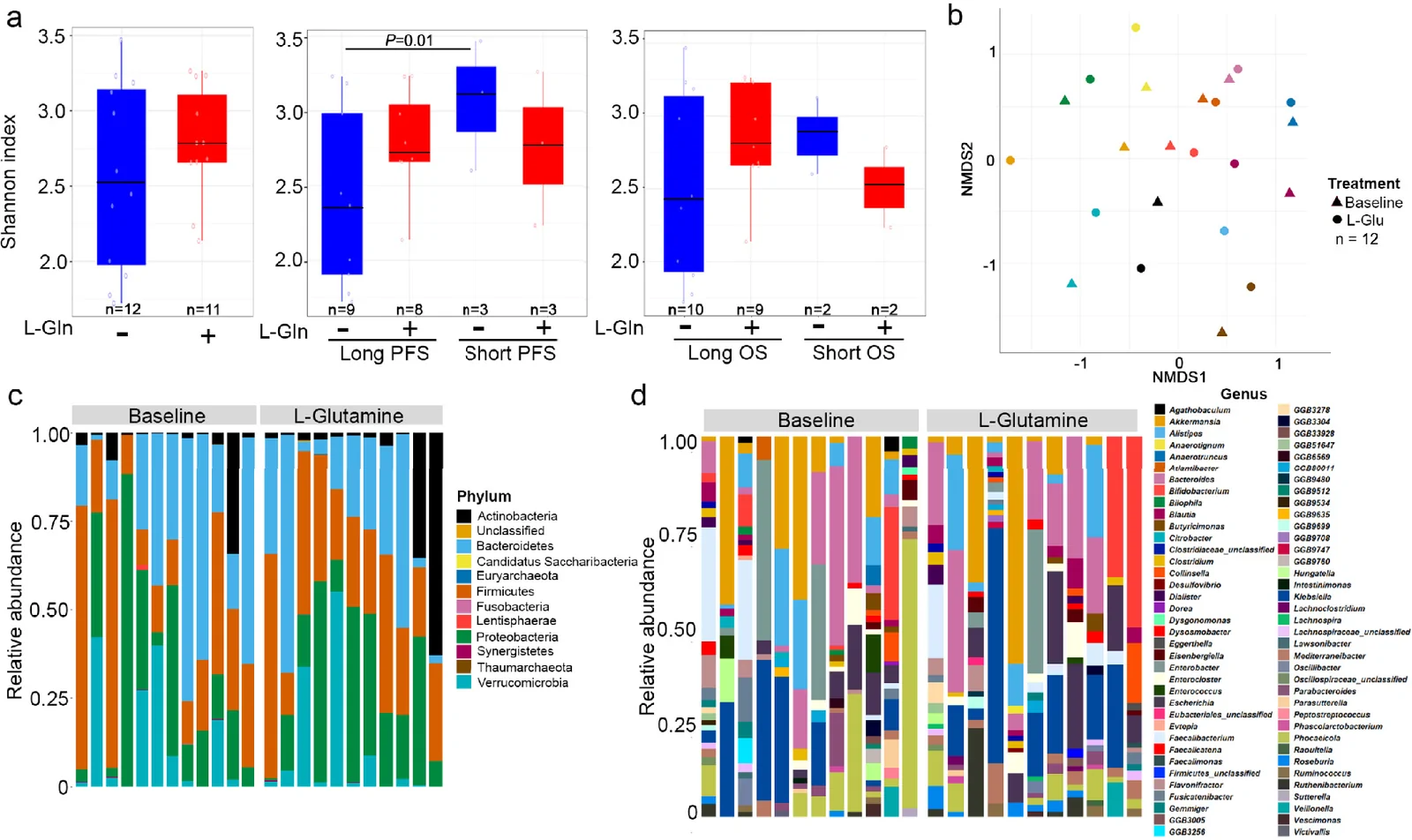
Diversity and abundance of gut microbiome before and after L-glutamine treatment in patients with metastatic PDAC (n = 12). **a**. Alpha diversity of the gut microbiome in patients in response to L-glutamine, with or without stratification by long vs. short PFS or OS was assessed by the Shannon index (reflecting microbial richness and evenness) from stools collected. Statistical significance was assessed using a two-sided linear mixed-effects model (LMM) to account for the repeated-measures design, with subject ID as a random effect. Treatment, long/short PFS and long/short OS statuses were included as fixed effects; p-values were derived using Satterthwaite’s approximation with no adjustments for multiple comparisons. Box plots indicate the center line (median), the bounds of the box (25th and 75th percentiles), and whiskers extending to the minima and maxima within 1.5× the interquartile range. Long OS was defined by patients whose median OS surpassed the historical median OS (258 days), while long PFS was defined by those patients having surpassed historical median PFS (165 days) of first-line gemcitabine and nab-paclitaxel alone. Please refer to Extended Data Table 6 for sample sizes across long PFS/OS vs. short PFS/OS comparisons made by two-sided Student’s t-test **b**. NMDS (non-metric multidimensional scaling) ordination plot of microbial community composition based on Bray-Curtis dissimilarity. Each point represents a sample, with triangles representing baseline, circles representing L-glutamine lead-in samples, and color indicating individual subjects. PERMANOVA with subject as strata did not demonstrate a significant difference between the two timepoints (R^2^ = 0.025, F = 0.49, p = 0.99). **c, d**. Relative abundance of gut microbes at the phylum and genus levels in patients with metastatic PDAC before and after oral L-glutamine treatment are illustrated. Each bar represents one subject, with each patient’s baseline and L-glutamine timepoints paired. All visualizations were created using R ggplot2 package. n = 12 for baseline and n = 11 following L-glutamine lead-in for b, c, and d comparisons. Source data

Intestinal microbiome composition and function was assessed by shotgun metagenomics of stool collected at baseline and after l-glutamine lead-in. No effect was seen of l-glutamine on alpha diversity as measured by the Shannon index (*P* = 0.24) and beta diversity by Bray–Curtis dissimilarity (*R*^2^ = 0.025, *P* = 0.99; Supplementary Table 6 and Extended Data Fig. 3a,b). However, a significant association was observed in participants with short PFS, exhibiting a higher basal alpha diversity compared to those with long PFS (*P* = 0.01). The consistent directional trend toward increased alpha diversity with l-glutamine treatment among subjects with long OS and PFS suggested a potential type II error because of limited sample size. There were discernable changes in abundance across gut microbes because of l-glutamine treatment (Extended Data Fig. 3c,d). Significant associations were found between the relative abundance of select gram-negative bacteria for both PFS and OS (Fig. 5a,b). Broadly, those participants experiencing longer survival on glutamine-based therapy had reduced abundance of gram-negative bacteria at baseline and after l-glutamine treatment.

**Fig. 6:**
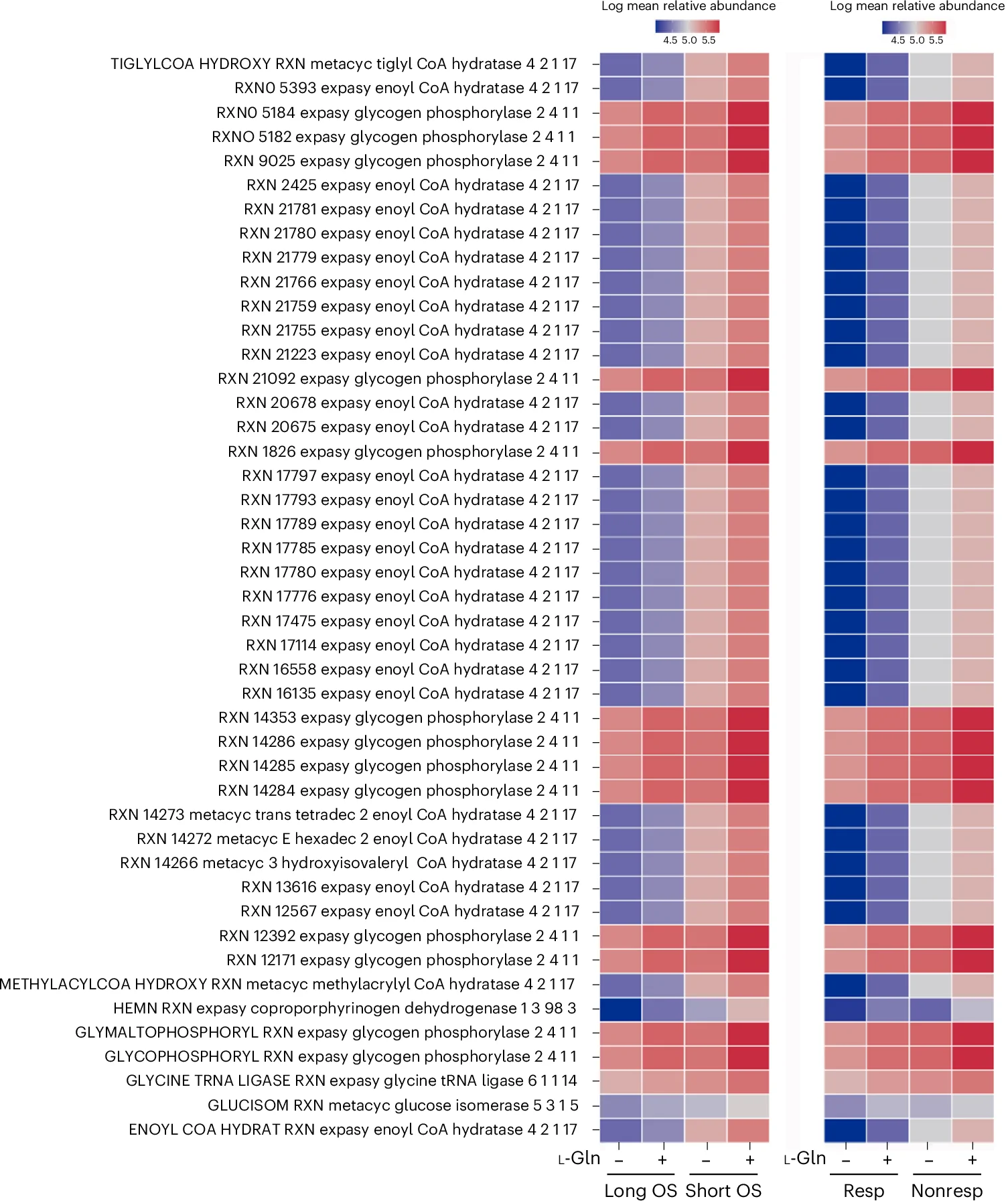
Bacterial gene families in stool from participants with metastatic PDAC receiving l-glutamine treatment that were differentially abundant according to survival and tumor response. Heat map of the abundance of bacterial genes detected in stool samples collected from participants achieving a tumor response (CR or PR) or not (SD or PD) and achieving a prolonged OS or not (short OS) to l-glutamine and GA. Stool samples were collected as indicted, before the oral l-glutamine lead-in week (baseline) and after the oral l-glutamine lead-in week. Long OS was defined by participants whose median OS surpassed the historical median OS (258 days) of first-line GA. The differential abundance of gene families was evaluated using linear mixed-effects models implemented in MaAsLin2 with short OS versus long OS (or responder versus nonresponder) and time point as fixed effects and subject as a random effect. Gene families are listed by reaction identifier and enzyme name; identical enzymes appearing multiple times represent distinct reactions or pathways. All associations between l-glutamine treatment and bacterial genes were statistically significant with *P* < 0.002 and *q* ≤ 0.25 following FDR correction. Source data

As glutamine did not impact the overall gut microbiome composition, the impact on bacterial metabolism was measured^16^. Understandably, polyamine, phospholipid and glycogen biosynthesis pathways were enriched by the gut bacteria with l-glutamine treatment, independent of GA chemotherapy (Fig. 5c). Elevation in glycogen phosphorylase activity by all subjects with l-glutamine treatment was observed, suggestive of glycolytic activity (Fig. 6). Among responsive participants, l-glutamine treatment reduced enoyl CoA hydratase activity in multiple reactions required for fatty acid oxidation by gram-negative bacteria. The metabolic shift in long OS individuals was the typical response to nutrient availability. Conversely, the generally higher gram-negative bacteria content in the gut of short OS participants under basal conditions may favor greater l-glutamine use for observed elevation in fatty acid oxidation over anaplerosis.

### Glutamine limits systemic inflammatory response and malabsorption while maintaining gut barrier integrity

**Fig. 7:**
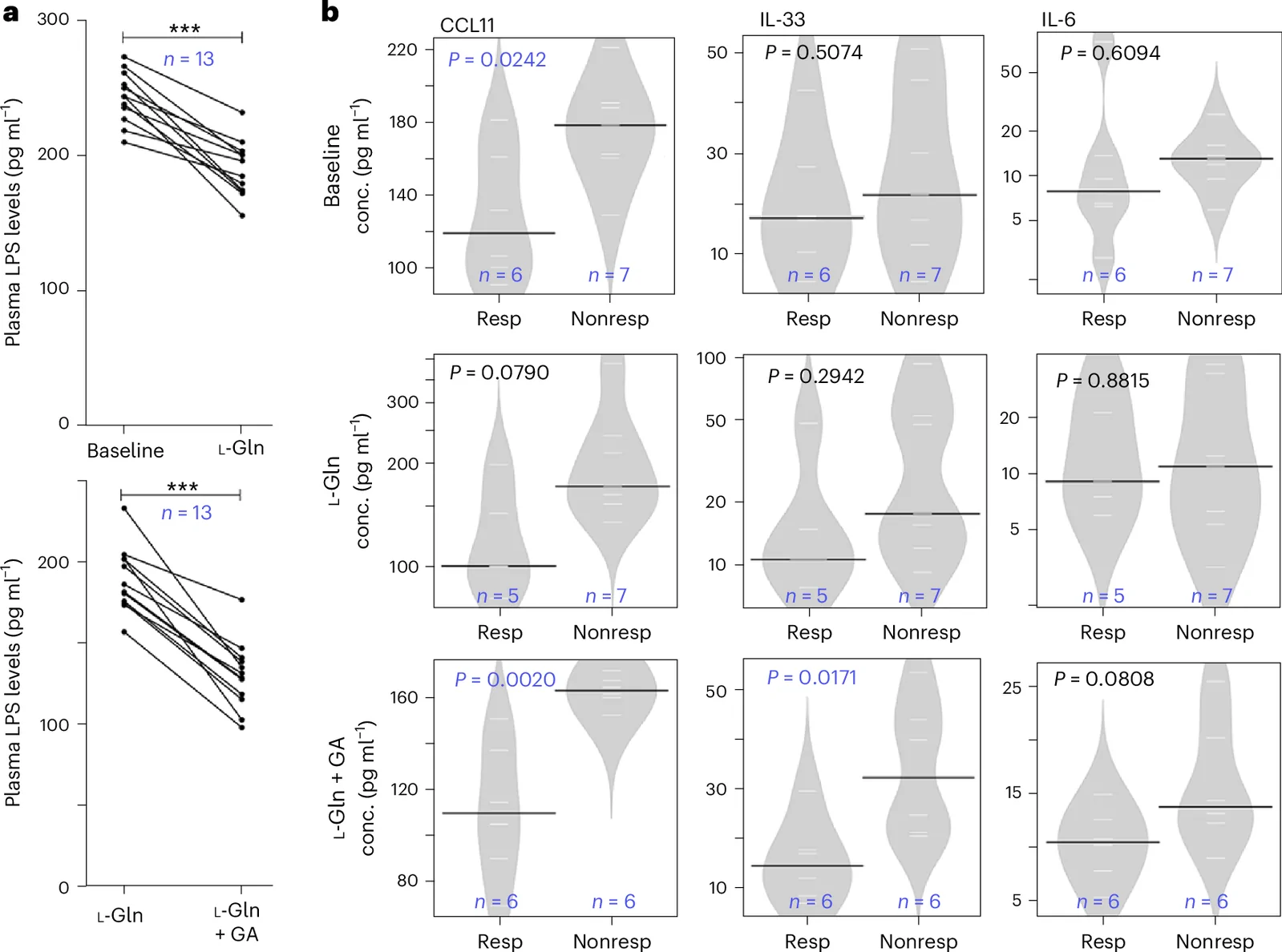
Change in inflammation and tumor response by treatment with l-glutamine and GA. **a**, Plasma LPS levels were measured at baseline, following 1 week of l-glutamine treatment and combination treatment of l-glutamine and GA. Each individual line plot represents *n* = 1 subject for each LPS comparison. ***P < 0.001. **b**, Differential expression of LPS-regulated cytokines shown in a violin plot, stratified by responders and nonresponders at the indicated time points of plasma collection: at baseline, following l-glutamine treatment and following the combination treatment of l-glutamine and GA. Responders were participants achieving CR or PR and nonresponders were those having SD or PD. Solid white lines represent each individual subject, and solid black lines represent the median. Statistical testing was conducted using a two-sided Wilcoxon test with statistical significance (*P* < 0.05) indicated in blue text for **a**,**b**. Source data

**Extended Data Fig. 4:**
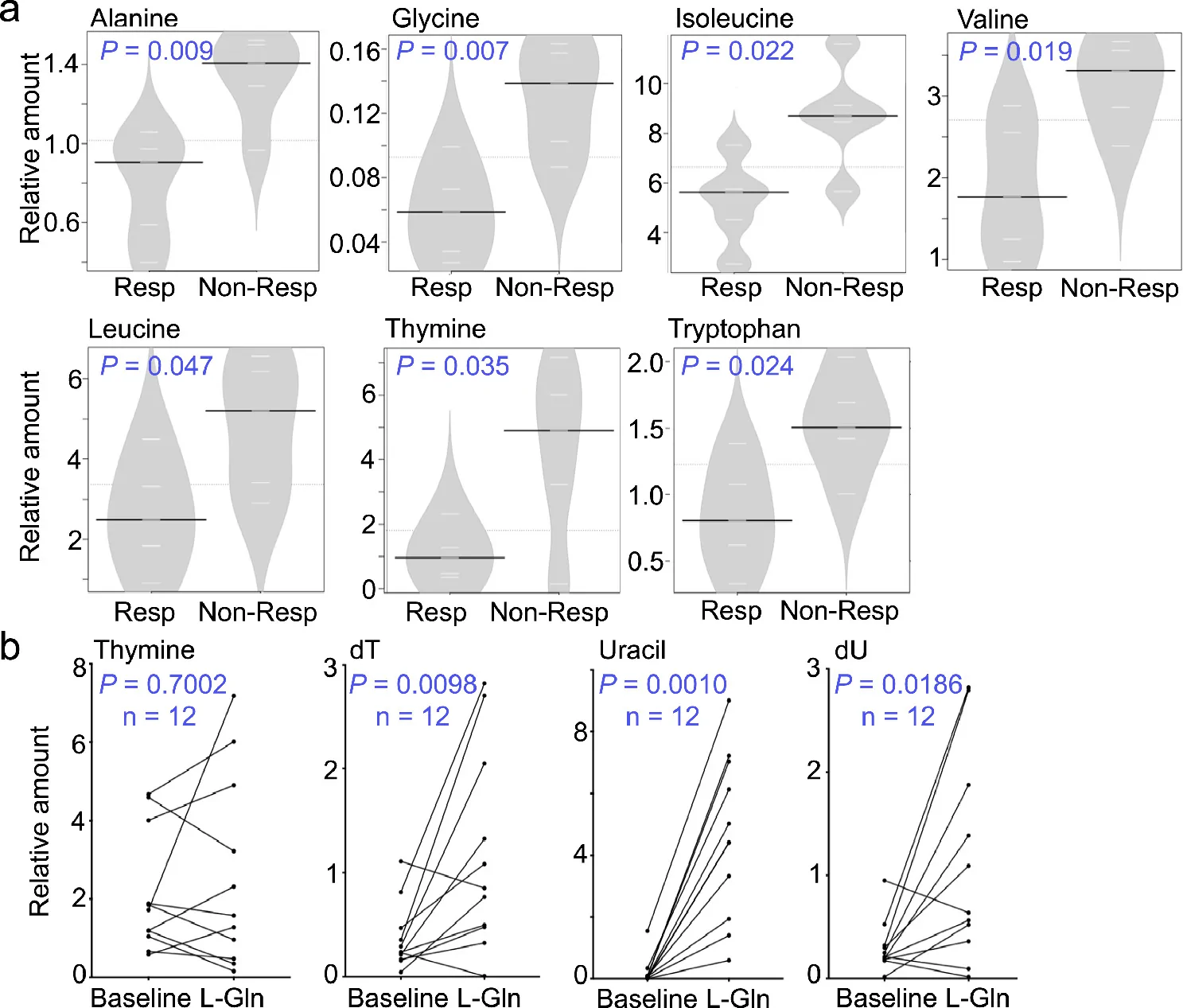
Stool metabolic analysis. **a**. Stool metabolomics were measured following one week of L-glutamine treatment, where differential amino acid enrichment is indicated, stratified for responders (Resp, n = 7) and non-responders (Non-Resp, n = 7). Responders were patients achieving CR or PR and non-responders as those having SD or PD. **b**. Pyrimidine base and nucleoside abundance was measured at baseline and following L-glutamine (L-Gln) treatment. P values determined by two-sided Student’s t-test for both **a**. and **b**. with *P* < 0.05 being statistically significant. Source data

Stool metabolite analysis demonstrated seven amino acids significantly elevated in the nonresponders compared to the responding subjects following l-glutamine administration, not found at baseline for the same participants (Extended Data Fig. 4a). In line with l-glutamine treatment, pyrimidine biosynthesis was generally elevated (Extended Data Fig. 4b). While gram-negative bacteria are described to disrupt the absorption of neutral amino acids^17^, the specific bacteria elevated in these subjects have not been described. The limiting of malabsorption by glutamine treatment in the responders would reasonably contribute to the observed weight stability and elevated chemotherapy responsivity (Supplementary Tables 7–9). An observed enrichment in gram-negative bacteria in those participants with shorter OS suggested the measurement of lipopolysaccharide (LPS) in circulation. l-Glutamine treatment improved gut barrier function, as evidenced by a significant reduction in LPS levels, even in the presence of GA for all subjects (Fig. 7a). However, the responders had reduced downstream pathogen-associated molecular pattern activation with related cytokines. Specifically, proinflammatory CCL11 and IL-33 were significantly lower in responders compared to nonresponders (Fig. 7b). The gut microbiome constitution at baseline and l-glutamine-driven effects on bacterial metabolism and glutamine intermediary metabolism could serve as the basis of efficacy of the combination therapy for further testing in a randomized control trial.

## Discussion

The phase 1 GlutaPanc trial met its primary endpoint, establishing the RP2D of the study combination at maximum doses of oral l-glutamine (30 g per day orally) daily with full standard doses of gemcitabine (1,000 mg m^−2^) and nab-paclitaxel (125 mg m^−2^) on days 1, 8 and 15 every 28-day cycle. The feasibility and safety in pairing l-glutamine supplementation with chemotherapy as a therapeutic combination in advanced PDAC is reassuring, given that historic efforts to antagonize glutamine uptake and metabolism as a therapeutic strategy in PDAC have proven challenging^18^. Our preliminary efficacy signals with the study combination support safe l-glutamine administration in combination with first-line GA chemotherapy, with encouraging tumor response rates and survival outcomes compared to historical findings reported in a pivotal phase 3 trial of first-line GA alone in metastatic PDAC^15^.

Our preplanned exploratory analyses highlight potential mechanisms underlying the efficacy of the study combination that warrant further validation. Firstly, l-glutamine administration elicited on-target effects on intermediary metabolism with resultant increased plasma glutamine levels and subsequent uptake into intermediary pathways following glutamine treatment, independent of GA chemotherapy. Interestingly, exogenous l-glutamine did not impact the overall gut microbiome composition in this study; however, given the understanding that enteric bacteria have relied on glutamine as a central nutrient for a variety of metabolic fates including ammonium assimilation or as a nitrogen donor in biosynthetic reactions and amino acid synthesis, we performed shotgun metagenomics to assess changes in bacterial genes^16,19^. l-Glutamine upregulated multiple metabolic pathways fulfilling key roles in bacterial growth and function, while enrichment in bacterial genes related to fatty acid β-oxidation and gluconeogenesis were observed in participants experiencing shorter OS and nonresponse to study treatment. These findings are hypothesis generating given that we observed higher abundance of gram-negative bacteria in the gut of short OS participants, which may favor greater upregulation in fatty acid oxidation over anaplerosis at baseline in these individuals, furthered by l-glutamine. However, in study treatment-responsive participants, l-glutamine treatment seemed to reduce enoyl CoA hydratase activity in multiple reactions required for fatty acid oxidation. Lastly, l-glutamine supplementation appears to preserve gut barrier integrity, limit malabsorption and inhibit proinflammatory response, providing yet another possible benefit through anticachexia effects that have been previously characterized with l-glutamine administration^12^. Collectively, our correlative studies suggest that intermediary metabolism, basal gut microbiome constitution and associated systemic inflammatory signature could serve as biomarkers for l-glutamine treatment.

Study limitations include the nonrandomized, single-arm design and small sample size. Our results do not support the claim that a combination of l-glutamine and GA is better than GA alone, as that question needs to be answered in the setting of a first-line randomized clinical trial. The safety and efficacy of combining l-glutamine with other systemic agents in PDAC also remain unknown. Additionally, the landscape for advanced PDAC is rapidly evolving with the advent of RAS inhibitors^20^. However, the pairing of l-glutamine with RAS inhibitors should not be prohibitive given the exquisite tolerability of l-glutamine, especially as RAS inhibitors in combination with GA are entering the first-line metastatic PDAC setting (NCT07491445). The positive phase 1 GlutaPanc trial represents a formal translation of l-glutamine supplementation as a therapeutic sensitizer. Maximum doses of oral l-glutamine with standard full doses of GA were achieved with a favorable safety profile of l-glutamine. The addition of oral l-glutamine to GA was associated with improved PFS, ORR and OS compared to historical levels of first-line GA alone in metastatic PDAC, albeit in a relatively small cohort^15^. Nevertheless, 15/16 (94%) experienced tumor shrinkage, while OS conclusions remain immature as five subjects are still alive with ongoing survival follow-up at the time of data cutoff. The combined findings of this report, along with the availability of a clinical-grade formulation of oral l-glutamine, further reinforce the attractiveness of this strategy as it advances into a randomized control trial.

## Methods

### Participant eligibility

This was a single-institution, open-label, single-arm, nonrandomized phase 1 trial enrolling participants with untreated advanced PDAC (defined as locally advanced, unresectable or metastatic disease) who were planned to receive standard GA chemotherapy. Participants aged ≥18 years with histologically confirmed advanced, unresectable or metastatic PDAC (de novo or recurrent disease) were eligible so long as systemic therapy was not received in the first-line metastatic setting (prior neoadjuvant or adjuvant chemotherapy and/or chemoradiation was allowed but must have been completed ≥6 months before recurrence). All inclusion and exclusion criteria for this trial are available in the study protocol. Of note, exclusionary criteria included parenteral nutrition or enteral feeding or use of any other nutritional supplement. The sex and/or gender of participants were determined through self-report. Eligible subjects with advanced PDAC were referred to our gastrointestinal (GI) oncology team at Cedars-Sinai Medical Center. Participants referred to our high-volume GI oncology clinic represent an economically, racially and ethnically diverse group of participants from within the Cedars-Sinai Medical Center health network and outside the network from providers in the community.

This study was approved by the Institutional Review Board of Cedars-Sinai Medical Center and informed consent was obtained from all subjects involved in the study. This study and its design were conducted according to the guidelines of the Declaration of Helsinki. This study was registered on ClinicalTrials.gov (NCT04634539) as of November 18, 2020.

### Study design and treatment

The full study protocol was previously published^14^; however, a copy has been included in Supplementary Information. A Bayesian adaptive design incorporating the EWOC algorithm was used for this phase 1 dose-finding trial^21^. The dose-finding portion of this study was preceded by an intended 7-day administration of l-glutamine alone (0.1, 0.2 or 0.3 g kg^−1^ oral twice daily). Following the l-glutamine lead-in phase for each subject, participants were enrolled to receive the combination of oral l-glutamine and GA across three different dosages for gemcitabine (600, 800 and 1,000 mg m^−2^ intravenous 30-min infusion on days 1, 8 and 15), nab-paclitaxel (75, 100 and 125 mg m^−2^ intravenously over 30 min on days 1, 8 and 15) and l-glutamine oral powder (0.1, 0.2 and 0.3 g kg^−1^ oral twice daily, rounded to nearest 5 g, with an upper limit of 15 g twice daily (30 g per day) for any dose level). Dose deescalations to GA were performed on the basis of toxicity, specifically DLTs. Escalation beyond standard maximum doses of GA were not permitted. However, deescalation to dose levels as low as 600 mg m^−2^ for gemcitabine and 75 mg m^−2^ for nab-paclitaxel were allowed in the event of toxicity. We prohibited further dose reductions below these lowest dose levels for GA given that they are unlikely to provide clinically meaningful benefit as observed in routine oncologic practice. No dose deescalations for l-glutamine were selected given the reassuring safety profile observed in phase 3 registrational trials^13^. Specifically, the most common (≥5% incidence) all-grade AEs to oral l-glutamine were constipation (25%), nausea (23%), headache (21%), vomiting (15%), pain in extremity (16%) and noncardiac chest pain (14%) in a population of participants with sickle cell anemia^13^. Each oral l-glutamine dose was to be immediately mixed with 240 ml of a beverage at cold or room temperature. l-Glutamine powder could also be consumed with 120–180 ml of food such as applesauce or yogurt, before ingestion.

Cancer phase 1 trials are typically designed to estimate the MTD or RP2D. As such, they are not designed to test a specific hypothesis or powered to favor a particular alternative hypothesis. The performance of such designs is evaluated using operating characteristics under different scenarios for the location of the true MTD. In our trial, the operating characteristics were reasonable for a sample size of 16, which established the sample size of 16 for this dose-finding phase 1 trial based on adaptive Bayesian design. A total of 18 subjects were enrolled into this phase 1 clinical trial; however, two of the subjects were deemed nonevaluable for the DLT assessment and were replaced with two new subjects, bringing the final cohort to *n* = 16. The dose-finding nature of this phase 1 trial where single participants are enrolled into various dosing combinations preclude replication; however, advancement of these positive findings into subsequent phase 2 or 3 trials would be warranted to confirm the safety and efficacy signals seen in this trial. No randomization or blinding was performed as this was a single-arm, phase 1 dose-finding trial.

The maximum daily dose of oral l-glutamine was selected at 15 g twice daily (30 g per day) in accordance with the FDA-recommended upper limit of 15 g twice daily (30 g per day) and based on the dose-limiting feature of oral glutamine being its solubility of only 3.6% at 23 °C and the fact that prior prospective trials found doses beyond 30 g per day prohibitive in its administration in persons with cancer^22–24^. Maximum doses of GA were selected according to FDA-approved standard doses as per the phase 3 registrational trial in the first-line treatment of metastatic PDAC^15^.

Notably, a 1-week lead-in period of l-glutamine alone was designed to ensure tolerability to twice-daily doses of oral l-glutamine. Here, if subjects were unable to tolerate ≥50% of the total dosing of oral l-glutamine over the lead-in period, they were replaced. At the conclusion of the l-glutamine lead-in phase, the first dose of GA was administered along with daily continuous dosing of l-glutamine. Preplanned fasted peripheral venous blood collections were performed during the morning hours (7–11 a.m.) at baseline (before the l-glutamine lead-in period), after completion of the l-glutamine lead-in period but before the first dose of GA (C1D1) and after the first dose of GA along with oral l-glutamine (C1D8). Stool samples were collected before and after the oral l-glutamine lead-in period as well. Blood and stool samples were processed according to standard operating procedure and stored at −80 °C until further use. Data collection and analysis were not performed blind to the conditions of the experiments for exploratory endpoints.

Dose reductions were permitted for GA as per the FDA label recommendations, while no dose modifications were permitted for oral l-glutamine. Treatment with the combination was continued until confirmed PD, intolerance or any discontinuation criteria were met.

### Endpoints and assessments

The primary endpoint in the phase 1 GlutaPanc trial was the RP2D, which was assessed on the basis of the number of DLTs, defined as the rate of treatment-related grade ≥3 AEs occurring within the first 4 weeks (that is, cycle 1) of study drugs. The dose level closest to the median of the posterior distribution of the MTD is defined as the RP2D, whereby the MTD is defined as the dose level such that the probability of DLTs at the MTD is *θ* = 0.33, calculated using statistical analysis programs R and JAGS under the discretion of the study biostatistician.

Secondary endpoints included safety and preliminary efficacy. All subjects entered into the study who received any amount of study treatment were included for safety analysis. Safety was assessed by AEs, as per the National Cancer Institute (NCI) Common Terminology Criteria for AEs (version 5.0). Clinical activity of the combination therapy was assessed by the objective response rate (including CR, PR, SD or PD), OS and PFS, which were evaluated every 8 weeks (that is, every two cycles ± 1 week) according to the revised Response Evaluation Criteria in Solid Tumors version 1.1 guidelines. Sex/gender-based analyses were not performed because of the nature of the phase 1 trial design with a limited cohort of participants to be enrolled precluding meaningful conclusions regarding sex/gender-driven factors. Reporting trial results was performed in accordance with CONSORT guidelines^25^.

### Stool metabolite extraction and analysis

Approximately 50 mg of wet-weight partially thawed stool samples were homogenized at cryotemperature for 30 s at 25 rpm using a CryoMill (Retsch) and a 6.5-mm YSZ grinding ball (Advanced Materials, 4039GM-S065). A 40:40:20 extraction solution of acetonitrile, methanol and water (high-performance liquid chromatography (HPLC)-grade; −20 °C) extraction solution was added at 1 ml per 50 mg. Following extraction, the cleared supernatants were evaluated by LC–mass spectrometry (MS) analysis. A small portion of each sample was used to generate a pool sample for quality controls testing every 12 samples.

The extract was resolved by a Luna NH_2_ 3-µm 100-Å (150 × 2.0 mm) column (Phenomenex) using a Vanquish Flex ultra-HPLC (Thermo Scientific) using mobile phases A (5 mM ammonium acetate pH 9.9) and B (95% acetonitrile) at a flow rate of 200 μl min^−1^. A linear gradient from 10% A to 95% A over 18 min was followed by 7 min of isocratic flow at 95% A and reequilibration to 10% A. Metabolites were detected with a Thermo Scientific Q Exactive MS instrument run with polarity switching (+3.5 kV/−3.5 kV) in full-scan mode using a range of 70–975 *m*/*z* and 70,000 resolution. Maven (version 8.1.27.11) was used to quantify the targeted polar metabolites by AreaTop, using the expected retention time and accurate mass measurements (<5 ppm) for identification. Data analysis was performed using in-house R scripts.

### Plasma metabolomics

Plasma amino acid concentrations were determined by MS in conjunction with a standard curve for each amino acid as described previously^26^. The samples were separated on a YMC Triart phenyl analytic column on an HPLC system using a mobile phase of 0.1% formic acid and 10 mM ammonium formate solution with a gradient of acetonitrile and water (95:5, v/v) at a flow rate of 0.40 ml min^−1^ for analysis using an LCMS-8060NX triple-quadrupole MS instrument (Shimadzu Corporation). Ratios of amino acid peak area per peak area of standards were plotted against concentration and calibration curves for each amino acid were generated by the 1/*x*^2^ weighted linear regression method using a software, LabSolutions Insight (Shimadzu). A total of 40 serial plasma samples were available from 15 subjects for the primary analysis of essential and nonessential amino acids, with 11 subjects having paired baseline and post-l-glutamine lead-in plasma collected.

All other metabolites measured were determined as a relative quantity from 50 µl of plasma extracted with 200 µl of methanol and ethanol (50:50, v/v). Metabolite extractions were analyzed with an Agilent 6470 A triple-quadrupole MS instrument, operating in negative mode, connected to an Agilent 1290 ultra-HPLC system. Changes in plasma metabolite levels were measured by relative area under the curve (AUC) by LC–MS or fold change in AUC to compare absolute value changes of plasma metabolites measured. Mobile phases consisted of HPLC-grade or LC–MS-grade reagents. Buffer A was water with 3% methanol, 10 mM TBA and 15 mM acetic acid. Buffers B and D were isopropanol and acetonitrile, respectively. Buffer C was methanol with 10 mM TBA and 15 mM acetic acid. The analytical column used was an Agilent ZORBAX RRHD Extend-C18 (1.8 µm, 2.1 × 150 mm) coupled with a ZORBAX Extend Fast Guard column for UHPLC Extend-C18 (1.8 µm, 2.1 mm × 5 mm). A total of 43 serial plasma samples were available from 15 subjects, with 13 subjects having complete sets of baseline, C1D1 and C1D8 plasma collected (one subject each failed to submit a C1D1 and C1D8 blood sample). Resulting chromatograms were visualized in Agilent MassHunter quantitative analysis for triple-quadrupole MS. The final peaks were manually checked for consistent and proper integration. Results were analyzed in MetaboAnalyst 6.0 (http://www.metaboanalyst.ca). Treatments were compared using the paired-samples Wilcoxon test (*P* < 0.05 being statistically significant). Correlation analyses were performed using Prism and Pearson’s *r* and *P* values (two-tailed) were calculated. Scatter plots were generated for select metabolites that showed a strong correlation (*P* < 0.05), with *r* > 0.7 or *r* < −0.7 indicating positive or negative correlations, respectively.

For generation of Kaplan–Meier OS curves for select plasma metabolites, plasma metabolite levels were stratified as high and low using the mean AUC measured for each metabolite by LC–MS and compared using the log-rank (Mantel–Cox) test in participants treated with the study combination. Long OS was defined by participants whose median OS surpassed the historical median OS (258 days) of first-line GA alone in metastatic PDAC^15^. Because of biospecimen availability, missing metabolite measurements were excluded from these comparisons.

### DNA extraction, shotgun sequencing and bioinformatics analysis

A total of 23 stool samples were collected from 12 subjects for shotgun metagenomics before and after l-glutamine therapy. Complete sets of pre-l-glutamine and post-l-glutamine lead-in stool samples were available in 11 subjects, with stool missing from five subjects overall. Around 100–250 mg of each fecal sample was used for microbial DNA extraction using the MagMAX microbiome ultra nucleic acid isolation kit on Thermo Fisher’s automated DNA extraction platform KingFisher Flex, following the manufacturer’s instructions. The resulting DNA was fragmented and barcoded using the Illumina DNA prep kit and sequenced on an Illumina NovaSeq X Plus system using 25B flowcells and a 2× 150-bp sequencing configuration with a target depth of 20 million paired-end sequences per sample^27^.

The raw sequence reads were processed using KneadData for quality filtering and removal of human-derived reads. Taxonomic composition and functional assessments were performed using MetaPhlAn4 to identify the bacterial species present in the samples and determine the compositional profiles of the microbial communities^28,29^. Next, HUMAnN3 was used to annotate bacterial genes^28^. The bacterial gene abundances were aggregated into metabolic pathways according to the MetaCyc pathway classifications^30^. Alpha diversity indices were estimated using the MetaPhlAn4 taxonomic data in R package phyloseq^31^. All visualizations were created using R ggplot2 package.

### LPS and cytokine analyses

LPS and cytokine concentrations were measured in a total of 43 serial plasma samples available from 15 subjects. A total of 13 subjects had complete sets of baseline, C1D1 and C1D8 plasma collected (one subject each failed to submit a C1D1 and C1D8 blood sample). A sandwich ELISA was performed using the LPS sandwich ELISA kit (LS-F17912, LifeSpan Biosciences) with controls, standards, blanks and samples, following tenfold dilution. Sample concentrations were calculated following standard curve generation using a four-parameter logistic regression and compared using the paired-samples Wilcoxon test (*P* < 0.05 being statistically significant).

A panel of 46 cytokines were analyzed using a multiplex bead-based immunoassay, Human XL Cytokine Luminex performance assay 46-plex fixed panel (LKTM014B, R&D Systems) for Luminex 200. Plasma samples were passively thawed, centrifuged at 16,000*g* for 4 min immediately before diluting twofold with R&D assay diluent RD6-65 and tested in duplicate. A seven-point standard curve across the 5-log dynamic range of the assays was included in the current assay design.

### Statistical analysis

#### Dose finding and efficacy

Upon enrollment of a total of 16 participants into the DLT assessment cohort, the RP2D was assessed by EWOC, an adaptive Bayesian design, using the dosing schema proposed in Supplementary Table 1. The sample size was not based on the power of one study. The operating characteristics of the EWOC algorithm for the true value of the MTD were evaluated under three scenarios. The operating characteristics of the algorithm for the true value of the MTD and a description of various summary statistics, including the percentage of MTD recommendation using 1,000 trial replicates, average percentage of DLTs across all trials, percentage of trials with an excessive DLT rate and percentage of participants allocated to each dose level, can be found in the full protocol. In short, in the scenario wherein the MTD was dose level 2, the percentage of MTD recommendation would be 61.3%, with 93.7% of the trial recommending either dose level 1 or dose level 2 as the true MTD. Most participants would be allocated to the MTD and the trial would be deemed very safe. According to the evaluation of all scenarios, we concluded that the design would perform well with a sample size of 16 participants. Successive cohorts of one participant were treated under this dose-finding design and the dose level for the first participant was dose level 0 (Supplementary Table 1). The dose for each subsequent subject was determined by the statistician such that the probability that the dose exceeds the MTD is equal to a prespecified value *α*, beginning at *α* = 0.4 with increases in increments of 0.05 until *α* = 0.5 (representing a value of compromise between the toxic side effects and therapeutic aspect of the agent). No dose skipping was permitted and the trial was to be terminated if the posterior probability of DLTs at dose level −2 exceeded *θ* = 0.8 or more. Upon study completion, the RP2D was estimated as the median of the marginal posterior distribution of the MTD, rounded to the nearest discrete dose level.

As the goal was to characterize the preliminary therapeutic efficacy of the combination of gemcitabine, nab-paclitaxel and l-glutamine, no formal hypothesis testing was conducted. PFS was defined as the start of study treatment to disease progression or death, whichever occurred first, while OS was defined as the time from study treatment initiation to death. Kaplan–Meier plots were used to depict survival outcomes, while safety and tumor response were characterized using descriptive statistics. Tumor shrinkage was defined as any reduction in tumor target lesions from baseline.

#### Microbiome analyses

To detect changes in alpha diversity indices between pre-l-glutamine and post-l-glutamine treatment groups over time, linear mixed models were constructed. These models included fixed effects of treatment and long versus short OS or long versus short PFS, with subject as a random effect, using the lmerTest package^32^. Long PFS and OS were defined by participants whose median PFS and OS surpassed the historical median PFS (167 days) and OS (258 days), respectively, of first-line GA alone in metastatic PDAC^15^. *P* values < 0.05 were considered statistically significant. Beta diversity was quantified using the Bray–Curtis dissimilarity metric and differences in community composition among samples were visualized using nonmetric multidimensional scaling ordination. The significance of beta diversity differences between groups was determined by permutational multivariate analysis of variance using the vegan R package. This analysis included fixed effects of treatment and long versus short OS or long versus short PFS, with subject as a random effect.

Differential abundances of microbial species, pathways and gene families were estimated using per-feature testing in MaAsLin2, implemented in the MaAsLin2 R package (version 1.18.0)^33^. Linear mixed-effects models were used to explore potential associations among abundances of bacterial species, pathways and gene families with a single clinical variable of interest (treatment, response status (CR or PR versus SD or PD), long versus short OS or long versus short PFS) as the fixed effect and subject as a random effect to account for repeated measures. Feature tables were normalized using total sum scaling and log-transformed before modeling. Associations with a false discovery rate (FDR)-adjusted *q* value ≤ 0.25 were considered statistically significant.

### Reporting summary

Further information on research design is available in the Nature Portfolio Reporting Summary linked to this article.

## Supplementary information

Supplementary Information Study protocol and statistical analysis plan.

Reporting Summary

Peer Review File

Supplementary Tables Supplementary Tables 1–9.

CONSORT checklist CONSORT 2025 editable checklist.

Supplementary Data Source Data Table 1, Figs. 1–7 and Extended Data Figs. 2–4

## Source data

Source Data Supplementary Tables 2 and 3 disaggregated by sex.

## Data Availability

All data needed to evaluate the conclusions in the paper are present in the paper and/or the Supplementary Information. The metabolomics data generated in this study were deposited to the MassIVE public repository under dataset identifier MSV000095856. The raw metagenomic sequencing data generated in this study were deposited to the National Center for Biotechnology Information Sequence Read Archive under BioProject PRJNA1463891. Additional data related to this paper may be requested from the authors. Deidentified data not published including study protocol are available for transfer following submission of a data transfer agreement (DTA) to the principal investigator (jun.gong@csmc.edu). Published data will be made available for as long as permitted within journal policies. Preserved but unshared data will be made available as long as permitted by the public repository and/or institutional policy. Proposals will be vetted by the Cedars-Sinai Medical Center Cancer Clinical Trials Office within a timeframe of 60 days. Investigators and institutions who consent to the terms of the DTA form, including but not limited to the use of these data for the purpose of a specific project and only for research purposes and to protect the confidentiality of the data and limit the possibility of identification of participants for the duration of the agreement, will be granted access. Source data are provided with this paper.

## Code availability

No custom code or mathematical algorithms were used to generate the results in this study.

## References

[1] Siegel, R. L., Giaquinto, A. N. & Jemal, A. Cancer statistics, 2024. *CA Cancer J. Clin.* **74**, 12–49 (2024). 10.3322/caac.21820 38230766

[2] Aprile, G. et al. Second-line chemotherapy for advanced pancreatic cancer: which is the best option?. *Crit. Rev. Oncol. Hematol.* **115**, 1–12 (2017). 10.1016/j.critrevonc.2017.03.025 28602164

[3] Cruzat, V., Macedo Rogero, M., Noel Keane, K., Curi, R. & Newsholme, P. Glutamine: metabolism and immune function, supplementation and clinical translation. *Nutrients* **10**, 1564 (2018). 10.3390/nu10111564 PMC6266414 30360490

[4] Son, J. et al. Glutamine supports pancreatic cancer growth through a KRAS-regulated metabolic pathway. *Nature* **496**, 101–105 (2013). 10.1038/nature12040 PMC3656466 23535601

[5] Qin, C. et al. Metabolism of pancreatic cancer: paving the way to better anticancer strategies. *Mol. Cancer* **19**, 50 (2020). 10.1186/s12943-020-01169-7 PMC7053123 32122374

[6] Klimberg, V. S. et al. Glutamine facilitates chemotherapy while reducing toxicity. *JPEN J. Parenter. Enteral Nutr.* **16**, 83s–87s (1992). 10.1177/014860719201600609 1287230

[7] Klimberg, V. S. et al. Effect of supplemental dietary glutamine on methotrexate concentrations in tumors. *Arch. Surg.* **127**, 1317–1320 (1992). 10.1001/archsurg.1992.01420110063013 1444793

[8] Chen, M. J. et al. Oral glutamine supplement inhibits ascites formation in peritoneal carcinomatosis mouse model. *Gastroenterol. Res. Pract.* **2013**, 814054 (2013). 10.1155/2013/814054 PMC3806239 24194753

[9] Guo, C. et al. SLC38A2 and glutamine signalling in cDC1s dictate anti-tumour immunity. *Nature* **620**, 200–208 (2023). 10.1038/s41586-023-06299-8 PMC10396969 37407815

[10] Ishak Gabra, M. B. et al. Dietary glutamine supplementation suppresses epigenetically-activated oncogenic pathways to inhibit melanoma tumour growth. *Nat. Commun.* **11**, 3326 (2020). 10.1038/s41467-020-17181-w PMC7335172 32620791

[11] Pan, M. et al. Regional glutamine deficiency in tumours promotes dedifferentiation through inhibition of histone demethylation. *Nat. Cell Biol.* **18**, 1090–1101 (2016). 10.1038/ncb3410 PMC5536113 27617932

[12] Muranaka, H. et al. Glutamine supplementation as an anticancer strategy: a potential therapeutic alternative to the convention. *Cancers (Basel)* **16**, 1057 (2024). 10.3390/cancers16051057 PMC10930819 38473414

[13] Niihara, Y. et al. A phase 3 trial of l-glutamine in sickle cell disease. *N. Engl. J. Med.* **379**, 226–235 (2018). 10.1056/NEJMoa1715971 30021096

[14] Gong, J. et al. Combination l-glutamine with gemcitabine and nab-paclitaxel in treatment-naive advanced pancreatic cancer: the phase I GlutaPanc study protocol. *Biomedicines* **11**, 1392 (2023). 10.3390/biomedicines11051392 PMC10216251 37239063

[15] Von Hoff, D. D. et al. Increased survival in pancreatic cancer with nab-paclitaxel plus gemcitabine. *N. Engl. J. Med.* **369**, 1691–1703 (2013). 10.1056/NEJMoa1304369 PMC4631139 24131140

[16] Forchhammer, K. Glutamine signalling in bacteria. *Front. Biosci.* **12**, 358–370 (2007). 10.2741/2069 17127304

[17] Yu, Y. et al. Changes to gut amino acid transporters and microbiome associated with increased E/I ratio in *Chd8*^+/−^ mouse model of ASD-like behavior. *Nat. Commun.* **13**, 1151 (2022). 10.1038/s41467-022-28746-2 PMC8894489 35241668

[18] Biancur, D. E. et al. Compensatory metabolic networks in pancreatic cancers upon perturbation of glutamine metabolism. *Nat. Commun.* **8**, 15965 (2017). 10.1038/ncomms15965 PMC5500878 28671190

[19] Perna, S. et al. The role of glutamine in the complex interaction between gut microbiota and health: a narrative review. *Int. J. Mol. Sci.* **20**, 5232 (2019). 10.3390/ijms20205232 PMC6834172 31652531

[20] Wolpin, B. M. et al. Daraxonrasib in previously treated advanced *RAS*-mutated pancreatic cancer. *N. Engl. J. Med.* **394**, 1790–1802 (2026). 10.1056/NEJMoa2505783 42090791

[21] Tighiouart, M. & Rogatko, A. Dose finding with escalation with overdose control (EWOC) in cancer clinical trials. *Stat. Sci.* **25**, 217–226 (2010).

[22] Kucuktulu, E., Guner, A., Kahraman, I., Topbas, M. & Kucuktulu, U. The protective effects of glutamine on radiation-induced diarrhea. *Support Care Cancer* **21**, 1071–1075 (2013). 10.1007/s00520-012-1627-0 23064902

[23] Jebb, S. A. et al. 5-Fluorouracil and folinic acid-induced mucositis: no effect of oral glutamine supplementation. *Br. J. Cancer* **70**, 732–735 (1994). 10.1038/bjc.1994.385 PMC2033386 7917930

[24] Daniele, B. et al. Oral glutamine in the prevention of fluorouracil induced intestinal toxicity: a double blind, placebo controlled, randomised trial. *Gut* **48**, 28–33 (2001). 10.1136/gut.48.1.28 PMC1728161 11115819

[25] Schulz, K. F., Altman, D. G., Moher, D. & Group, C. CONSORT 2010 statement: updated guidelines for reporting parallel group randomised trials. *BMJ* **340**, c332 (2010). 10.1136/bmj.c332 PMC2844940 20332509

[26] Harada, M., Karakawa, S., Yamada, N., Miyano, H. & Shimbo, K. Biaryl axially chiral derivatizing agent for simultaneous separation and sensitive detection of proteinogenic amino acid enantiomers using liquid chromatography–tandem mass spectrometry. *J. Chromatogr. A* **1593**, 91–101 (2019). 10.1016/j.chroma.2019.01.075 30739759

[27] Sato, M. P. et al. Comparison of the sequencing bias of currently available library preparation kits for Illumina sequencing of bacterial genomes and metagenomes. *DNA Res.* **26**, 391–398 (2019). 10.1093/dnares/dsz017 PMC6796507 31364694

[28] Beghini, F. et al. Integrating taxonomic, functional, and strain-level profiling of diverse microbial communities with bioBakery 3. *eLife* **10**, e65088 (2021). 10.7554/eLife.65088 PMC8096432 33944776

[29] Blanco-Miguez, A. et al. Extending and improving metagenomic taxonomic profiling with uncharacterized species using MetaPhlAn 4. *Nat. Biotechnol.* **41**, 1633–1644 (2023). 10.1038/s41587-023-01688-w PMC10635831 36823356

[30] Caspi, R. et al. The MetaCyc database of metabolic pathways and enzymes and the BioCyc collection of pathway/genome databases. *Nucleic Acids Res.* **42**, D459–D471 (2014). 10.1093/nar/gkt1103 PMC3964957 24225315

[31] McMurdie, P. J. & Holmes, S. phyloseq: an R package for reproducible interactive analysis and graphics of microbiome census data. *PLoS ONE* **8**, e61217 (2013). 10.1371/journal.pone.0061217 PMC3632530 23630581

[32] Kuznetsova, A., Brockhoff, P. B. & Christensen, R. H. B. lmerTest package: tests in linear mixed effects models. *J. Stat. Softw.* **82**, 1–26 (2017).

[33] Mallick, H. et al. Multivariable association discovery in population-scale meta-omics studies. *PLoS Comput. Biol.* **17**, e1009442 (2021). 10.1371/journal.pcbi.1009442 PMC8714082 34784344

