# Supplementary material for: l-Glutamine in combination with first-line gemcitabine and nab-paclitaxel in advanced pancreatic ductal adenocarcinoma: an open-label, single-arm, phase 1 GlutaPanc trial
Source: Nat Cancer. 2026 Aug 31;7(9):1406–18. doi: 10.1038/s43018-026-01225-z (PMC13600916; doi:10.1038/s43018-026-01225-z)
Supplement: Supplementary file 1 [file 43018_2026_1225_MOESM1_ESM.pdf]

nature cancer

Supplementary information
https://doi.org/10.1038/s43018-026-01225-z

L-Glutamine in combination with first-line gemcitabine and nab-paclitaxel in advanced pancreatic ductal adenocarcinoma: an open-label, single-arm, phase 1 GlutaPanc trial

In the format provided by the authors and unedited

Cedars Sinai

CANCER

IIT2020-02-GONG-GLUTAPANC

Phase I trial of first-line L-glutamine with gemcitabine and nab-paclitaxel in advanced pancreatic cancer (GlutaPanc)

Principal Investigator: **Jun Gong, MD**
Cedars-Sinai Medical Center
8700 Beverly Blvd, LL NT, AC 1042B
Los Angeles, CA 90048
T: 310-423-5776
F: 310-423-4759
E: jun.gong@cshs.org

Sub-Investigator(s)	Department/Division
Andrew Hendifar, MD	Medicine/Hematology and Oncology
Arsen Osipov, MD	Medicine/Hematology and Oncology
Neil Bhowmick, PhD	Medicine/Hematology and Oncology
Jennifer Van Eyk, PhD	Biomedical Sciences
David Hoffman, MD	Medicine/Hematology and Oncology
Jeremy Lorber, MD	Medicine/Hematology and Oncology
Kevin S. Scher, MD	Medicine/Hematology and Oncology

Biostatistician: Mourad Tighiouart, PhD
Associate Director, Biostatistics and Bioinformatics
Cedars Sinai Medical Center
T: 310-248-8673
E: Mourad.Tighiouart@cshs.org

FDA Status: IND Exempt

Funding Source: CSMC

ClincialTrials.gov Reg: Required

NCT Number: NCT04634539

ClincialTrials.gov Reporting: Not Required

Current Version: Protocol Version 3 dated 09SEPT021

Initial Version: Protocol Version 1 dated 14OCT2020

CONFIDENTIAL
This material is the property of Cedars-Sinai Medical Center. Do not disclose or use except as authorized in writing by the study sponsor.

Protocol Version 3: Dated 09SEP 2021

Signature Page

The signature below constitutes the approval of this protocol and the attachments, and provides the necessary assurances that this trial will be conducted according to all stipulations of the protocol, including all statements regarding confidentiality, and according to local legal and regulatory requirements and applicable U.S. federal regulations and ICH guidelines.

____ ________________

Principal Investigator (PI) Signature **Date**

CONFIDENTIAL
This material is the property of Cedars-Sinai Medical Center. Do not disclose or use except as authorized in writing by the study sponsor.

TABLE OF CONTENTS

LIST OF ABBREVIATIONS **1**

STUDY SCHEMA **2**

STUDY SUMMARY **3**

Primary Objectives: 3

Secondary Objectives 3

Exploratory Objectives 3

1.0 BACKGROUND AND RATIONALE **5**

1.1 Disease Background 5

1.2 Study Agent(s) Background and Associated Known Toxicities 5

1.3 Rationale 7

1.4 Correlative Studies 8

2.0 STUDY OBJECTIVES **9**

2.1 Primary Objective 9

2.2 Secondary Objectives 9

2.3 Exploratory Objectives 9

3.0 STUDY ENDPOINTS **9**

3.1 Primary Endpoint 9

3.2 Secondary Endpoints 9

3.3 Exploratory Endpoints 10

4.0 STUDY DESIGN **10**

5.0 PATIENT ELIGIBILITY **10**

5.1 Inclusion Criteria 11

5.2 Exclusion Criteria 12

6.0 TREATMENT PLAN **13**

6.1 Treatment Dosage and Administration 13
6.2 Definition of Dose-Limiting Toxicity 15
6.3 Toxicities and Dosing Delays/Dose Modifications 16
6.4 Concomitant Medications/Treatments 18
6.5 Duration of Study Participation 19
6.6 Removal of Patients from Protocol 19
6.7 Subject Replacement 19

7.0 STUDY PROCEDURES 19
7.1 Screening/Baseline Procedures 19
7.2 Procedures During Treatment 21
7.3 Follow-up Procedures 22
7.4 Time and Events Table 23
7.5 Removal of Subjects from Study 25

8.0 ADVERSE EVENTS (AE) 25
8.1 Adverse Event Monitoring 25
8.2 Definitions 26
8.3 Severity of Adverse Events 27
8.4 Reporting Requirements for Adverse Events 27

9.0 CORRELATIVES/SPECIAL STUDIES 29
9.1 Collection of Plasma Specimens 29
9.2 Sample Processing/Specimen Banking 30
9.3 Shipping/Plasma Metabolomics 31
9.4 Stool Microbiome Analyses 31

10.0 STATISTICAL CONSIDERATIONS 32
10.1 Sample Size and Randomization 32
10.2 Data Sets Analyzed 33
10.3 Data Analyses/Study Endpoints 33

10.4 Data Analysis Plans 34

11.0 STUDY MANAGEMENT 35

11.1 Conflict of Interest 35

11.2 Institutional Review Board (IRB) Approval and Consent 35

11.3 Registration Procedures 36

11.4 Data Management and Quality Control and Reporting 36

11.5 Data and Safety Monitoring 37

11.6 Record Retention 37

11.7 Adherence to Protocol 38

11.8 Obligations of Investigators 39

12.0 REFERENCES 41

13.0 APPENDICES 45

13.1 APPENDIX A: PROSPECTIVE CLINICAL TRIALS INVESTIGATING ORAL GLUTAMINE 45

13.2 APPENDIX B: EASTERN COOPERATIVE ONCOLOGY GROUP (ECOG) AND KARNOFSKY PERFORMANCE STATUS (KPS) SCALES 46

13.3 APPENDIX C: PLASMA METABOLOMICS LC/MS PROTOCOL 48

13.4 APPENDIX D: RESPONSE EVALUATION CRITERIA IN SOLID TUMORS (RECIST VERSION 1.1) 49

13.5 APPENDIX E: CYP3A4 and CYP2C8 INHIBITORS/INDUCERS 55

13.6 APPENDIX F: SUMMARY OF CHANGES 56

LIST OF ABBREVIATIONS

5-FU	5-flourouracil
AE	Adverse Event
ALT	Alanine Aminotransferase
ANC	Absolute Neutrophil Count
AST	Aspartate Aminotransferase
BUN	Blood Urea Nitrogen
CBC	Complete Blood Count
CMP	Comprehensive Metabolic Panel
CI	Confidence Interval
CR	Complete Response
CT	Computed Tomography
CTCAE	Common Terminology Criteria for Adverse Events
DLT	Dose Limiting Toxicity
DSMB	Data and Safety Monitoring Board
ECOG	Eastern Cooperative Oncology Group
EWOC	Escalation with Overdose Control
FDA	U.S. Food and Drug Administration
FOLFIRINOX	5-FU/Leucovorin, Irinotecan, and Oxaliplatin
H&P	History & Physical Exam
HR	Hazard Ratio
HRPP	Human Research Protections Program
IV (or iv)	Intravenously
MS	Mass Spectrometry
NCI	National Cancer Institute
ORR	Overall Response Rate
OS	Overall Survival
PD	Progressive Disease
PDAC	Pancreatic Ductal Adenocarcinoma
PFS	Progression Free Survival
p.o.	per os/by mouth/orally
PoCT	Point-of-care testing
PR	Partial Response
PS	Performance Status
RP2D	Recommended Phase 2 Dose
SAE	Serious Adverse Event
SCOEPS	Safety Committee On Early Phase Studies
SD	Stable Disease
SGOT	Serum Glutamic Oxaloacetic Transaminase
SPGT	Serum Glutamic Pyruvic Transaminase
TCA	Tricarboxylic Acid Cycle
WBC	White Blood Cells

STUDY SCHEMA

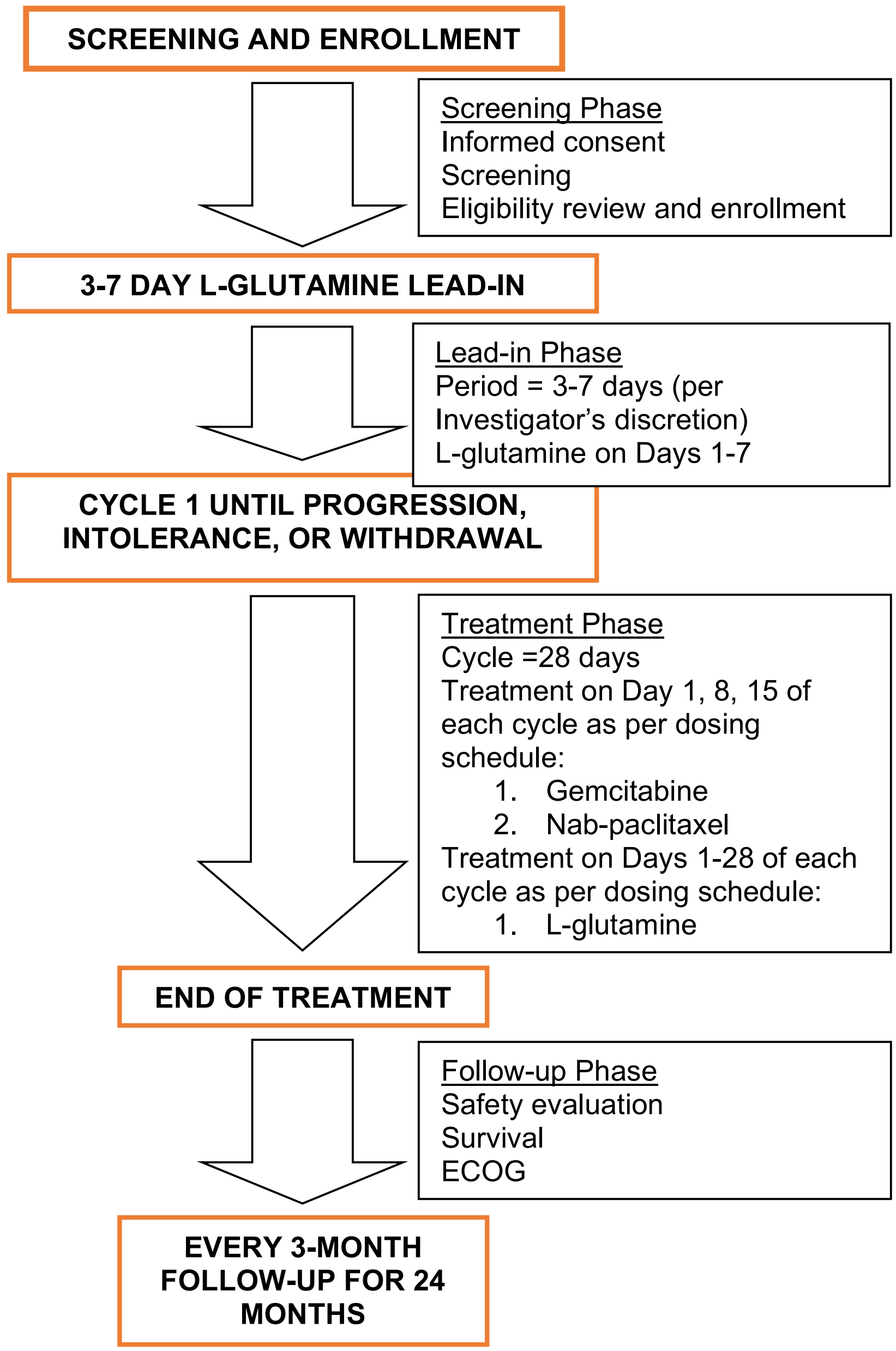

Primary endpoint: The number of dose-limiting toxicities (DLTs), defined as the rate of drug-related grade ≥3 adverse events (AEs) experienced within the first 4 weeks (1 cycle) of study treatment. The recommended phase 2 dose (RP2D) is defined as the dose level closest to the median of the posterior distribution of the maximum tolerated dose (MTD). The MTD is defined as the dose level such that the probability of DLT at the MTD is $\theta=0.33$.

STUDY SUMMARY

Title	Phase I trial of first-line L-glutamine with gemcitabine and nab-paclitaxel in advanced pancreatic cancer (GlutaPanc)
Protocol Number	IIT2020-02-Gong-GlutaPanc
Phase	I
Methodology	Single-arm, single-center
Study Duration	24 months
Study Center(s)	Cedars-Sinai Medical Center, Tower Hematology-Oncology
Objectives	**Primary Objectives:**<br>To determine the recommended phase II dose (RP2D) of combination gemcitabine, nab-paclitaxel, and L-glutamine in treatment-naïve metastatic pancreatic cancer<br><br>**Secondary Objectives**<br>- To describe the safety of gemcitabine, nab-paclitaxel and L-glutamine across all investigated dose levels in subjects with untreated advanced pancreatic cancer<br>- In patients with measurable disease, to describe any preliminary evidence of antitumor activity of the combination by assessment of objective response as determined by RECIST 1.1 criteria in subjects with treatment-naïve metastatic pancreatic cancer<br><br>**Exploratory Objectives**<br>- To determine the feasibility in measuring plasma metabolites in subjects across multiple timepoints to evaluate changes in plasma metabolites before and following study treatment<br>- To evaluate changes in the stool microbiome from before and after L-glutamine supplementation from available stool samples
Number of Subjects	16

Diagnosis and Main Inclusion Criteria	1. Advanced or unresectable, histologically confirmed pancreatic cancer (new diagnosis or recurrent) referred to Cedars-Sinai Medical Center (CSMC), Samuel Oschin Comprehensive Cancer Institute (SOCCI) for first-line chemotherapy<br>2. Age ≥18 years<br>3. ECOG performance status ≤2 or Karnofsky performance status ≥60% (**Appendix B**)<br>4. Demonstrate adequate organ and marrow function as defined (within 14 days of study treatment initiation) in **Table 1**<br>5. Have measurable disease based on RECIST 1.1<br>6. Female subjects of childbearing potential should have a negative urine or serum pregnancy within 72 hours prior to receiving the first dose of study medication. If the urine test is positive or cannot be confirmed as negative, a serum pregnancy test will be required.<br>7. Female subjects of childbearing potential should be willing to use adequate methods of birth control (hormonal or barrier method of birth control) or be surgically sterile or abstain from heterosexual activity for the course of the study through 120 days after the last dose of study medication. Subjects of childbearing potential are those who have not been surgically sterilized or have not been free from menses for >1 year.<br>8. Male subjects should agree to use an adequate method of contraception starting with the first dose of study therapy through 120 days after the last dose of study therapy.<br>9. Willingness to undergo serial peripheral blood draws during predefined study timepoints and under prespecified conditions (i.e., fasted, morning blood collections).<br>10. Written informed consent obtained from subject and ability for subject to comply with the requirements of the study.
Study Product(s), Dose, Route, Regimen	Gemcitabine 600, 800, or 1000 mg/m^2 IV over 30 minutes on Days 1, 8, and 15 every 28 days<br>Nab-paclitaxel 75, 100, or 125 mg/m^2 IV over 30 minutes on Days 1, 8, and 15 every 28 days<br>L-glutamine powder 0.1, 0.2, or 0.3 g/kg oral twice daily continuously (rounded to nearest 5 g, maximum daily dose of 15 g twice daily (30 g/day) for any dose level) with L-glutamine starting 1 week (+/- 1 day) prior to initiation of gemcitabine/nab-paclitaxel
Duration of administration	Until progression of disease, intolerance, or withdrawal by subject
Statistical Methodology	Single-institution, open-label, nonrandomized, single-arm, prospective phase I trial

1.0 BACKGROUND AND RATIONALE

1.1 Disease Background

Cytotoxic chemotherapy remains the preferred treatment for advanced or unresectable pancreatic cancer with combination regimens including 5-fluorouracil (5-FU), leucovorin, irinotecan, and oxaliplatin (FOLFIRINOX) or gemcitabine and nab-paclitaxel having recently been established as first-line standards in patients with good performance status (PS).[1] For those with poor PS, gemcitabine alone has remained a standard cytotoxic agent in the treatment of metastatic pancreatic cancer.[1] The administration rates of second-line and third-line chemotherapy in metastatic pancreatic cancer has approximated 45% and 21%, respectively, following failure of gemcitabine.[2] These values reinforce the importance of optimizing first-line chemotherapy given that the aggressive nature of advanced pancreatic cancer is underscored by progressive therapeutic resistance and a dismal 5-year survival rate of 3%.[3]

1.2 Study Agent(s) Background and Associated Known Toxicities

1.2.1 Preclinical Data

Preclinical investigations in human pancreatic ductal adenocarcinoma (PDAC) cells have shown that oncogenic *Kras* mutations reprogram glutamine metabolism through an alternative pathway to fuel the tricarboxylic acid (TCA) cycle and promote tumor growth.[4, 5] Early endeavors to antagonize glutamine metabolism as a therapeutic strategy in PDAC preclinical models, however, have largely failed.[6] Instead, a growing body of evidence supports that glutamine <u>deprivation</u> activates IκB kinase β (IKKβ) and p53, which in turn, promote cancer cell adaptation to glutamine depletion.[7, 8] In PDAC *in vitro* and *in vivo* models, microRNA-135 accumulates in response to glutamine deprivation in a p53- and reactive oxygen species (ROS)-dependent manner and contributes to cancer cell adaptation and survival.[9] Interestingly, one preclinical study demonstrated increased cancer cell survival in the face of low glutamine conditions whereas decreased cancer cell viability was observed when cells were cultured in higher concentrations of glutamine and the systemic agent PLX4032.[10] We have first-hand experience in targeting glutamine metabolism in human PDAC cells with the glutaminase inhibitor, CB-839, whereby blockade of glutamine uptake increased cancer cell survival and resistance to standard chemotherapy (gemcitabine, Fig 1A). When PDAC cells were treated with glutamine supplementation, however, there was enhancement of PDAC cell death with increasing concentrations of gemcitabine (Fig 1B). Lastly, glutamine supplementation decreased cancer cell viability with increasing concentrations of gemcitabine even in the face of glutaminase inhibition (Fig 1C). In vivo mouse models of PDAC have corroborated the antitumor effects of oral glutamine supplementation, when compared to controls, at 5 mg/kg per day doses of 100% glutamine, which are much lower doses than our supraphysiologic doses of 40 mM glutamine.[11]

Purpose and Hypothesis

This preclinical evidence provides the rationale for this single-institution, open-label, single-arm phase I trial investigating the feasibility of the combination of gemcitabine and nab-paclitaxel with L-glutamine in patients with unresectable or metastatic pancreatic cancer as a first-line therapy. We hypothesize that the combination of gemcitabine, nab-paclitaxel, and L-glutamine represents a feasible and attractive strategy to develop as a new therapeutic regimen in metastatic pancreatic cancer. We will also seek to characterize the preliminary efficacy of the combination and explore the changes in plasma metabolites resultant from study treatment across multiple timepoints to gain mechanistic insights and determine potential associations to efficacy with oral glutamine.

1.2.2 Clinical Data

Figure 1

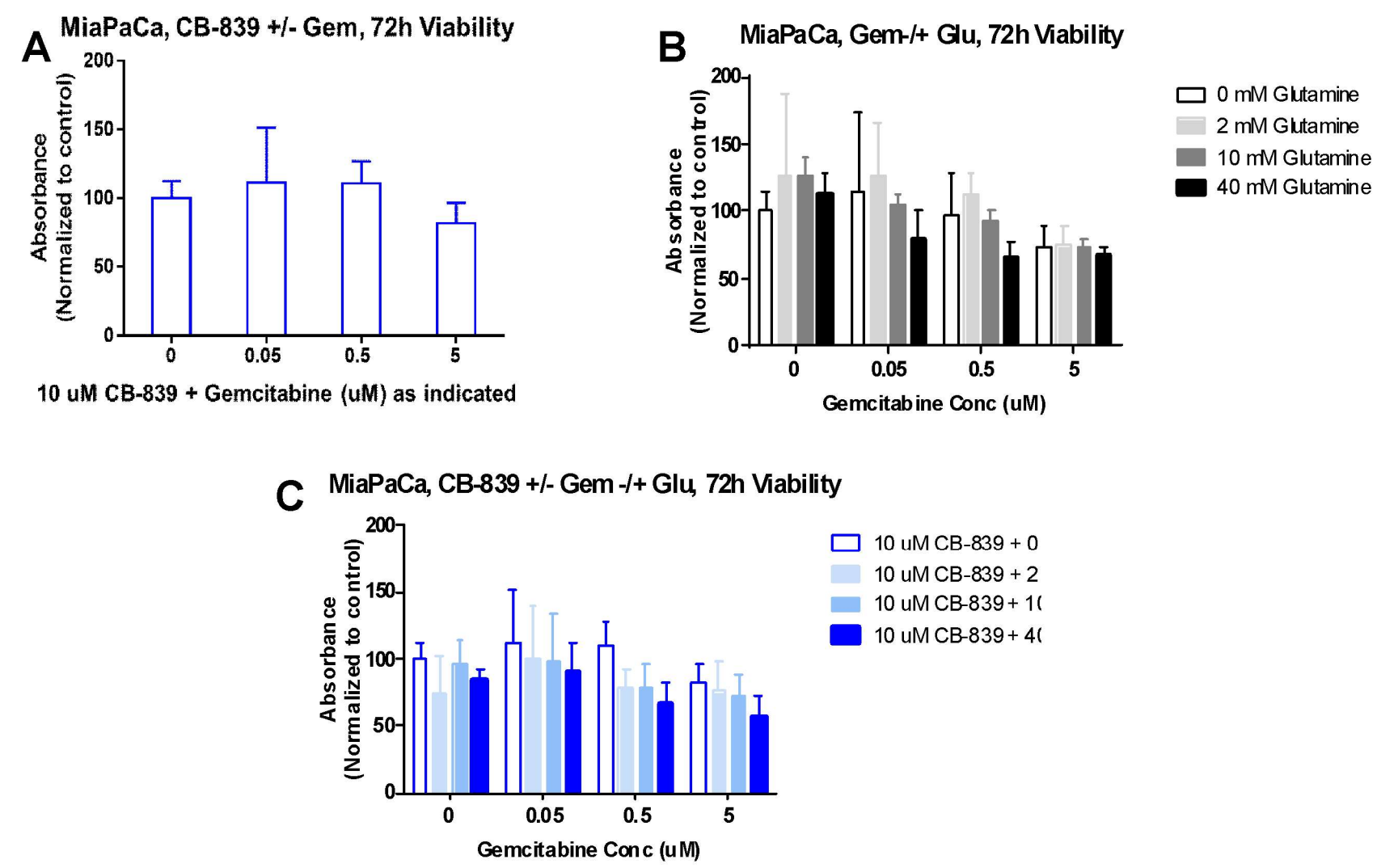

Figure 1. Glutamine supplementation enhances sensitivity of MIA PaCa-2 cells to gemcitabine. MIA PaCa-2 cells were treated for 72 hours with increasing concentrations of gemcitabine (μM, x-axis) and **A)** 10 μM CB-839 (glutaminase inhibitor), **B)** 0-40 mM glutamine, and **C)** 10 μM CB-839 with 0-40 mM glutamine. MIA PaCa-2 cells were cultured with RPMI, 10% FBS, P/S on Day 0. Viability was assessed using MTT reagent (M6494, Life Technologies) and normalized to control. Error bars are mean +/- SD. Average of 3 experiments each.

Gemcitabine is a pyrimidine nucleoside analog that was first approved by the U.S. Food and Drug Administration (FDA) in 1996 for the first-line treatment of metastatic pancreatic cancer based on a superior clinical benefit response (23.8%) over 5-FU (4.8%, p=0.0022).[12] In this randomized control trial of 126 patients with metastatic pancreatic cancer, gemcitabine produced a median overall survival (OS) of 5.65 months compared to 4.41 months with 5-FU (p=0.0025). In 2007, gemcitabine was also FDA approved in combination with the epidermal growth factor receptor erlotinib for the first-line treatment of advanced pancreatic cancer based on a phase III trial whereby gemcitabine and erlotinib demonstrated a modest, at best, OS benefit (median 6.24 months) over gemcitabine alone (5.91 months, hazard ratio (HR) of 0.82, 95% confidence interval (CI) 0.69-0.99, p=0.038).[13] More recently, the combination of gemcitabine and nab-paclitaxel (nanoparticle albumin-bound paclitaxel) was established as a new first-line treatment standard in metastatic pancreatic cancer based on the phase III MPACT trial.[14] Here, 861 patients with treatment-naïve, advanced pancreatic cancer were randomized 1:1 to receive gemcitabine and nab-paclitaxel or gemcitabine alone with the combination showing a superior OS benefit (median 8.5 months) over gemcitabine alone (6.7 months, HR 0.72, 95% CI 0.62-0.83, p<0.001). The median progression-free survival (PFS) was 5.5 months with gemcitabine and nab-paclitaxel compared to 3.7 months with gemcitabine (HR 0.69, 95% CI 0.58-0.82, p<0.001). The overall response rate (ORR) was higher with the combination as well (23% vs. 7%, p<0.001).

Under the FDA-labeled indication in untreated metastatic pancreatic cancer, gemcitabine can be administered by intravenous (IV) infusion over 30 minutes at 1000 mg/m^2 on days 1, 8, and 15 following nab-paclitaxel IV 30-minute infusion at 125 mg/m^2 on days 1, 8, and 15 every 28-day cycles.[15] The most commonly observed (incidence ≥20%) treatment-related adverse events (AEs) from gemcitabine and nab-paclitaxel in treated patients with pancreatic cancer have included neutropenia, fatigue, peripheral neuropathy, nausea, alopecia, peripheral edema, diarrhea, pyrexia, vomiting, decreased appetite, rash, and dehydration.

Over the past 3 decades, oral glutamine supplementation has been investigated in adult and pediatric patients with cancer treated with chemotherapy, radiation therapy, and stem cell transplantation as a means for mitigating mucositis, neuropathy, and radiation enteritis.[16-58] Other studies on glutamine intake have supported improvements in protein balance in tumor-bearing animals, enhancements in function of natural killer cells, and improvements in overall immunity and gut integrity with chronic glutamine supplementation.[59] L-glutamine, however, is currently only FDA approved as an oral powder administered twice daily based on body weight to reduce the acute complications of sickle cell disease based on a randomized phase III trial enrolling 230 patients with sickle cell anemia or sickle β^0-thalassemia.[60] Here, patients in the L-glutamine group had significantly fewer pain crises (median 3.0) than those in the placebo group (median 4.0, $p=0.005$). The most common adverse reactions (incidence >10%) from L-glutamine treatment in this population have included constipation, nausea, headache, abdominal pain, cough, pain in extremity, back pain, and chest pain.

Refer to the most current gemcitabine, nab-paclitaxel, and L-glutamine package inserts.

1.3 Rationale

This phase I clinical trial investigating the combination of gemcitabine, nab-paclitaxel, and L-glutamine represents the direct translation of preclinical findings generated by the Bhowmick and Gong labs illustrating the anticancer potential of glutamine when paired with standard chemotherapy in PDAC (i.e., gemcitabine). Although initial efforts to target the *Kras*-driven metabolic dependency of PDAC through glutamine antagonism in preclinical models have largely failed, [6] our work (unpublished) and the work of others have demonstrated that direct glutamine supplementation, rather than glutamine deprivation, can elicit antitumor effects in PDAC.[7-11] As such, we are seeking to test the feasibility of combining L-glutamine with gemcitabine and nab-paclitaxel in treatment-naïve patients with unresectable or metastatic PDAC. Gemcitabine and nab-paclitaxel represents a standard first-line therapy in advanced PDAC, however, optimizing the efficacy of first-line therapy is of high unmet need given the dismal overall prognosis and precipitous decline in receipt of second- and third-line chemotherapy regimens in this population.[2, 3]

Dosing levels for gemcitabine and nab-paclitaxel are based on standard dose levels as recommended by the FDA-labeled indication for this combination in metastatic PDAC.[15] L-glutamine has been used widely as an oral supplement for various indications for decades with no evidence of major side effects.[59] Proposed doses of oral L-glutamine is based on the only FDA-labeled indication for L-glutamine powder (Endari®) to date as per phase III trial data.[60, 61] In the preceding phase II study, L-glutamine was dosed based on body weight (0.3 g/kg/dose, rounded to nearest 10, 20, or 30 g/day) and adjusted in increments of 5 g with an upper limit of 30 g/day.[62] Our proposal to start L-glutamine 1 week before the initiation of gemcitabine/nab-paclitaxel is consistent with recent phase III data supporting this approach.[29] Across 47 prospective trials investigating enteral glutamine in over 1600 adult patients (including those treated with chemotherapy and radiation therapy), glutamine was well-tolerated without any definitive evidence of drug interactions or compromise in antitumor efficacy.[16-58, 60, 62-64] We therefore anticipate that L-glutamine will be safe at full FDA-labeled doses in combination with gemcitabine/nab-paclitaxel. Rather, the dose-limiting feature of oral glutamine is in its administration as the solubility of glutamine is only 3.6% at 23°C.[32] Consequently, doses of >15 g have historically required over 400 mL of fluid and constituted a significant burden to patients, who are often anorexic and would experience greater discomfort (nausea) with such continuous daily intake while on chemotherapy.[24, 32, 34] The FDA-approved doses of L-glutamine now permit administration of the powder in 240 mL of fluid per dose, which translates to about 500 mL of fluid intake daily.[61] In keeping with the constellation of prospective trial data whereby doses of oral glutamine have rarely, if at all, exceeded 30 g/day given that clinically-relevant larger volumes of water would be required to dissolve glutamine at higher doses, we have decided to cap the maximum daily dose of L-

glutamine in this study to the FDA-recommended upper limit of 15 g twice daily (30 g/day) for any dose level (**Appendix A**).

If we demonstrate the feasibility of L-glutamine with gemcitabine and nab-paclitaxel in this study, we would seek to advance the combination through a randomized trial of patients with metastatic pancreatic cancer in the first-line setting, a high-risk subgroup desperately in need of additional therapies that can improve upon the dismal outcomes seen in this population.

1.4 Correlative Studies

Preclinical studies have shown that oncogenic *Kras* alters glucose metabolism in PDAC such that glycolysis is enhanced to drive glycolytic intermediates into anabolic pathways important for proliferation.[65] For example, mutant *Kras* regulates key enzymes involved in glucose metabolism increasing glycolytic flux that drives glucose intermediates into 1.) the hexosamine biosynthesis pathway important for posttranslational protein modification and glycolipid and proteoglycan synthesis and 2.) the nonoxidative pentose phosphate pathway important for nucleic acid synthesis without affecting the flux of glycolytic metabolites into the TCA cycle. However, mutant *Kras* has been shown to alter glutamine metabolism to support tumor proliferation and growth as well through an alternative pathway to fuel the TCA cycle.[4, 5] Specifically, mutant *Kras* upregulates several enzymes resulting in transport of glutamine-derived aspartate into the cytoplasm that maintains cellular redox for ATP production through increasing the NADPH/$NADP^+$ ratio and generation of pyruvate.

We are in a unique position in this study to be able to investigate the metabolic changes elicited by oral L-glutamine with or without chemotherapy for the first time in human subjects with metastatic PDAC. Based on preclinical evidence of metabolic pathways altered by oncogenic *Kras* in PDAC, we hypothesize that 1.) L-glutamine supplementation can increase generation of pyruvate through an alternative pathway to fuel the TCA cycle involving a glutamine-derived aspartate shunt, which in turn, 2.) decreases glycolysis and glucose intermediates important for nucleic acid synthesis (via the nonoxidative pentose phosphate pathway) and protein modification and glycolipid/proteoglycan synthesis (via the hexosamine biosynthesis pathway) that altogether contribute to the anticancer activity of glutamine in PDAC.

We therefore will plan to collect peripheral venous blood at prespecified timepoints along the 3-7 day oral L-glutamine lead-in and dose-finding portion of this study when L-glutamine is combined with gemcitabine/nab-paclitaxel for measurement of changes in plasma metabolites to assess the impact of L-glutamine as well as combination therapy on metabolic pathways inclusive of mitochondrial oxidative phosphorylation, nonoxidative pentose phosphate, and hexosamine biosynthesis pathways. In an earlier study, 6 healthy male subjects were administered 0, 0.1, or 0.3 g/kg of oral glutamine weekly for 3 doses with blood samples collected at multiple timepoints after glutamine administration.[58] Oral glutamine resulted in significant increases in concentrations of amino acids that are known end products of glutamine metabolism including arginine, alanine, citrulline, and glutamate, while levels of glycine, methionine, phenylalanine, leucine, and branched-chain amino acids decreased as glutamine dose increased. *Given that Kras-driven metabolic reprogramming in PDAC has been shown to affect a multitude of pathways, however, we are proposing a broader pilot analysis using mass spectrometry (MS)-based quantitation of 219 central carbon metabolites involved in mitochondrial oxidative phosphorylation, fatty acid metabolism, amino acid metabolism, and lipid biosynthesis pathways.* L-glutamine has also been implicated in promoting gut health.[66] To this end, we will propose to evaluate changes in the stool microbiome before and after L-glutamine lead-in. The goal will be to identify bacteria whose abundance may be impacted by L-glutamine present in the gut. Abundance of specific bacteria will then correlated to rates of gastrointestinal side effects to chemotherapy.

Our correlative studies will thus assess the feasibility of performing serial plasma metabolomics in human PDAC subjects with metastatic disease and provide potential mechanistic insights into the anticancer properties of L-glutamine that may rely on metabolic pathways altered by mutant *Kras*. From our correlative analyses, we also hope to develop hypothesis-generating findings on potential associations between metabolic profiles associated with therapeutic response (or lack of) to study treatment. We will also aim to deduce a relationship between L-glutamine and abundance of specific bacterial species in the gut microbiome of human PDAC subjects from available stool samples.

2.0 STUDY OBJECTIVES

2.1 Primary Objective

To determine the recommended phase II dose (RP2D) of combination gemcitabine, nab-paclitaxel, and L-glutamine in treatment-naïve metastatic pancreatic cancer

2.2 Secondary Objectives

2.2.1 To describe the safety of gemcitabine, nab-paclitaxel and L-glutamine across all investigated dose levels in subjects with untreated advanced pancreatic cancer

2.2.2 In patients with measurable disease, to describe any preliminary evidence of antitumor activity of the combination by assessment of objective response rate, progression-free survival, and overall survival as determined by RECIST 1.1 criteria in subjects with treatment-naïve metastatic pancreatic cancer

2.3 Exploratory Objectives

2.3.1 To determine the feasibility in measuring plasma metabolites in subjects across multiple timepoints to evaluate changes in plasma metabolites before and following study treatment

2.3.2 To evaluate changes in the stool microbiome from before and after L-glutamine supplementation from available stool samples

3.0 STUDY ENDPOINTS

3.1 Primary Endpoint

3.1.1 The number of dose-limiting toxicities (DLTs), defined as the rate of drug-related grade ≥3 adverse events (AEs) experienced within the first 4 weeks (1 cycle) of study treatment. The RP2D is defined as the dose level closest to the median of the posterior distribution of the maximum tolerated dose (MTD). The MTD is defined as the dose level such that the probability of DLT at the MTD is $\theta=0.33$.

3.2 Secondary Endpoints

3.2.1 Adverse events as summarized in terms of type and severity by NCI CTCAE version 5.0

3.2.2 Clinical activity of the combination including objective response rate (ORR), progression-free survival (PFS), and overall survival (OS) evaluated every 8 weeks (every 2 cycles ±1 week) according to the revised RECIST guidelines (version 1.1)

3.3 Exploratory Endpoints

3.3.1 Plasma metabolite levels as assessed by mass spectrometry (MS)-based quantitation of blood samples collected from enrolled subjects at baseline, after a 1-week administration of L-glutamine, weekly throughout the first cycle of combination gemcitabine/nab-paclitaxel and L-glutamine, and upon disease progression, intolerance to therapy, or other reason for study discontinuation

3.3.2 Relative bacterial abundance as assessed by shotgun metagenomics from available stool samples collected from enrolled subjects at baseline and after L-glutamine lead-in prior to initiation of gemcitabine/nab-paclitaxel

4.0 STUDY DESIGN

This is a single-institution, open-label, nonrandomized, single-arm, prospective phase I trial with the purpose to investigate the feasibility of combination gemcitabine, nab-paclitaxel, and L-glutamine in treatment-naïve, unresectable or metastatic pancreatic cancer. We hypothesize that combining L-glutamine with gemcitabine and nab-paclitaxel represents a feasible and attractive strategy to enhance sensitivity to gemcitabine-based chemotherapy in metastatic PDAC based on preclinical rationale whereby glutamine supplementation increased anticancer activity with gemcitabine in PDAC cells. We will also plan to characterize the safety and preliminary efficacy of the combination in this cohort. Lastly, we propose to conduct plasma metabolomics in longitudinal samples collected from L-glutamine-treated subjects with and without gemcitabine/nab-paclitaxel to measure changes in a comprehensive number of metabolic pathways that may be altered by oncogenic *Kras* to identify potential mechanistic insights and associations with response to study treatment. We will also evaluate changes in stool microbiome before and after L-glutamine to investigate the impact of specific gut bacteria on promoting gut health from available stool samples.

Please refer to the Study Schema and Time and Events Table for a summary of study details and procedures.

5.0 PATIENT ELIGIBILITY

5.1 Inclusion Criteria

5.1.1 Advanced or unresectable, histologically confirmed pancreatic cancer (new diagnosis or recurrent, where recurrent tumor does not need to be histologically-confirmed) referred to Cedars-Sinai Medical Center (CSMC), Samuel Oschin Comprehensive Cancer Institute (SOCCI) for first-line chemotherapy. Prior neoadjuvant or adjuvant chemotherapy and/or chemoradiation is allowed but must have been completed ≥12 months prior to recurrence.

5.1.2 Age ≥18 years

5.1.3 ECOG performance status ≤2 or Karnofsky performance status ≥60% (**Appendix B**)

5.1.4 Demonstrate adequate organ and marrow function as defined below (within 14 days of study treatment initiation) in **Table 1**:

Table 1. Adequate Organ Function Laboratory Values

System	Laboratory Value
**Hematological**	
Absolute neutrophil count (ANC)	≥1,500 /µL
Platelets	≥100,000 / µL
Hemoglobin	≥9 g/dL or ≥5.6 mmol/L
**Renal**	
Serum creatinine or PoCT creatinine	≤1.5 X upper limit of normal (ULN) **OR** measured or calculated creatinine clearance ≥45 mL/min/1.73 m^2 for subject with creatinine levels > 1.5 X institutional ULN
**Hepatic**	
Serum total bilirubin	≤ 1.5 X ULN **OR** Direct bilirubin ≤ ULN for subjects with total bilirubin levels > 1.5 ULN
AST (SGOT) and ALT (SGPT)	≤ 5 X ULN

5.1.5 Have measurable disease based on RECIST 1.1

5.1.6 Female subject of childbearing potential should have a negative urine or serum pregnancy within 14 days prior to receiving the first dose of study medication for eligibility verification. If the urine test is positive or cannot be confirmed as negative, a serum pregnancy test will be required. Note: Negative urine or serum pregnancy test is also conducted within 72 hours prior to C1D1 for study procedures and should not preclude eligibility verification, but if screening pregnancy test is done within 72 hours of C1D1, it is not required to be repeated.

5.1.7 Female subjects of childbearing potential should be willing to use adequate methods of birth control (hormonal or barrier method of birth control) or be surgically sterile, or abstain from heterosexual activity for the course of the study through 120 days after the last dose of study medication. Subjects of childbearing potential are those who have not been surgically sterilized or have not been free from menses for > 1 year.

5.1.8 Male subjects should agree to use an adequate method of contraception starting with the first dose of study therapy through 120 days after the last dose of study therapy.

5.1.9 Willingness to undergo serial peripheral blood draws and provide stool samples during predefined study timepoints and under prespecified conditions (i.e., fasted, morning blood collections).

5.1.10 Written informed consent obtained from subject and ability for subject to comply with the requirements of the study.

5.2 Exclusion Criteria

5.2.1 Is currently participating in or has participated in a study of an investigational agent or using an investigational device within 4 weeks of the first dose of treatment.

5.2.2 Has previously received chemotherapy for metastatic disease (treatment with neoadjuvant or adjuvant therapy is allowed so long as treatment was completed ≥ 12 months prior to recurrence).

5.2.3 Has pre-existing grade ≥3 neuropathy.

5.2.4 Has a known additional malignancy that is progressing or requires active treatment. Exceptions include basal cell carcinoma of the skin, squamous cell carcinoma of the skin, or in situ cervical cancer that has undergone potentially curative therapy.

5.2.5 Has a known hypersensitivity to any components of the study drugs.

5.2.6 Has known active central nervous system (CNS) metastases and/or carcinomatous meningitis. Subjects with previously treated brain metastases may participate provided they are stable (without evidence of progression by imaging for at least four weeks prior to the first dose of trial treatment and any neurologic symptoms have returned to baseline), have no evidence of new or enlarging brain metastases, and are not using steroids for at least 7 days prior to trial treatment.

5.2.7 Has a history or current evidence of any condition, therapy, or laboratory abnormality that might confound the results of the trial, interfere with the subject's participation for the full duration of the trial, or is not in the best interest of the subject to participate, in the opinion of the treating investigator.

5.2.8 Has known psychiatric or substance abuse disorders that would interfere with cooperation with the requirements of the trial.

5.2.9 Is pregnant or breastfeeding or expecting to conceive or father children within the projected duration of the trial, starting with the pre-screening or screening visit through 120 days after the last dose of trial treatment.

5.2.10 Has any gastrointestinal disorder (e.g., bowel obstruction) or neurologic condition (e.g., oropharyngeal dysphagia) that may result in impairment of oral intake and/or absorption of study drug in the opinion of the treating investigator.

5.2.11 Is currently receiving any parenteral nutrition or enteral (tube) feeding or is planning to use any other nutritional supplement (defined as any other nutritional supplement containing *any* amount of glutamine) during the study period.

Table 2. Dosing Levels[a]

Dose Level	Gemcitabine Dosing	Nab-paclitaxel Dosing	L-glutamine Dosing[b]
-2	600 mg/m^2 IV D1, 8, 15	75 mg/m^2 IV D1, 8, 15	0.1 g/kg oral BID
-1	800 mg/m^2 IV D1, 8, 15	100 mg/m^2 IV D1, 8, 15	0.1 g/kg oral BID
0	1000 mg/m^2 IV D1, 8, 15	125 mg/m^2 IV D1, 8, 15	0.1 g/kg oral BID
1	1000 mg/m^2 IV D1, 8, 15	125 mg/m^2 IV D1, 8, 15	0.2 g/kg oral BID
2	1000 mg/m^2 IV D1, 8, 15	125 mg/m^2 IV D1, 8, 15	0.3 g/kg oral BID

[a]Every 28-day cycles, [b]Rounded to nearest 5 g, upper limit of 15 g twice daily (30 g/day) for any dose level; started 1 week (+/- 1 day) prior to chemotherapy

5.2.12 Patients on strong CYP2C8 or CYP3A4 inhibitors or inducers within 1 week prior to starting nab-paclitaxel (**Appendix E**).

6.0 TREATMENT PLAN

6.1 Treatment Dosage and Administration

This is a single-arm, open-label, phase I trial to determine the safety and feasibility of the combination of L-glutamine, gemcitabine, and nab-paclitaxel in untreated, metastatic pancreatic cancer.

Once patient is deemed eligible to be enrolled onto the study, the subject will proceed to Baseline for a 3–7-day lead-in, per Investigator's discretion, where patients will receive L-glutamine (0.1, 0.2, or 0.3 g/kg oral twice daily).

Proposed doses of oral L-glutamine in this study are based on the only FDA-labeled indication for L-glutamine powder (Endari®) to date as per phase III trial data and the preceding phase II study whereby L-glutamine dosing levels of 0.1, 0.2, or 0.3 g/kg/dose (rounded to nearest 5 g) with an upper limit of 30 g/day were used.[60-62] The dose-finding portion of this study for the combination of L-glutamine, gemcitabine, and nab-paclitaxel will be preceded by 3-7 day administration of L-glutamine (0.1, 0.2, or 0.3 g/kg oral twice daily), which is consistent with recent phase III data supporting this approach.[29] This 3-7 day administration of L-glutamine will facilitate measurement of baseline and post-glutamine monotherapy plasma metabolite levels prior to addition of gemcitabine and nab-paclitaxel.

Across 47 prospective trials investigating enteral glutamine in >1600 adult patients (including those treated with chemotherapy and radiation therapy), glutamine was well-tolerated without any definitive evidence of drug interactions or compromise in antitumor efficacy.[16-58, 60, 62-64] We therefore anticipate that L-glutamine will be safe at full FDA-labeled doses in combination with standard doses of gemcitabine/nab-paclitaxel as proposed in **Table 2**. Dosing levels for gemcitabine and nab-paclitaxel are based on standard dose levels as recommended by the FDA-labeled indication for this combination in metastatic PDAC.[15]

The dose-limiting feature of oral glutamine is its administration as the solubility of glutamine is only 3.6% at 23°C.[32] As a result, doses of >15 g have historically required over 400 mL of fluid and constituted a significant burden to patients, who are often anorexic and would experience greater discomfort (nausea) with such continuous daily intake while on chemotherapy.[24, 32, 34] The FDA-approved doses of L-glutamine now

permit administration of the powder in 240 mL of fluid per dose, which translates to about 500 mL of fluid intake daily.[61] We have decided to cap the maximum daily dose of L-glutamine in this study to the FDA-recommended upper limit of 15 g twice daily (30 g/day) for this reason, which is consistent with the constellation of prospective trial data whereby doses of oral glutamine have rarely, if at all, exceeded 30 g/day given that clinically-relevant larger volumes of water would be required to dissolve glutamine at higher doses (**Appendix A**).

This phase I study is designed to identify the maximum tolerated dose (MTD) using 5 dose levels as defined in **Table 2**. The MTD is defined as the dose level such that the probability of DLT at the MTD is $\theta=0.33$. We will use an extension of escalation with overdose control (EWOC) algorithm to design the trial.[67] The first patient will receive study drug at dose level 0 and the dose for the next patient will be determined according to the EWOC algorithm (Section 10.0). At any time during the trial, a patient may be enrolled to the trial if there are no more than 2 patients with unresolved DLT status in the trial. In other words, if there are 3 patients under treatment that did not experience DLT and have not finished their cycle 1 therapy, then we cannot enroll another patient.

Evaluation of the RP2D and DLTs will be based on the first 4 weeks (Cycle 1) of study drug treatment. The RP2D is defined as the dose level closest to the median of the posterior distribution of the MTD.

One treatment cycle of the combination of L-glutamine, gemcitabine, and nab-paclitaxel will consist of L-glutamine continuous oral daily dosing along with gemcitabine and nab-paclitaxel IV on days 1, 8, and 15 (every 28-day cycles) each given according to the dosing scheme (**Table 2**). A cycle will be defined around the start of gemcitabine and nab-paclitaxel i.e. Day 1 of each 28-day Cycle of gemcitabine and nab-paclitaxel. Treatment will be administered on an outpatient basis.

Post-treatment imaging and follow up will be performed 8 weeks after initiating therapy (C1D1). Patients removed from study for unacceptable adverse events will be followed until resolution or stabilization of the adverse event. Subjects who cannot complete a minimum of 3 days during the 3-7 day lead-in of L-glutamine alone may be replaced per Investigator's discretion. The start of a subsequent cycle can be delayed depending on toxicities of the prior cycle (Refer to Section 6.3).

Reported AEs and potential risks for L-glutamine, gemcitabine, and nab-paclitaxel are described in Section 6.3. No investigational or commercial agents or therapies other than those described below may be administered with the intent to treat the patient's malignancy.

Refer to the most current gemcitabine, nab-paclitaxel, and L-glutamine package inserts.

6.1.1 L-glutamine

The dose levels of L-glutamine in this study will be 0.1, 0.2, and 0.3 g/kg oral twice daily (rounded to nearest 5 g with an upper limit of 15 g twice daily (30 g/day) for any dose level) taken at the same time each day until confirmed progressive disease (PD), intolerance, or any discontinuation criteria are met. L-glutamine will be administered for 3-7 days prior to addition of gemcitabine/nab-paclitaxel. As per FDA recommendations, each dose of L-glutamine should be mixed immediately before ingestion with 8 oz. (240 mL) of a cold or room temperature beverage, such as water, milk or apple juice, or 4 oz. (120 mL) to 6 oz. (177 mL) of food such as applesauce or yogurt.[61] L-glutamine is formulated in 5 gram packets as a white crystalline powder in paper-foil-plastic laminate. Complete dissolution is not required prior to administration. If a dose of L-glutamine is accidentally skipped, the dose should be taken at the next scheduled dose at the regular time. Do not make up a skipped dose. In instances of vomiting following a dose of L-

glutamine, do not take an additional dose or double the dose. Instead, resume L-glutamine at the next scheduled dose. Subjects who cannot complete a minimum of 3 days of the 3-7 day lead-in of L-glutamine may be replaced, per Investigator's discretion. (See Section 6.7 Subject Replacement). After the lead-in period, oral compliance for l-glutamine is 85%. Less than 85% compliance is considered a deviation reportable to the IRB. No pre-medication is needed with L-glutamine administration. Supportive care medication such as antiemetics may be administered at the discretion of the treating investigator. The preparation and administration of L-glutamine is detailed in the pharmacy manual and/or package insert. A dosing diary and instructions will be provided for each enrolled subject and review by study staff is required at each clinical visit. L-glutamine is supplied and will be delivered by Emmaus Medical, Inc.

6.1.2 Gemcitabine

Gemcitabine will be administered (Refer to **Table 2**) at 600, 800, or 1000 mg/m^2 IV over 30 minutes on days 1, 8, and 15 (every 28-day cycles) until confirmed PD, intolerance, or any discontinuation criteria are met. Gemcitabine will be administered immediately after the infusion of nab-paclitaxel. Pre-medication may be administered per institutional standard practice at the investigator's discretion with gemcitabine. The preparation and administration of gemcitabine is detailed in the pharmacy manual and/or package insert. Gemcitabine is commercially available and will be obtained locally.

6.1.3 Nab-paclitaxel

Nab-paclitaxel will be administered (Refer to **Table 2**) at 75, 100, or 125 mg/m^2 IV over 30 minutes on days 1, 8, and 15 (every 28-day cycles) until confirmed PD, intolerance, or any discontinuation criteria are met. Nab-paclitaxel will be given first, before infusion of gemcitabine. Pre-medication for nab-paclitaxel may be administered per institutional standard practice at investigator's discretion. Grade 1-2 hypersensitivity reactions have occurred on days of nab-paclitaxel administration consisting of dyspnea (1%) and flushing, hypotension, chest pain, and arrhythmia (all <1%).[15] Premedication may be needed in patients who have had prior hypersensitivity reactions to nab-paclitaxel. Patients who experience a severe hypersensitivity reaction to nab-paclitaxel should not be re-challenged with this drug and will be discontinued from the study. The preparation and administration of nab-paclitaxel is detailed in the pharmacy manual and/or package insert. Nab-paclitaxel is commercially available and will be obtained locally.

6.1.4 Supportive Care

With the exception of prohibited therapies listed in Concomitant Medications/Treatments below, participants should receive appropriate supportive care measures as deemed necessary by the treating investigator per institutional standards.

Patients exhibiting hypomagnesaemia, hypocalcemia, and/or hypokalemia, should receive electrolyte replacement as indicated by national and institutional guidelines and be followed until laboratory values have normalized or as clinically indicated.

Supportive medications or treatments (e.g., acetaminophen or diphenhydramine) may be administered as deemed necessary by the treating investigator to provide adequate prophylactic or supportive care. Best supportive care (including antibiotics, growth factor support, correction of metabolic disorders, optimal symptom control, and pain management [including palliative radiotherapy, etc.]) should be used when necessary for all patients so long as all eligibility criteria are still fulfilled.

6.2 Definition of Dose-Limiting Toxicity

The following toxicities will be considered dose limiting in the event they are treatment-related and occur in the first 28 days of treatment (Cycle 1):

- Grade 4 thrombocytopenia of any duration or Grade 3 thrombocytopenia associated with a Grade 3 hemorrhage
- Grade 4 (life-threatening) anemia
- Grade ≥3 febrile neutropenia
- Absolute neutrophil count (ANC) < 100 mcL for ≥ 3 days
- ANC <500 mcL for ≥ 7 days
- Any grade 3-4 non-hematological toxicity considered clinically significant by the PI or treating investigator
- Any grade 3 non-hematological toxicity lasting for more than 72 hours (with the exception of 1.) alopecia and fatigue and 2.) grade 3 nausea, vomiting, and diarrhea that resolves within 5 days)

DLTs are any of the above that are suspected to be related or possibly related to any one of the drugs in combination. If a patient experiences a DLT, treatment is to be held. Treatment to resume per investigator discretion. Dosing modifications will follow standard of care practices. Dose modifications and treatment delay guidelines for gemcitabine and nab-paclitaxel are described in **Tables 3-5**.

6.3 Toxicities and Dosing Delays/Dose Modifications

Any patient who receives any amount of treatment or the study drugs on this protocol will be evaluable for toxicity. Doses can be delayed or modified in the event of an adverse event or at the physician's discretion. Thus, adverse events will be collected and reviewed at every patient visit. During the DLT assessment period, patients will be evaluated for DLT within 1 week (±7 days) after the last dose of gemcitabine/nab-paclitaxel, if doses are delayed. If there is more than a 2-week delay between doses (all doses must be received within 4 weeks), the patient is not evaluable (but they will continue to follow the protocol-directed procedures).

To appropriately assess toxicity and possible DLTs during treatment, patients will be seen in clinic by a practitioner every week, or per investigator's discretion, with blood work drawn as per the study calendar. Therapy may be administered provided that the patient has no evidence of progressive disease and meets criteria for treatment as defined below.

Toxicity will be evaluated using the NCI Common Terminology Criteria for Adverse Events (CTCAE), Version 5.0. The frequency of toxicities per organ system will be tabulated using descriptive statistics. All patients who receive any amount of the study drug will be evaluable for toxicity.

After the DLT period is complete, dosing modifications, timing, and combination is per investigator discretion.

In the event that L-glutamine is held due to toxicity related to L-glutamine, Gemcitabine and Nab-Paclitaxel may continue as long as patient meets criteria for retreatment. Dose modifications and treatment delay guidelines for gemcitabine and nab-paclitaxel are described in **Tables 3-5**.

6.3.1 Dose Modification and Treatment Delay Guidelines for L-glutamine

L-glutamine will be administered for 3-7 days, per Investigator's discretion, prior to initiation of gemcitabine and paclitaxel. During both the 3-7 day lead-in and dose-finding portion of this study, L-glutamine will be given at predefined doses of 0.1, 0.2, or 0.3 g/kg oral twice daily (rounded to nearest 5 g with an upper limit of 15 g twice daily (30 g/day)

for any dose level). The most common adverse reactions (incidence >10%) from L-glutamine treatment have included constipation, nausea, headache, abdominal pain, cough, pain in extremity, back pain, and chest pain, although these were all AEs reflective of a study population with sickle cell anemia or sickle β^0-thalassemia.[61] Specifically, treatment discontinuation due to adverse reactions was reported in 2.7% (n=5) of patients receiving L-glutamine and included one case each of hypersplenism, abdominal pain, dyspepsia, burning sensation, and hot flash. Serious adverse reactions were reported in both treatment groups, more frequently in the placebo group, and were consistent with underlying diseases of sickle cell anemia or sickle β^0-thalassemia.[61] As such, no dosing modifications and treatment delays have been recommended in the FDA package insert.

We anticipate L-glutamine will be well-tolerated at full FDA-labeled doses in combination with gemcitabine and nab-paclitaxel based on review of 47 prospective trials investigating enteral glutamine in over 1600 adult patients (including those treated with chemotherapy and radiation therapy, **Appendix A**). We may encounter nausea and abdominal discomfort as dose-limiting features of L-glutamine intake given the volumes of food or fluid required for mixing and administration. Subjects who cannot tolerate or comply to a minimum of 3 days within the 3-7 day lead-in phase may be replaced per investigator's discretion. Outside of the DLT period, dosing modifications, timing, and combination is per investigator discretion.

6.3.2 Dose Modification and Treatment Delay Guidelines for Gemcitabine and Nab-paclitaxel

Three doses for gemcitabine at 600, 800, and 1000 mg/m^2 intravenous (IV) over 30 minutes on days 1, 8, and 15 and 3 doses for nab-paclitaxel at 75, 100, and 125 mg/m^2 IV over 30 minutes on days 1, 8, and 15 (every 28-day cycles) will be explored in this study. Dose modifications and treatment delay guidelines for gemcitabine and nab-paclitaxel are described in **Tables 3-5**.

After the DLT is complete, Cycle may be repeated when patient meets the criteria as described below:

- ANC ≥ 1,500 cells/mm^3
- Platelet count ≥ 100,000 cells/mm^3
- Non-hematologic toxicity must have resolved to ≤ Grade 1

At the discretion of the investigator, L-Glutamine may be continued in the event that Gemcitabine and/or Nab-paclitaxel is held due to toxicity.

If L-Glutamine is permanently discontinued, patient will be taken off study and may continue treatment as standard of care at the discretion of the investigator.

If Gemcitabine and Nab-Paclitaxel is permanently discontinued, patient will be taken off study.

Table 3. Dose Level Reductions for Treatment-related Adverse Events from Gemcitabine and Nab-paclitaxel

Dose Level[a]	Nab-paclitaxel (mg/m^2)	Gemcitabine (mg/m^2)
Full dose	125	1000
1st dose reduction	100	800
2nd dose reduction	75	600
If additional dose reduction required	Discontinue	Discontinue

[a]Dose reductions should occur with reference to starting dose level, e.g., if starting at 100 mg/m^2 and 800 mg/m^2 of nab-paclitaxel and gemcitabine, respectively, next dose reduction would be 75 mg/m^2 and 600 mg/m^2 level

Table 4. Dose Modifications for Hematologic Toxicities from Gemcitabine and Nab-paclitaxel

Cycle Day	ANC (cells/mm³)		Platelet Count (cells/mm³)	Gemcitabine/nab-paclitaxel
**Day 1**	<1500	OR	<100,000	Delay dose until recovery
**Day 8**	500 to <1000	OR	50,000 to <75,000	Reduce 1 dose level
	<500	OR	<50,000	Withhold dose
**Day 15: IF Day 8 doses were reduced or given without modification:**				
	500 to <1000	OR	50,000 to <75,000	Reduce 1 dose level from Day 8
	<500	OR	<50,000	Withhold dose
**Day 15: IF Day 8 doses were withheld:**				
	≥1000	OR	≥75,000	Reduce 1 dose level from Day 1
	500 to <1000	OR	50,000 to <75,000	Reduce 2 dose levels from Day 1
	<500	OR	<50,000	Withhold dose

Table 5. Dose Modifications for Other Toxicities from Gemcitabine and Nab-paclitaxel

Treatment-related AE	Nab-paclitaxel	Gemcitabine
Febrile Neutropenia: Grade 3 or 4	Withhold until fever resolves and ANC ≥1500; resume at next lower dose level	
Peripheral Neuropathy: Grade 3 or 4	Withhold until improves to ≤grade 1; resume at next lower dose level	No dose reduction
Cutaneous Toxicity: Grade 2 or 3	Reduce to next lower dose level; discontinue treatment if toxicity persists	
Gastrointestinal Toxicity: Grade 3 mucositis or diarrhea	Withhold until improves to ≤grade 1; resume at next lower dose level	
Other Non-hematologic Toxicity: Grade 3 or 4	Withhold until improves to ≤grade 1; resume at next lower dose level (no dose modifications recommended for alopecia, nausea, or vomiting)	Withhold or reduce dose by 50% until resolved (no dose modifications recommended for alopecia, nausea, or vomiting)

Grade 1-2 hypersensitivity reactions have occurred on days of nab-paclitaxel administration consisting of dyspnea (1%) and flushing, hypotension, chest pain, and arrhythmia (all <1%).[15] Premedication may be needed in patients who have had prior hypersensitivity reactions to nab-paclitaxel. Patients who experience a severe hypersensitivity reaction to nab-paclitaxel should not be re-challenged with this drug, and are discontinued from the study. Permanently discontinue gemcitabine for any of the following: unexplained dyspnea or other evidence of severe pulmonary toxicity, severe hepatic toxicity, hemolytic-uremic syndrome, capillary leak syndrome, or posterior reversible encephalopathy syndrome. Should one drug be permanently discontinued, continuation of other drugs is permitted.

6.4 Concomitant Medications/Treatments

The following concomitant medications are prohibited:

- Any investigational agent
- Systemic corticosteroid therapy exceeding 10 mg/day of prednisone or equivalent (except for pre-medication, treatment of study drug-related AEs, and as anti-emetic agent prior to chemotherapy). Topical, inhaled and intranasal corticosteroid is allowed.
- Other immunosuppressives or immunomodulating agents (e.g. cyclophosphamide, methotrexate, azathioprine, and tumor necrosis factor-α blockers)
- Other chemotherapy or anticancer treatment not stated in the protocol. Any parenteral nutrition, enteral (tube) feeding, or other nutritional supplement

To date, L-glutamine has not been shown to interact with any other concomitant medications. Gemcitabine has little or no effect on the pharmacokinetics (clearance and half-life) of nab-paclitaxel and nab-paclitaxel has little or no effect on the

pharmacokinetics of gemcitabine. Nab-paclitaxel is catalyzed by CYP2C8 and CYP3A4 and caution should be exercised when administering concomitantly with medicines known to inhibit (e.g., ketoconazole and other imidazole antifungals, erythromycin, fluoxetine, gemfibrozil, cimetidine, ritonavir, saquinavir, indinavir, and nelfinavir) or induce (e.g., rifampicin, carbamazepine, phenytoin, efavirenz, and nevirapine) either CYP2C8 or CYP3A4. **Patients on strong CYP2C8 or CYP3A4 inhibitors or inducers should discontinue at least one week prior to starting nab-paclitaxel (Appendix E).**

6.5 Duration of Study Participation

All screening procedures are required within 4 weeks of the 3-7 day L-glutamine lead-in phase, or within 5 weeks of C1D1.

The DLT assessment of this study will be the first cycle over 28 days. Then patients will continue on treatment until PD or until any of the following occur:

- Inter-current illness that prevents further administration of treatment
- Unacceptable adverse event(s)
- Experiencing DLT(s), which in the opinion of the PI precludes resuming treatment with dose reduction due to unfavorable risk-benefit ratio (for the first 28-day cycle)
- Patient decides to withdraw from the study, **OR**
- General or specific changes in the patient's condition render the patient unacceptable for further treatment in the judgment of the investigator.

In the absence of the above criteria, subjects may continue to receive study treatment at the investigator's discretion.

6.5.1 Duration of Follow Up

Patients will undergo an End of Treatment assessment within **30 days** after removal from treatment. Patients removed from treatment for unacceptable adverse events will then be followed until resolution or stabilization of the adverse event and will continue to be followed for the duration of the study as per protocol. After the End of Treatment Assessment, follow-up will involve every 3-month phone calls or medical chart review to collect survival data for 24 months.

6.6 Removal of Patients from Protocol

Patients will be removed from the study when any of the criteria listed in **Section 7.5** apply. If a patient is not compliant with doses during lead-in (Sec 7.5.6), they may be replaced at investigator's discretion. Notify the Principal Investigator and document the reason for study removal and the date the patient was removed on the Case Report Form. The patient should be followed-up per protocol.

6.7 Subject Replacement

Subjects who withdraw from the study treatment prior to starting study intervention (C1D1) will be replaced. Subjects who cannot complete a minimum of 3 days during the 3-7 day lead-in of L-glutamine may be replaced at investigator's discretion.

7.0 STUDY PROCEDURES

7.1 Screening/Baseline Procedures

Assessments performed exclusively to determine eligibility for this study will be done only after obtaining informed consent. Assessments performed for clinical indications (not exclusively to determine study eligibility) may be used for baseline values even if the studies were done before informed consent was obtained.

All screening procedures must be performed **within 4 weeks of 3-7 day L-glutamine lead-in or within 5 weeks of Day 1** unless otherwise stated. The screening procedures include:

7.1.1 Informed Consent *must be obtained within 6 weeks of Day 1*

7.1.2 Medical history and record review

Relevant medical history, including history of current disease, surgical history, and information regarding underlying diseases will be recorded at Screening.

7.1.3 Demographics

Age, gender, race, ethnicity

7.1.4 Review subject eligibility criteria

7.1.5 Concomitant Medications

7.1.6 Physical exam including *vital signs (temperature, pulse, respirations, blood pressure), height, and weight*

7.1.7 Performance status *evaluated prior to study entry according to Eastern cooperative group (ECOG) criteria or Karnofsky Performance Status (KPS) scale* (**Appendix B**)

7.1.8 Adverse event assessment

Baseline adverse events will be assessed from C1D1 until the End of Treatment Visit (within 30 days of treatment discontinuation +//-7 days). See Section 8.0 for Adverse Event monitoring and reporting.

Duration (start and stop dates and times), severity/grade, outcome, treatment and relation to study drug will be recorded on the case report form (CRF).

7.1.9 Laboratory Evaluations

CBC with differential, CMP

7.1.10 Pregnancy Testing for WOCBP

Urine or serum pregnancy required within 14 days prior to receiving first dose of study medication for eligibility. If urine test is positive or cannot be confirmed negative, then a serum pregnancy test is required. For safety, a negative pregnancy test is required within 72 hours of first dose but does not preclude eligibility verification. Note: If screening pregnancy test is done within 72 hours of C1D1, it is not required to be repeated.

7.1.11 Blood draw for tumor assessment

Carbohydrate antigen (CA) 19-9 will be assessed

7.1.12 Blood draw for plasma metabolomics

Obtained along with standard of care laboratory evaluations (under fasted, morning blood collection) for baseline plasma metabolomics. Refer to Section 9.0.

7.1.13 Stool sample for microbiome analyses

Obtained prior to initiation of L-glutamine lead-in and upon completion of glutamine lead-in but prior to initiation of chemotherapy, if available.

7.1.14 CT imaging/scan

Baseline evaluation of measurable disease per RECIST v1.1.

7.2 Procedures During Treatment

These visits can occur within a window of +/- 3 business days of the anticipated visit date to account for clinic closures or holidays.

7.2.1 <u>Cycle 1 (First 4 weeks / 28 days)</u>

Day 1, 8, 15, and 28 (or Day 1 of Cycle 2)

- Safety evaluation (as per CTCAE version 5.0)
- Physical Exam
- Weight
- Vital signs
- ECOG performance status or KPS (**Appendix B**)
- Concomitant medications
- Blood collection (CBC, CMP)
- Blood collection for plasma metabolomics following 1-week of L-glutamine administration (under fasted, morning blood collection)
- Drug diary review (oral drug compliance <85% is considered a deviation that is reportable to the IRB)
- Treatment as per dosing schedule

7.2.2 <u>Cycle 2 Day 1, 8, 15, and Cycle 3 (D1, 8, 15) onward (each cycle is 4 weeks/28 days)</u>

Weekly (Day 1, 8, 15 of every cycle)

- Safety evaluation (as per CTCAE version 5.0)
- Physical Exam
- Weight
- Vital signs
- ECOG performance status or KPS (**Appendix B**)
- Concomitant medications
- Blood collection (CBC, CMP)
- Tumor marker (CA 19-9) every 28 days from Cycle 1 Day 1
- Drug diary review (oral drug compliance <85% is considered a deviation that is reportable to the IRB)
- Treatment as per dosing schedule

7.2.3 Every 2 cycles (every 8 weeks/2 months)

Day 1 (Clinic Visit, +/- 7 days)

- CT imaging/scan: Evaluation of tumor response (Every 8 weeks as per standard of care, +/- 7 days)

7.2.4 End of Treatment (EOT) Visit

Within 30 days after treatment termination (+/- 7 days)

- Safety evaluation (as per CTCAE version 5.0)
- Physical Exam
- Weight
- Vital signs
- ECOG performance status or KPS (**Appendix B**)
- Concomitant medications
- Drug diary review (oral drug compliance <85% is considered a deviation that is reportable to the IRB)
- Blood collection (CBC, CMP)
- Blood collection for plasma metabolomics (under fasted, morning blood collection)
- Tumor marker (CA 19-9)
- Continue treatment as per physician's discretion

7.3 Follow-up Procedures

Patients will be followed every 3 months for 24 months after EOT visit/completion of (or early withdrawal from) study treatment or until death. The following will be assessed during follow-up:

- Medical chart review for survival. If there are no records of patient survival in the medical chart, PI or study staff may call to assess survival.

7.4 Time and Events Table

PROCEDURE	Screening/ Baseline	3-7 Day Lead-in of L-glutamine	Treatment phase until progression, intolerance, or withdrawal		End of Treatment	Follow-up
	All to occur within 4 weeks prior to the lead-in week of L-glutamine or within 5 weeks of Cycle 1 Day 1		Day 1, 8, 15, 28 (+/- 3 days) of **Cycle 1** L-glutamine, gemcitabine, and nab-paclitaxel	Day 8, 15 (+/- 3 days) of **Cycle 2** and Day 1, 8, 15 (+/- 3 days) **Cycle 3 onward**	Within 30 days after study termination (+/- 7 days)	Survival follow-up every 3 months for 24 months (+/- 7 days)
Informed Consent[a]	X					
Eligibility review	X					
Medical history review[b]	X					X
Demographics	X					
Performance Status (ECOG/KPS)[c]	X		X	X	X	
Concomitant medications	X		X	X	X	
Complete Physical Exam	X		X	X	X	
Height	X					
Weight	X		X	X	X	
Vital Signs[d]	X		X	X	X	
Adverse Event Assessment[e]	X		X	X	X	
Blood collection (CBC, CMP)	X		X	X	X	
Pregnancy test[f]	X					
Plasma metabolomics[g]	X		X		X	
Tumor assessment (imaging)[h]	X			X[h] *(every 2 cycles)*		
Tumor marker (CA 19-9)[i]	X			X	X	
Drug diary review[j]			X	X	X	
L-glutamine		X[k]	X	X		
Gemcitabine[l]			X	X		
Nab-paclitaxel[l]			X	X		

Survival Follow-up[m]						X
Stool microbiome[n]	X					

Footnotes:

[a]Informed consent must be obtained within 6 weeks of Cycle 1 Day 1

[b]Relevant medical history, including history of current disease, surgical history, and information regarding underlying diseases will be recorded at Screening.

[c]According to Eastern Cooperative Oncology Group (ECOG) criteria or Karnofsky Performance Status (KPS) scale (**Appendix B**)

[d]Vital signs to include temperature, pulse, respirations, blood pressure

[e]Safety evaluation as per CTCAE version 5.0; **DLT assessment window is first cycle (4 weeks)**

[f]Urine or serum pregnancy test within 14 days prior to receiving the first dose of study medication for eligibility verification. If the urine test is positive or cannot be confirmed as negative, a serum pregnancy test will be required. Note: Negative urine or serum pregnancy test is also conducted within 72 hours prior to C1D1 for study procedures and should not preclude eligibility verification, but if screening pregnancy test is done within 72 hours of C1D1, it is not required to be repeated.

[g]Collected at the same time of the day (morning timeframe of 7-11 a.m.) and in fasting patients (at least 8 hours from last meal) at baseline, after a 1-week administration of L-glutamine, weekly throughout the first cycle of combination gemcitabine/nab-paclitaxel and L-glutamine (including Day 28 of Cycle 1 or Day 1 of Cycle 2), and upon disease progression, intolerance to therapy, or other reason for study discontinuation. We expect at most 7 peripheral blood collections for each subject (including baseline) for metabolomics.

[h]Evaluation of tumor response (every 8 weeks as per standard of care, +/- 7 days) according to RECIST v1.1 (Appendix D)

[i]Every 28 days from Cycle 1 Day 1

[j]All subjects will be provided with instructions and drug diary

[k]L-glutamine will be administered alone for 3-7 days prior to addition of gemcitabine/nab-paclitaxel. L-glutamine will be given as 0.1, 0.2, or 0.3 g/kg oral twice daily (rounded to nearest 5 g) with an upper limit of 15 g twice daily (30 g/day) for any dose level. See Section 6.0 for more details.

[l]Gemcitabine and nab-paclitaxel doses are assigned. See Section 6.0 Treatment Plan. Treatment can continue even after completion of study per investigator's discretion.

[m]Survival follow-up includes medical chart review for survival. If no records of patient survival in medical chart, PI or study staff may call to assess survival.

[n]Collected at baseline or prior to initiation of L-glutamine lead-in and upon completion of glutamine lead-in but prior to initiation of chemotherapy, if available.

7.5 Removal of Subjects from Study

Patients can be taken off the study treatment and/or study at any time at their own request, or they may be withdrawn at the discretion of the investigator for safety, behavioral or administrative reasons. The reason(s) for discontinuation will be documented and may include:

7.5.1 Patient voluntarily withdraws (follow-up permitted);
7.5.2 Patient withdraws consent (termination of treatment and follow-up);
7.5.3 Patient is unable to comply with protocol requirements;
7.5.4 Patient demonstrates disease progression (unless continued treatment with study drug is deemed appropriate at the discretion of the investigator);
7.5.5 Patient experiences toxicity that makes continuation in the protocol unsafe;
7.5.6 Patient is unable to finish a minimum of 3 days of L-glutamine during 3-7 day lead-in;
7.5.7 Treating physician determines continuation on the study would not be in the patient’s best interest;
7.5.8 Patient becomes pregnant (pregnancy to be reported along same timelines as a serious adverse event);
7.5.9 Development of second malignancy (except for basal cell carcinoma or squamous cell carcinoma of the skin) that requires treatment, which would interfere with this study;
7.5.10 Lost to follow-up. If a research subject cannot be located to document survival after a period of 2 years, the subject may be considered “lost to follow-up.” All reasonable efforts must be made to locate subjects to determine and report their ongoing status. Lost to follow-up is defined by the inability to reach the subject after a minimum of three documented phone calls, faxes, or emails as well as lack of response by subject to one registered mail letter. All attempts should be documented in the subject’s medical records. If it is determined that the subject has died, the site will use permissible local methods to obtain the date and cause of death. If after all attempts, the subject remains lost to follow-up, then the last known alive date as determined by the investigator should be reported and documented in the subject’s medical records.

8.0 ADVERSE EVENTS (AE)

8.1 Adverse Event Monitoring

The investigator or designee is responsible for ensuring that all adverse events (both serious and non-serious) observed by the clinical team or reported by the subject which occur after the subject has signed the informed consent are fully recorded in the subject’s medical records for as long as the event is ongoing. Source documentation must be available to support all adverse events.

A laboratory test abnormality considered clinically relevant (e.g., meets the definition of a DLT, causing the subject to withdraw from the study, requiring treatment or causing apparent clinical manifestations, result in a delay or dose modification of study treatment, or judged relevant by the investigator), should be reported as an adverse event.

The investigator, sub-investigator (treating physician if applicable), or research nurses will provide the following for all adverse events (both serious and non-serious):

- Event term (as per CTCAE) - Descriptions and grading scales found in the NCI Common Terminology Criteria for Adverse Events (CTCAE) version 5.0 will be utilized for AE reporting.

- Description of the event
- Date of onset and resolution
- Expectedness of the toxicity
- Grade of toxicity
- Attribution of relatedness to the investigational agent- (this must be assigned by an investigator, sub-investigator, or treating physician)
- Action taken as a result of the event, including but not limited to; no changes, dose interrupted, reduced, discontinued, etc. or action taken with regard to the event, i.e. no action, received conmed or other intervention, etc.
- Outcome of event

All patients experiencing an adverse event, regardless of its relationship to study drug, will be monitored until:

- the adverse event resolves or the symptoms or signs that constitute the adverse event return to baseline
- any abnormal laboratory values have returned to baseline
- there is a satisfactory explanation other than the study drug for the changes observed, or
- death.

8.2 Definitions

8.2.1 Adverse Event

An adverse event is any untoward medical occurrence associated with the use of a drug in humans, whether or not considered drug related.

8.2.2 Serious Adverse Event or Serious Suspected Adverse Reaction

An adverse event or suspected adverse reaction is considered “serious” if, in the view of either the investigator or sponsor, it results in any of the following outcomes:

8.2.2.1 Death.

8.2.2.2 A life-threatening adverse event.

8.2.2.3 Inpatient hospitalization or prolongation of existing hospitalization

8.2.2.4 A persistent or significant incapacity or substantial disruption of the ability to conduct normal life functions

8.2.2.5 A congenital anomaly/birth defect

8.2.2.6 Important medical events that may not result in death, be life-threatening, or require hospitalization may be considered serious

when, based upon appropriate medical judgment, they may jeopardize the patient or subject and may require medical or surgical intervention to prevent one of the outcomes listed in this definition. Examples of such medical events include allergic bronchospasm requiring intensive treatment in an emergency room or at home, blood dyscrasias or convulsions that do not result in inpatient hospitalization, or the development of drug dependency or drug abuse.

8.2.3 Unanticipated Problem (UP)

Unanticipated problems include any incident, experience, or outcome that meets all of the following criteria:

- Unexpected (in terms of nature, severity, frequency) given (a) the research procedures that are described in the protocol-related documents, such as the IRB-approved research protocol and informed consent document; and (b) the characteristics of the subject population being studied;

- Related or possibly related to participation in the research (possibly related means there is a reasonable possibility that the incident, experience, or outcome may have been caused by the procedures involved in the research); and

- Suggests that the research places subjects or others at a greater risk of harm (including physical, psychological, economic, or social harm) than was previously known to an individual or group of individuals (including research subjects, research staff, or others not directly involved in the research).

8.2.4 Evaluable for toxicity

Any patient who receives at least one dose of investigational product is evaluable for toxicity and will be included in the safety analysis.

8.3 Severity of Adverse Events

All non-hematologic adverse events will be graded according to the NCI Common Terminology Criteria for Adverse Events (CTCAE) version 5.0. The CTCAE current version is available at **http://ctep.cancer.gov/reporting/ctc.html**

8.4 Reporting Requirements for Adverse Events

8.4.1 Steps to Determine If an Adverse Event Requires Expedited Reporting

Step 1: Identify the type of adverse event using the NCI Common Terminology Criteria for Adverse Events (CTCAE version 5.0).

Step 2: Grade the adverse event using the NCI CTCAE version 5.0

Step 3: Determine whether the adverse event is related to the protocol therapy

Attribution categories are as follows:

- Definite – The AE *is clearly related* to the study treatment.

- Probable – The AE *is likely related* to the study treatment.
- Possible – The AE *may be related* to the study treatment.
- Unlikely – The AE *is doubtfully related* to the study treatment.
- Unrelated – The AE *is clearly NOT related* to the study treatment.

Note: This includes all events that occur within 30 days (Refer to Section 8.1) of the last dose of protocol treatment. Any event that occurs more than 30 days (Refer to Section 8.1) after the last dose of treatment and is attributed (possibly, probably, or definitely) to the agent(s) must also be reported accordingly.

Step 4: Determine the prior experience of the adverse event.

Expected events are those that have been previously identified as resulting from administration of the agent. An adverse event is considered unexpected, for expedited reporting purposes only, when either the type of event or the severity of the event is not listed in:

- the current known adverse events listed in the Agent Information Section of this protocol;
- the drug package insert; and/or
- the current Investigator's Brochure

8.4.2 Expedited Reporting

8.4.2.1 Reporting to the Principal Investigator

The Principal Investigator must be notified by study staff or co-investigators within 24 hours of learning of any serious adverse events, regardless of attribution, occurring during the study or within 30 days (Refer to Section 8.1) of the last administration of the study drug.

Phone Number for Expedited Reporting:
Jun Gong, MD
Email: Jun.Gong@cshs.org
Phone: (310)-423-5776

Alternate Phone Number for Expedited Reporting:
Andrew Hendifar, MD
Email: Andrew.Hendifar@cshs.org
Phone: (310) 423-2217

8.4.2.2 Reporting to DSMC

Serious Adverse Events deemed to be related to the protocol and on-study deaths, including death of a research subject unless the death is expected (e.g. due to disease progression) to be reported to the DSMC within 24 hours of awareness for medical monitor ad hoc review between meetings to determine if immediate action is required. Reports to be emailed to the DSMC team at GroupSOCCICCTODSMCAdmin@cshs.org.

8.4.2.3 Reporting to the Institutional Review Board (IRB)

The CSMC IRB must be notified within 10 business days of "any unanticipated problems involving risk to subjects or others."

1. Any serious event (injuries, side effects, deaths or other problems), which in the opinion of the Principal Investigator was unanticipated, involved risk to subjects or others, and was possibly related to the research procedures.
2. Any serious accidental or unintentional change to the IRB-approved protocol that alters the level of risk.
3. Any deviation from the protocol taken without prior IRB review to eliminate apparent immediate hazard to a research subject.
4. Any new information (e.g., publication, safety monitoring report, updated sponsor safety report), interim result or other finding that indicates an unexpected change to the risk/benefit ratio for the research.
5. Any breach in confidentiality that may involve risk to the subject or others.
6. Any complaint of a subject that indicates an unanticipated risk or that cannot be resolved by the Principal Investigator.

8.4.2.4 Reporting to the Food and Drug Administration

The investigator or designee must submit SAEs on FDA Form 3500A (MedWatch) according to the following reporting criteria:

- Reporting any unexpected fatal or life-threatening suspected adverse reactions no later than 7 calendar days after initial receipt of the information

- Reporting any (1) serious, unexpected suspected adverse reactions, (2) findings from other clinical, animal, or in-vitro studies that suggest significant human risk, and (3) a clinically important increase in the rate of a serious suspected adverse reaction no later than 15 calendar days after determining that the information qualifies for reporting

8.4.2.5 Reporting to Emmaus

All SAEs to be forwarded to Emmaus in the same time frame as FDA regulatory reporting; within 7 calendar days of knowledge of the event for fatal or life threatening unexpected, suspected serious adverse events, and within 15 calendar days for non-fatal, non-life-threatening unexpected, suspected adverse events. The format can be in either the completed 3500A (MedWatch) or CIOMS form. This includes expedited and non-expedited SAE reports and follow-ups. This will allow Emmaus to cross report to other clinical trials/investigators in a timely manner. Emmaus may request additional or supportive information to clarify details in a follow-up report and will submit request to the site in writing.

9.0 CORRELATIVES/SPECIAL STUDIES

9.1 Collection of Plasma Specimens

All consented and enrolled patients in this study will need to undergo serial peripheral blood draws during predefined study timepoints (Refer to Section 7.0 and Section 7.4) and under prespecified conditions (i.e., fasted, morning blood collections) for evaluation of changes in plasma metabolites before and after study treatment.

Peripheral blood draws (one 10 mL K2 EDTA vacutainer tube per collection) for plasma metabolomics will be collected at the same time of the day (morning timeframe of 7-11 a.m.) and in fasting patients (at least 8 hours from last meal) at baseline, after a 1-week administration of L-glutamine, weekly throughout the first cycle of combination gemcitabine/nab-paclitaxel and L-glutamine (including Day 28 of Cycle 1 or Day 1 of Cycle 2), and upon disease progression, intolerance to therapy, or other reason for study discontinuation (**Figure 2**). We expect at most 7 peripheral blood collections for each subject (including baseline) for metabolomics. All venous blood draws for plasma metabolomics will be collected during standard clinical phlebotomy under prespecified

conditions as described previously according to the following standard operating protocol (SOP):

Figure 2. Schedule of blood collections for plasma metabolomics (Refer to Lab manual).

Research procedure/samples type	Screening/ Baseline	Post 3-7 day Lead-in with L-glutamine	C1D1	C1D8	C1D15	C1D28	End-of-Treatment Visit
Plasma Metabolomics – One (1) x 10 ml K2 EDTA Vacutainer	X	X	X	X	X	X	X

Vacutainers for Processing:

- 1 x 10 mL K2 EDTA vacutainer tube for each blood draw performed by skilled phlebotomist

Blood Processing Schema Per Collection

Vacutainer Type	Fractionation derivative	Aliquot Volume
10 mL K2 EDTA	EDTA plasma-unquenched	0.2 mL

Blood Collection, Processing, and Storage:

a. Refer to Lab Manual for more details.

9.2 Sample Processing/Specimen Banking

Processing K2 EDTA vacutainers for plasma

a. After collection, invert tube twice to ensure adequate mixing of EDTA.

b. Centrifuge the EDTA vacutainer within one hour of collection at 2,000 x g for 15 minutes in a refrigerated centrifuge (4°C). If unable to process samples at 4°C then spinning at room temperature is acceptable if done within 1 hour of draw but must be noted on the Lab Requisition Form.

c. If the interval between specimen collection and processing is not immediate, keep specimen on ice until centrifuging is performed.

d. Aliquot 200 µl plasma into one 2.0 ml cryovial. Place a label on each vial with the following (1) standard de-identified CCTO labeling standards, (2) specimen type (e.g., plasma metabolomics), (3) unique vial number. Refer to Lab Manual.

i. Place cryovials into the cryovial storage box and immediately freeze at -70 to -90° C and store frozen until ready to ship/transfer.

e. Remaining plasma will be aliquoted into as many 2.0 ml cryovials as possible. The remaining aliquots should be 1.0 ml aliquots in each of the 2.0 ml cryovials.

f. Place cryovials into a Mr. Frosty Freezing Container and place it at -70 to -90° C to freeze slowly. Once cryovials are frozen completely they can be transferred to liquid nitrogen (LN2) for long term storage.

g. Record the storage location onto the *Biobank Processing Form* and add the location onto Oncore.

9.3 Shipping/Plasma Metabolomics

For exploratory analyses, plasma samples will be transported to the CSMC Proteomics and Metabolomics Core where plasma metabolomic assays will be performed in conjunction with the Van Eyk laboratory. The plasma metabolomics assay (Agilent Triple Quad) is coupled to a high-pressure liquid chromatography (HPLC) set up and has been specifically purposed for MS-based quantitation whereby measurements of peak area under the curve (AUC) for metabolites of interest are performed. An automated sample preparation method using the Bravo Metabolomics Sample Prep Platform (Bravo) and Agilent Captiva EMR-Lipid plate will be used to precipitate plasma proteins to quench enzymatic activity, depletes lipids, and extracts metabolites ahead of the LC/MS analysis (**Appendix C**).

9.4 Stool Microbiome Analyses

Stool Collection, Processing, and Storage:

a. Refer to Lab Manual for more details.
b. Place labeled stool at -20 to 80° C. They can be transferred to -80ºC for long term storage.
c. Record the storage location onto the Biobank Processing Form and add the location onto Oncore.

Stool microbiome analyses will occur immediately upon enrollment of the last subject with post-L-glutamine lead-in stool collected but will be batched as a single analysis through the UCLA Microbiome Core. Shotgun metagenomics will be performed using the NovaSeq™ 6000 Sequencing System by the UCLA Microbiome Core according to the following workflow:

1. Frozen stool specimens will be delivered to the UCLA Microbiome Core for aliquoting.
2. When ready for shotgun metagenomics, 0.25-0.5 g aliquots of stool are used.
3. Extract bacterial DNA using the ZymoBIOMICS DNA kit (cat#D4300) with bead beating per protocol: https://files.zymoresearch.com/pdf/d4300t_d4300_d4304_zymobiomics_dna_miniprep_kit_1-3-0.pdf.
4. Library generation and shotgun metagenomic sequencing via the NovaSeq™ 6000 Sequencing System per protocol: https://support.illumina.com/content/dam/illumina-support/documents/documentation/system_documentation/novaseq/novaseq-6000-system-guide-1000000019358-09.pdf.
5. Process raw data using R platforms and quality-filtered reads into operational taxonomic units matched to publicly available reference genomes such as the Greengenes or Silva database, performed by the UCLA Microbiome Core: http://www.microbiome.ucla.edu/cores/microbiome-core/.

6. Perform post-processing analysis and review results with the UCLA Microbiome Core and CSMC Biostatistics and Bioinformatics Core.

10.0 STATISTICAL CONSIDERATIONS

10.1 Sample Size and Randomization

This is a single-institution, open-label, nonrandomized, single-arm, prospective phase I trial investigating the feasibility of combination L-glutamine, gemcitabine, and nab-paclitaxel in treatment-naïve, unresectable or metastatic pancreatic cancer. We propose to accrue 16 patients with advanced or unresectable pancreatic cancer to this dose-finding study exploring 3 doses for gemcitabine, 600, 800, and 1000 mg/m^2 IV over 30 minutes on days 1, 8, and 15, 3 doses of nab-paclitaxel, 75, 100, and 125 mg/m^2 IV over 30 minutes on days 1, 8, and 15, and 3 doses of L-glutamine powder, 0.1, 0.2, and 0.3 g/kg oral twice daily (rounded to nearest 5 g with an upper limit of 15 g twice daily (30 g/day) for any dose level), with L-glutamine starting 1 week (+/- 1 day) prior to initiation of gemcitabine and nab-paclitaxel. Our sample size will not be based on power of a study, instead we will enroll a total of n=16 patients into the DLT assessment cohort whereby the RP2D will be assessed by EWOC, an adaptive Bayesian design as described in Section 6.0 using the dosing schema proposed in **Table 2**.

Operating Characteristics. For the purpose of deriving the operating characteristics of the study, we will assume that successive cohorts of 1 patient will be treated and the dose level for the first patient will be dose level 0. The dose for each subsequent patient will be determined so that, based on all available data, the probability that it exceeds the MTD is equal to a prespecified value α. In this trial, we start at α=0.4 and increase α in small increments of 0.05 until α=0.5, this value being a compromise between the therapeutic aspect of the agent and its toxic side effects. No dose skipping will be permitted. **The trial will be terminated if the posterior probability that the probability that DLT at dose level -2 exceeds θ=0.8 or more.**

A maximum of 16 patients will be accrued to the trial. Upon completion of the trial, the RP2D will be estimated as the median of the marginal posterior distribution of the MTD rounded to the nearest discrete dose level. The computation of the dose to be administered to each patient and the estimate of the MTD will be carried out by Dr. Tighiouart using R and JAGS.

We evaluated the design operating characteristics of this algorithm under 3 scenarios for the true value of the MTD. **Table 6** shows various summary statistics such as the percent of MTD recommendation using 1000 trial replicates, average % of DLTs across all trials, % of trials with an excessive rate of DLT, and % of patients allocated to each dose level. For each scenario, the true MTD is indicated in bold (probability of DLT=0.33). All 3 scenarios are misspecified so that the results of the simulations do not depend on the logistic model. In scenario 1, the MTD is dose level -2. In this case, the probability of recommending this dose level is 0.816 and 58% of the patients are allocated to that level. The average percent of DLT is 42.5%, about 10% higher than the target which is expected when the minimum dose level is the MTD. In scenario 2, the MTD is dose level 0. In this case, the percent of recommendation is 57.3% and the trial is safe. In scenario 3, the MTD is the last dose level. In this case, the percent MTD recommendation is 61.3% and 93.7% of the trial will recommend either dose level 1 or 2 as the true MTD. Most patients are allocated to the MTD and the trial is very safe. Based on these scenarios, we conclude that the design performs well with a sample size of 16 patients.

**Table 6. Design Operating Characteristics for Safety and Efficiency (true MTD shown in bold)**					
	Dose Level				
**Scenario 1**	**-2**	-1	0	1	2

True Prob DLT	**0.33**	0.45	0.65	0.75	0.90
%MTD recommendation	**81.6**	17.7	7.0	0.0	0.0
% patients allocated	57.9	26.1	12.1	3.3	0.6
Ave % DLTs	42.5				
% Trials: rate DLT > θ+0.1	57.5				
	Dose Level				
**Scenario 2**	-2	-1	**0**	1	2
True Prob DLT	0.01	0.05	**0.33**	0.50	0.70
%MTD recommendation	0.0	28.0	**57.3**	13.9	0.8
% patients allocated	2.5	25.5	46.1	18.4	7.2
Ave % DLTs	30.1				
% Trials: rate DLT > θ+0.1	5.1				
	Dose Level				
**Scenario 3**	-2	-1	0	1	**2**
True Prob DLT	0.001	0.03	0.08	0.15	**0.33**
%MTD recommendation	0.00	0.20	0.61	32.4	**61.3**
% patients allocated	0.50	2.73	14.95	27.37	54.42
Ave % DLTs	23.08				
% Trials: rate DLT > θ+0.1	0.50				

On review of our annual SOCCI gastrointestinal oncology (GI) Oncology Program census, the PI and co-investigator, Dr. Andrew Hendifar, see at minimum 4 new PDAC cases per month where the majority (approximately 60%) are unresectable or metastatic at presentation in our high-volume GI oncology clinic at CSMC. Therefore, we expect to be able to accrue 16 patients with advanced pancreatic cancer over 12 months for DLT assessment and longitudinal collection of blood samples, accounting for screen failures. Survival and exploratory analyses would occur over the subsequent 12 months. We estimate the total study duration to be 24 months.

10.2 Data Sets Analyzed

All eligible patients who are enrolled into the study and receive at least one dose of the study drug (the Safety Population) will be included in the safety and preliminary efficacy analysis. All enrolled subjects will undergo peripheral blood draws for plasma metabolomics and if available, stool collection for microbiome analyses. Those having received study drug will be included in the exploratory biomarker analyses.

10.3 Data Analyses/Study Endpoints

10.3.1 Primary Objective Analysis

The primary analysis will be based on all enrolled subjects receiving at least one dose of study drug. The primary analysis of RP2D will be assessed by the number of DLTs, defined as the rate of drug-related grade ≥3 adverse events (AEs) experienced within the first 4 weeks (cycle 1) of study treatment. The RP2D is defined as the dose level closest to the median of the posterior distribution of the MTD. The MTD is defined as the dose level such that the probability of DLT at the MTD is θ=0.33.

10.3.2 Secondary Objective Analysis

Secondary objectives include safety and preliminary efficacy. Safety will be assessed by AEs as summarized in terms of type and severity by NCI CTCAE version 5.0. All subjects entered into the study who receives any amount of study

drug will be included in the safety analysis. The frequencies of AEs by type, body system, severity and relationship to study drug will be summarized. SAEs (if any) will be described in detail.

Clinical activity of the combination will be assessed by the objective response rate (ORR), progression-free survival (PFS), and overall survival (OS) evaluated every 8 weeks (every 2 cycles ±1 week) according to the revised RECIST guidelines (version 1.1, **Appendix D**). Since the goal is to characterize the preliminary efficacy of the combination of L-glutamine, gemcitabine, and nab-paclitaxel, no formal hypothesis testing will take place and will not be based on power of a test.

10.3.3 Correlative Studies

Exploratory endpoints for the correlative biomarker analyses include measurement of peak AUCs from a 219 central carbon metabolite panel as assessed by MS-based quantitation of plasma samples. Given that the primary objective of the correlative studies is to determine the feasibility of longitudinally collecting peripheral blood samples across predefined timepoints for plasma metabolomics in our study cohort, no formal hypothesis testing will take place and will not be based on power of a test.

Plasma metabolites collected at baseline (i.e., prior to initiation of the 3-7 day lead-in of L-glutamine) will be correlated to a comprehensive list of clinicopathologic variables including site of metastases, carbohydrate antigen (CA) 19-9 level, demographics, cachexia, pathologic features, PFS, and OS. In longitudinally collected blood samples from this same cohort, we will measure changes in metabolite levels at the following: 1.) after a 1-week administration of L-glutamine, 2.) weekly throughout the first cycle of combination gemcitabine/nab-paclitaxel and L-glutamine, and 3.) upon disease progression, intolerance to therapy, or other reason for study discontinuation.

Bacterial abundance from baseline (prior to L-glutamine lead-in) and post-L-glutamine lead-in stools of enrolled PDAC patients with advanced PDAC will be associated with clinicopathologic variables including cachexia and rates of gastrointestinal side effects.

Univariate analyses will be used to assess the association of metabolites with clinicopathologic variables using parametric or nonparametric tests such as Pearson or Spearman correlation coefficients, two-sample t-test or Mann-Whitney test. Preliminary analyses will be carried out to compare changes in metabolites from baseline to each of the time points using paired t-tests or Wilcoxon sign-test controlling for the proportion of false positives among all significant results with a false discovery rate (FDR) of 0.1 via the Benjamini and Hochberg procedure to adjust for multiplicity.[68]

10.4 Data Analysis Plans

Exploratory Data Analyses

Exploratory analyses will be performed to elucidate the relationships among variables. Graphical data analysis will be applied to examine the distribution of the data and to reveal the association among covariates and outcome variables. Statistical graphics provide effective data summaries and can be very useful for error checking and outlier identification. Standard distribution plots such as histograms and box-plots will be applied. A scatter plot matrix will be used to explore the association among multiple variables. These analyses not only

provide an assumption free, graphical, presentation of the data, but can also be used to help select appropriate statistical models to use during data analysis.

Analysis of Categorical Response Variables

We will test for differences among groups using Pearson's chi-square statistic, or Fisher's exact test.

Analysis of Survival Data

We will estimate the survival distributions for time-to-event outcomes using the method of Kaplan and Meier and compare these distributions among groups using the logrank test.

Analysis of Continuous Response Variables

For the analysis of continuous response variables, we will use analysis of variance (ANOVA), analysis of covariance (ANCOVA), and mixed models. We will test the data to ensure that the underlying assumptions (i.e., normality, homoscedasticity) of the ANOVA model are met. If these assumptions are not met, then we will attempt to transform the data such that the transformed data meet these assumptions. Standard transformations of the response variable such as the log, square root and Box-Cox transformations will be examined. If data transformation is inadequate to meet the analysis assumptions, then rank transformation of the data will be performed and one-way ANOVA on the rank transformed response variables will be analyzed and reported.

11.0 STUDY MANAGEMENT

11.1 Conflict of Interest

Any reportable conflict of interest will be disclosed to the local IRB and will be outlined in the Informed Consent Form.

11.2 Institutional Review Board (IRB) Approval and Consent

It is expected that the IRB will have the proper representation and function in accordance with federally mandated regulations. The IRB should approve the consent form and protocol.

In obtaining and documenting informed consent, the investigator should comply with the applicable regulatory requirement(s), and should adhere to Good Clinical Practice (GCP) and to ethical principles that have their origin in the Declaration of Helsinki.

Before recruitment and enrollment onto this study, the patient will be given a full explanation of the study and will be given the opportunity to review the consent form. Each consent form must include all the relevant elements currently required by the FDA Regulations and local or state regulations. Once this essential information has been provided to the patient and the investigator is assured that the patient understands the implications of participating in the study, the patient will be asked to give consent to participate in the study by signing an IRB-approved consent form.

Prior to a patient's participation in the trial, the written informed consent form should be signed and personally dated by the patient and by the person who conducted the informed consent discussion.

11.3 Registration Procedures

All subjects that sign informed consent will be assigned a subject number sequentially by their date of consent. Those subjects that do not pass the screening phase will be listed as screen failures on the master list of consented subjects. Eligible subjects, as determined by screening procedures and verified by a treating investigator, will be registered on study at Cedars-Sinai Medical Center by the Study Coordinator.

Issues that would cause treatment delays after registration should be discussed with the Principal Investigator (PI). If a patient does not receive protocol therapy following registration, the patient's registration on the study may be cancelled. The Study Coordinator should be notified of cancellations as soon as possible.

Assignment of Subject ID: The study team will track all subjects who sign consent using OnCore. Subjects found to be ineligible will be recorded as screen failures. Subjects found to be eligible will be registered using a three-digit numeric ID that follows the standard SOCCI format (001, 002, etc.).

A) Eligibility Verification

Prior to registration, all subjects must undergo eligibility verification by the SOCCI Cancer Clinical Trials Office (CCTO) Quality Management Core (QMC). The following documents will be organized into an eligibility packet, scanned as a pdf, and emailed to GroupSOCCICROQMC@cshs.org for review:

- Copy of applicable source documents
- QMC-approved eligibility checklist (signed by investigator and 2 members of the study team)
- Signed patient consent form with Subject's Bill of Rights, HIPAA authorization form, consent progress notes and any optional consent forms, as applicable

B) Registration

After eligibility is verified, registration is completed as follows:

- Assign a patient study number
- Assign the patient a dose as determined through communication with Biostatistics and the principal investigator
- Enter the patient in OnCore as on study
- Notify the investigational pharmacy and treating physicians that a subject has gone on study and anticipated treatment start date

Oversight by the principal investigator is required throughout the entire registration process

11.4 Data Management and Quality Control and Reporting

The data will be entered into a HIPAA-compliant database. The Study Staff will be responsible for data processing, in accordance with procedural documentation. Database lock will occur once quality assurance procedures have been completed.

All procedures for the handling and analysis of data will be conducted using good computing practices meeting FDA guidelines for the handling and analysis of data for clinical trials.

11.5 Data and Safety Monitoring

11.5.1 Data Monitoring and Quality Assurance

Moderate Risk Monitoring

Adherence to the protocol, Good Clinical Practices (GCP), and institutional policy will be monitored by the PI during the course of the study through routine Disease Research Group (DRG) meetings or equivalent. In addition, the SOCCI Cancer Clinical Trials Office (CCTO) Quality Management Core (QMC) will conduct internal monitoring visits and audits for data quality and protocol adherence. QMC reports will be forwarded to the SOCCI Data and Safety Monitoring (DSMC). Refer to the DSMC Charter for more details. For any protocol, QMC has the authority to request more frequent reviews or focused safety monitoring if it is deemed appropriate for any reason.

QMC will also conduct the following:

1. Central eligibility verification for all subjects enrolled as described in protocol section 11.3
2. Central review by the SOCCI CCTO Medical Director or designee of all eligibility exception requests and waiver requests to assess appropriateness, to ensure quality data and subject safety protections for investigator-initiated research.

11.5.2 Safety Monitoring

Moderate Risk Monitoring

Oversight of the progress and safety of the study will be provided by the PI. The PI will maintain continuous safety monitoring for the duration of the study by reviewing subject/study data. Adverse events and unanticipated problems are not expected, but if they occur, they will be documented and reported according to CS-IRB policies and procedures. If the PI becomes aware of any new safety information that may place subjects at increased risk than what was previously known, the IRB will be promptly notified and if warranted, enrollment may be held until the PI determines whether a modification to the study is necessary and/or the informed consent documents are updated accordingly. It is the responsibility of the principal investigator to adhere to the Data Safety Monitoring Plan throughout the life of the study.

In addition, this protocol will utilize the SOCCI Data and Safety Monitoring Committee will provide another layer of data and safety oversight. DSMC membership and responsibilities are governed by the committee charter. Every six (6) months the DSMC findings and recommendations will be reported in writing to the Principal Investigator as a summary letter which will be forwarded by the Principal Investigator or designee to the CS-IRB. The DSMC may increase or decrease the frequency of study review, at their discretion. Refer to the DSMC Charter for details of the DSMC review.

11.6 Record Retention

Study documentation includes all Case Report Forms, data correction forms or queries, source documents, monitoring/auditing logs/letters, and regulatory documents (e.g., protocol and amendments, IRB correspondence and approval, signed patient consent forms).

Source documents include all recordings of observations or notations of clinical activities and all reports and records necessary for the evaluation and reconstruction of the clinical research study. Government agency regulations and directives require that the study investigator must retain all study documentation pertaining to the conduct of a clinical

trial. Study documents should be kept on file per local guidelines.

11.7 Adherence to Protocol

Except for an emergency situation in which proper care for the protection, safety, and well-being of the study patient requires alternative treatment, or a protocol exception request approved by the SOCCI Medical Director and CSMC IRB, the study shall be conducted exactly as described in the approved protocol.

11.7.1 Emergency Modifications

Investigators may implement a deviation from, or a change of, the protocol to eliminate an immediate hazard(s) to trial subjects without prior IRB approval. For any such emergency modification implemented, the IRB must be notified as soon as possible, but no more than 10 days from the investigator's awareness of the event.

11.7.2 Protocol Exceptions and Eligibility Waivers

Moderate Risk

A protocol exception is an anticipated or planned deviation from the IRB-approved research protocol, as described in the IRB Policy, *Reporting Possible Unanticipated Problems Involving Risks to Subject or Others (UPIRSO) Policy: Institutional Review Board/Research Compliance and Quality Improvement.* A protocol exception most often involves a single subject and is not a permanent revision to the research protocol. Protocol exceptions that extend beyond a single subject should result in a protocol amendment to avoid serial violations.

Planned exceptions to the protocol that are more than logistical in nature and/or impact an eligibility criterion, affect timing of study drug administration, or the investigator assesses the event may impact subject safety and/or study integrity, may not be implemented without prior approval from the SOCCI (CCTO) Medical Director and the IRB. The PI or her/his designee is responsible for submitting a protocol exception and its supporting documents to the SOCCI CCTO Medical Director for review and further instructions on IRB reporting.

Study team should refer to the IRB *Reporting Possible Unanticipated Problems Involving Risks to Subject or Others (UPIRSO) Policy: Institutional Review Board/Research Compliance and Quality Improvement* guidelines to determine which deviations and exception requests meet reporting guidelines. Once approved by the medical director, the deviation or exception request must be submitted to the IRB for review and approval prior to implementation.

Special considerations for Eligibility Waivers (EW)

In general, subjects who do not meet the eligibility requirements should not be enrolled. In the rare event that it is appropriate for subject inclusion, the rationale/justification and subject case history should be forwarded to the SOCCI CCTO Medical Director for assessment prior to submission to the IRB for approval.

The CCTO Medical Director will review the case and contact the investigator if additional information is needed or further discussion is warranted. The CCTO Medical Director will provide a written assessment/recommended course of action. The CCTO Medical Director's assessment must be uploaded into CS-IRB with the waiver request for IRB review and consideration. The CCTO Medical Director may recommend future protocol changes.

Eligibility Waiver and Exception Request Submission Process

The PI and/or treating physician will provide a written request for an eligibility waiver including case history and justification for prospective deviation from the study design, to the SOCCI CCTO Medical Director. The "IIT Monitoring – Eligibility Waivers and Exception Requests (EW/ER) Form" must be completed then submitted, along with any applicable supporting documents, by email to QMC (GroupSOCCICROQMC@cshs.org) to request an eligibility exception or waiver request from the CCTO Medical Director. This is only a requirement for studies with a DSM category of moderate or high. An assessment from the CCTO Medical Director or designee must be done prior to submission to the IRB for review.

11.7.3 Other Protocol Deviations

Logistical deviations from the protocol (e.g., minor changes to the study schedule for an individual subject) do not require prior IRB approval unless the deviation has the potential to affect the subject's safety or study integrity. Such planned deviations that do meet this definition and do not affect the subject's safety or study integrity should be noted in the subject's research record or deviation log as described in the SOCCI CCTO's Standard Operating Procedure 12: Deviation and Noncompliance Reporting.

Unintentional deviations from the protocol that might affect subject safety or study integrity should be reported to the IRB within 10 days from when the investigator becomes aware that such a deviation has occurred, as outlined in the SOCCI CCTO's Standard Operating Procedure 12: *Deviation and Noncompliance Reporting*. In this case, a Protocol Deviation report must be submitted in CS-IRB, per IRB policy, *Reporting Possible Unanticipated Problems Involving Risks to Subject or Others (UPIRSO) Policy: Institutional Review Board/Research Compliance and Quality Improvement*. All submissions should include a description of the plan to avoid similar deviations or exceptions in the future.

11.7.4 Amendments to the Protocol

Should amendments to the protocol be required, the amendments will be originated and documented by the Principal Investigator. It should also be noted that when an amendment to the protocol substantially alters the study design or the potential risk to the patient, a revised consent form might be required.

The written amendment, and if required the amended consent form, must be sent to the IRB for approval prior to implementation. Repeat exceptions or deviations to the protocol may suggest a protocol amendment is needed.

11.8 Obligations of Investigators

The Principal Investigator is responsible for the conduct of the clinical trial at the site in accordance with Title 21 of the Code of Federal Regulations and/or the Declaration of Helsinki. The Principal Investigator is responsible for personally overseeing the treatment of all study patients. The Principal Investigator must assure that all study site personnel, including sub-investigators and other study staff members, adhere to the study protocol and all FDA/GCP/NCI regulations and guidelines regarding clinical trials both during and after study completion.

The Principal Investigator will be responsible for assuring that all the required data will be collected and entered onto the Case Report Forms and/or into a HIPAA-compliant study database. Periodically, monitoring visits will be conducted and the Principal Investigator will provide access to his/her original records to permit verification of proper entry of data.

At the completion of the study, all case report forms will be reviewed by the Principal Investigator and will require his/her final signature to verify the accuracy of the data.

12.0 REFERENCES

1. Gong J, Tuli R, Shinde A, Hendifar AE. Meta-analyses of treatment standards for pancreatic cancer. Mol Clin Oncol. 2016;4(3):315-25.
2. Aprile G, Negri FV, Giuliani F, De Carlo E, Melisi D, Simionato F, et al. Second-line chemotherapy for advanced pancreatic cancer: Which is the best option? Crit Rev Oncol Hematol. 2017;115:1-12.
3. Bray F, Ferlay J, Soerjomataram I, Siegel RL, Torre LA, Jemal A. Global cancer statistics 2018: GLOBOCAN estimates of incidence and mortality worldwide for 36 cancers in 185 countries. CA: a cancer journal for clinicians. 2018;68(6):394-424.
4. Bryant KL, Mancias JD, Kimmelman AC, Der CJ. KRAS: feeding pancreatic cancer proliferation. Trends Biochem Sci. 2014;39(2):91-100.
5. Son J, Lyssiotis CA, Ying H, Wang X, Hua S, Ligorio M, et al. Glutamine supports pancreatic cancer growth through a KRAS-regulated metabolic pathway. Nature. 2013;496(7443):101-5.
6. Biancur DE, Paulo JA, Malachowska B, Quiles Del Rey M, Sousa CM, Wang X, et al. Compensatory metabolic networks in pancreatic cancers upon perturbation of glutamine metabolism. Nature communications. 2017;8:15965.
7. Ishak Gabra MB, Yang Y, Lowman XH, Reid MA, Tran TQ, Kong M. IKKbeta activates p53 to promote cancer cell adaptation to glutamine deprivation. Oncogenesis. 2018;7(11):93.
8. Lowman XH, Hanse EA, Yang Y, Ishak Gabra MB, Tran TQ, Li H, et al. p53 Promotes Cancer Cell Adaptation to Glutamine Deprivation by Upregulating Slc7a3 to Increase Arginine Uptake. Cell Rep. 2019;26(11):3051-60.e4.
9. Yang Y, Ishak Gabra MB, Hanse EA, Lowman XH, Tran TQ, Li H, et al. MiR-135 suppresses glycolysis and promotes pancreatic cancer cell adaptation to metabolic stress by targeting phosphofructokinase-1. Nat Commun. 2019;10(1):809.
10. Pan M, Reid MA, Lowman XH, Kulkarni RP, Tran TQ, Liu X, et al. Regional glutamine deficiency in tumours promotes dedifferentiation through inhibition of histone demethylation. Nat Cell Biol. 2016;18(10):1090-101.
11. Chen MJ, Wang TE, Tsai SJ, Lin CC, Liu CY, Wang HY, et al. Oral glutamine supplement inhibits ascites formation in peritoneal carcinomatosis mouse model. Gastroenterol Res Pract. 2013;2013:814054.
12. Burris HA, 3rd, Moore MJ, Andersen J, Green MR, Rothenberg ML, Modiano MR, et al. Improvements in survival and clinical benefit with gemcitabine as first-line therapy for patients with advanced pancreas cancer: a randomized trial. J Clin Oncol. 1997;15(6):2403-13.
13. Moore MJ, Goldstein D, Hamm J, Figer A, Hecht JR, Gallinger S, et al. Erlotinib plus gemcitabine compared with gemcitabine alone in patients with advanced pancreatic cancer: a phase III trial of the National Cancer Institute of Canada Clinical Trials Group. J Clin Oncol. 2007;25(15):1960-6.
14. Von Hoff DD, Ervin T, Arena FP, Chiorean EG, Infante J, Moore M, et al. Increased survival in pancreatic cancer with nab-paclitaxel plus gemcitabine. The New England journal of medicine. 2013;369(18):1691-703.
15. Celgene. U.S. Full Prescribing Information for ABRAXANE. 2018:https://www.accessdata.fda.gov/drugsatfda_docs/label/2018/021660s045lbl.pdf. Accessed Oct 20, 2019.
16. Algara M, Rodriguez N, Vinals P, Lacruz M, Foro P, Reig A, et al. Prevention of radiochemotherapy-induced esophagitis with glutamine: results of a pilot study. Int J Radiat Oncol Biol Phys. 2007;69(2):342-9.
17. Anderson PM, Schroeder G, Skubitz KM. Oral glutamine reduces the duration and severity of stomatitis after cytotoxic cancer chemotherapy. Cancer. 1998;83(7):1433-9.
18. Azman M, Mohd Yunus MR, Sulaiman S, Syed Omar SN. Enteral glutamine supplementation in surgical patients with head and neck malignancy: A randomized controlled trial. Head Neck. 2015;37(12):1799-807.
19. Bozzetti F, Biganzoli L, Gavazzi C, Cappuzzo F, Carnaghi C, Buzzoni R, et al. Glutamine supplementation in cancer patients receiving chemotherapy: a double-blind randomized study. Nutrition. 1997;13(7-8):748-51.

20. Canovas G, Leon-Sanz M, Gomez P, Valero MA, Gomis P, La Huerta JJ. Oral glutamine supplements in autologous hematopoietic transplant: impact on gastrointestinal toxicity and plasma protein levels. Haematologica. 2000;85(11):1229-30.
21. Chang SC, Lai YC, Hung JC, Chang CY. Oral glutamine supplements reduce concurrent chemoradiotherapy-induced esophagitis in patients with advanced non-small cell lung cancer. Medicine (Baltimore). 2019;98(8):e14463.
22. Chattopadhyay S, Saha A, Azam M, Mukherjee A, Sur PK. Role of oral glutamine in alleviation and prevention of radiation-induced oral mucositis: A prospective randomized study. South Asian J Cancer. 2014;3(1):8-12.
23. Choi K, Lee SS, Oh SJ, Lim SY, Lim SY, Jeon WK, et al. The effect of oral glutamine on 5-fluorouracil/leucovorin-induced mucositis/stomatitis assessed by intestinal permeability test. Clin Nutr. 2007;26(1):57-62.
24. Daniele B, Perrone F, Gallo C, Pignata S, De Martino S, De Vivo R, et al. Oral glutamine in the prevention of fluorouracil induced intestinal toxicity: a double blind, placebo controlled, randomised trial. Gut. 2001;48(1):28-33.
25. Das S, Kar Mahapatra S, Gautam N, Das A, Roy S. Oxidative stress in lymphocytes, neutrophils, and serum of oral cavity cancer patients: modulatory array of L-glutamine. Support Care Cancer. 2007;15(12):1399-405.
26. de Urbina JJO, San-Miguel B, Vidal-Casariego A, Crespo I, Sanchez DI, Mauriz JL, et al. Effects Of Oral Glutamine on Inflammatory and Autophagy Responses in Cancer Patients Treated With Abdominal Radiotherapy: A Pilot Randomized Trial. Int J Med Sci. 2017;14(11):1065-71.
27. Farias JW, Furtado FS, Guimaraes SB, Silva Filho AR, Vasconcelos PR. Oxidative stress parameters in women with breast cancer undergoing neoadjuvant chemotherapy and treated with nutraceutical doses of oral glutamine. Acta Cir Bras. 2011;26 Suppl 1:82-7.
28. Gul K, Muge A, Taner A, Sehri E. Oral glutamine supplementation reduces radiotherapy- induced esophagitis in lung cancer patients. Asian Pac J Cancer Prev. 2015;16(1):53-8.
29. Huang CJ, Huang MY, Fang PT, Chen F, Wang YT, Chen CH, et al. Randomized double-blind, placebo-controlled trial evaluating oral glutamine on radiation-induced oral mucositis and dermatitis in head and neck cancer patients. Am J Clin Nutr. 2019;109(3):606-14.
30. Huang EY, Leung SW, Wang CJ, Chen HC, Sun LM, Fang FM, et al. Oral glutamine to alleviate radiation-induced oral mucositis: a pilot randomized trial. Int J Radiat Oncol Biol Phys. 2000;46(3):535-9.
31. Jacobson SD, Loprinzi CL, Sloan JA, Wilke JL, Novotny PJ, Okuno SH, et al. Glutamine does not prevent paclitaxel-associated myalgias and arthralgias. J Support Oncol. 2003;1(4):274-8.
32. Jebb SA, Osborne RJ, Maughan TS, Mohideen N, Mack P, Mort D, et al. 5-fluorouracil and folinic acid-induced mucositis: no effect of oral glutamine supplementation. Br J Cancer. 1994;70(4):732-5.
33. Kozelsky TF, Meyers GE, Sloan JA, Shanahan TG, Dick SJ, Moore RL, et al. Phase III double-blind study of glutamine versus placebo for the prevention of acute diarrhea in patients receiving pelvic radiation therapy. J Clin Oncol. 2003;21(9):1669-74.
34. Kucuktulu E, Guner A, Kahraman I, Topbas M, Kucuktulu U. The protective effects of glutamine on radiation-induced diarrhea. Support Care Cancer. 2013;21(4):1071-5.
35. Lopez-Vaquero D, Gutierrez-Bayard L, Rodriguez-Ruiz JA, Saldana-Valderas M, Infante-Cossio P. Double-blind randomized study of oral glutamine on the management of radio/chemotherapy-induced mucositis and dermatitis in head and neck cancer. Mol Clin Oncol. 2017;6(6):931-6.
36. Mochiki E, Ohno T, Yanai M, Toyomasu Y, Andoh H, Kuwano H. Effects of glutamine on gastrointestinal motor activity in patients following gastric surgery. World J Surg. 2011;35(4):805-10.
37. Muscaritoli M, Micozzi A, Conversano L, Martino P, Petti MC, Cartoni C, et al. Oral glutamine in the prevention of chemotherapy-induced gastrointestinal toxicity. Eur J Cancer. 1997;33(2):319-20.
38. Okuno SH, Woodhouse CO, Loprinzi CL, Sloan JA, LaVasseur BI, Clemens-Schutjer D, et al. Phase III controlled evaluation of glutamine for decreasing stomatitis in patients receiving fluorouracil (5-FU)-based chemotherapy. Am J Clin Oncol. 1999;22(3):258-61.
39. Pachon Ibanez J, Pereira Cunill JL, Osorio Gomez GF, Irles Rocamora JA, Serrano Aguayo P, Quintana Angel B, et al. Prevention of oral mucositis secondary to antineoplastic treatments in head and neck cancer by supplementation with oral glutamine. Nutr Hosp. 2018;35(2):428-33.
40. Pathak S, Soni TP, Sharma LM, Patni N, Gupta AK. A Randomized Controlled Trial to Evaluate the Role and Efficacy of Oral Glutamine in the Treatment of Chemo-radiotherapy-induced Oral Mucositis and Dysphagia in Patients with Oropharynx and Larynx Carcinoma. Cureus. 2019;11(6):e4855.
41. Pattanayak L, Panda N, Dash MK, Mohanty S, Samantaray S. Management of Chemoradiation-Induced Mucositis in Head and Neck Cancers With Oral Glutamine. J Glob Oncol. 2016;2(4):200-6.

42. Peterson DE, Jones JB, Petit RG, 2nd. Randomized, placebo-controlled trial of Saforis for prevention and treatment of oral mucositis in breast cancer patients receiving anthracycline-based chemotherapy. Cancer. 2007;109(2):322-31.
43. Rotovnik Kozjek N, Kompan L, Soeters P, Oblak I, Mlakar Mastnak D, Mozina B, et al. Oral glutamine supplementation during preoperative radiochemotherapy in patients with rectal cancer: a randomised double blinded, placebo controlled pilot study. Clin Nutr. 2011;30(5):567-70.
44. Rubio I, Suva LJ, Todorova V, Bhattacharyya S, Kaufmann Y, Maners A, et al. Oral glutamine reduces radiation morbidity in breast conservation surgery. JPEN J Parenter Enteral Nutr. 2013;37(5):623-30.
45. Strasser F, Demmer R, Bohme C, Schmitz SF, Thuerlimann B, Cerny T, et al. Prevention of docetaxel- or paclitaxel-associated taste alterations in cancer patients with oral glutamine: a randomized, placebo-controlled, double-blind study. Oncologist. 2008;13(3):337-46.
46. Stubblefield MD, Vahdat LT, Balmaceda CM, Troxel AB, Hesdorffer CS, Gooch CL. Glutamine as a neuroprotective agent in high-dose paclitaxel-induced peripheral neuropathy: a clinical and electrophysiologic study. Clin Oncol (R Coll Radiol). 2005;17(4):271-6.
47. Tanaka Y, Takahashi T, Yamaguchi K, Osada S, Shimokawa T, Yoshida K. Elemental diet plus glutamine for the prevention of mucositis in esophageal cancer patients receiving chemotherapy: a feasibility study. Support Care Cancer. 2016;24(2):933-41.
48. Topkan E, Parlak C, Topuk S, Pehlivan B. Influence of oral glutamine supplementation on survival outcomes of patients treated with concurrent chemoradiotherapy for locally advanced non-small cell lung cancer. BMC Cancer. 2012;12:502.
49. Topkan E, Yavuz MN, Onal C, Yavuz AA. Prevention of acute radiation-induced esophagitis with glutamine in non-small cell lung cancer patients treated with radiotherapy: evaluation of clinical and dosimetric parameters. Lung Cancer. 2009;63(3):393-9.
50. Tsujimoto T, Yamamoto Y, Wasa M, Takenaka Y, Nakahara S, Takagi T, et al. L-glutamine decreases the severity of mucositis induced by chemoradiotherapy in patients with locally advanced head and neck cancer: a double-blind, randomized, placebo-controlled trial. Oncol Rep. 2015;33(1):33-9.
51. Vahdat L, Papadopoulos K, Lange D, Leuin S, Kaufman E, Donovan D, et al. Reduction of paclitaxel-induced peripheral neuropathy with glutamine. Clin Cancer Res. 2001;7(5):1192-7.
52. Vidal-Casariego A, Calleja-Fernandez A, Cano-Rodriguez I, Cordido F, Ballesteros-Pomar MD. Effects of oral glutamine during abdominal radiotherapy on chronic radiation enteritis: a randomized controlled trial. Nutrition. 2015;31(1):200-4.
53. Wang WS, Lin JK, Lin TC, Chen WS, Jiang JK, Wang HS, et al. Oral glutamine is effective for preventing oxaliplatin-induced neuropathy in colorectal cancer patients. Oncologist. 2007;12(3):312-9.
54. Yoshida S, Matsui M, Shirouzu Y, Fujita H, Yamana H, Shirouzu K. Effects of glutamine supplements and radiochemotherapy on systemic immune and gut barrier function in patients with advanced esophageal cancer. Ann Surg. 1998;227(4):485-91.
55. Jebb SA, Marcus R, Elia M. A pilot study of oral glutamine supplementation in patients receiving bone marrow transplants. Clin Nutr. 1995;14(3):162-5.
56. Schloerb PR, Skikne BS. Oral and parenteral glutamine in bone marrow transplantation: a randomized, double-blind study. JPEN J Parenter Enteral Nutr. 1999;23(3):117-22.
57. Skubitz KM, Anderson PM. Oral glutamine to prevent chemotherapy induced stomatitis: a pilot study. J Lab Clin Med. 1996;127(2):223-8.
58. Ziegler TR, Benfell K, Smith RJ, Young LS, Brown E, Ferrari-Baliviera E, et al. Safety and metabolic effects of L-glutamine administration in humans. JPEN J Parenter Enteral Nutr. 1990;14(4 Suppl):137s-46s.
59. Holecek M. Side effects of long-term glutamine supplementation. JPEN J Parenter Enteral Nutr. 2013;37(5):607-16.
60. Niihara Y, Miller ST, Kanter J, Lanzkron S, Smith WR, Hsu LL, et al. A Phase 3 Trial of l-Glutamine in Sickle Cell Disease. N Engl J Med. 2018;379(3):226-35.
61. Emmaus. U.S. Full Prescribing Information for ENDARI. 2017:https://www.accessdata.fda.gov/drugsatfda_docs/label/2017/208587s000lbl.pdf. Accessed Oct 20, 2019.
62. Niihara Y, Macan H, Eckman JR, Koh H, Cooper ML, Ziegler TR, et al. L-Glutamine Therapy Reduces Hospitalization for Sickle Cell Anemia and Sickle β°-Thalassemia Patients at Six Months - A Phase II Randomized Trial. Clin Pharmacol Biopharm. 2014;3:116.

63. Niihara Y, Matsui NM, Shen YM, Akiyama DA, Johnson CS, Sunga MA, et al. L-glutamine therapy reduces endothelial adhesion of sickle red blood cells to human umbilical vein endothelial cells. BMC Blood Disord. 2005;5:4.
64. Niihara Y, Zerez CR, Akiyama DS, Tanaka KR. Oral L-glutamine therapy for sickle cell anemia: I. Subjective clinical improvement and favorable change in red cell NAD redox potential. Am J Hematol. 1998;58(2):117-21.
65. Ying H, Kimmelman AC, Lyssiotis CA, Hua S, Chu GC, Fletcher-Sananikone E, et al. Oncogenic Kras maintains pancreatic tumors through regulation of anabolic glucose metabolism. Cell. 2012;149(3):656-70.
66. Kim MH, Kim H. The Roles of Glutamine in the Intestine and Its Implication in Intestinal Diseases. Int J Mol Sci. 2017;18(5).
67. Tighiouart M, Cook-Wiens G, Rogatko A. A Bayesian adaptive design for cancer phase I trials using a flexible range of doses. J Biopharm Stat. 2018;28(3):562-74.
68. Yoav B, Hochberg Y. Controlling the False Discovery Rate: A Practical and Powerful Approach to Multiple Testing. . Journal of the Royal Statistical Society Series B. 1995;57(1):289-300.

13.0 APPENDICES

13.1 APPENDIX A: PROSPECTIVE CLINICAL TRIALS INVESTIGATING ORAL GLUTAMINE

Chemotherapy (Y/N)	Glutamine (GLN) Dose and Duration	Reference
N (n=6)	0, 0.1, or 0.3 g/kg oral weekly for 3 doses; whole blood GLN levels peaked 30-45 min (mean 1028 µM 0.1 g/kg and 1328 µM 0.3 g/kg) after ingestion, then to normal 90-120 minutes (0.1 g/kg) or 180-240 min (0.3 g/kg)	Ziegler, 1990
Y (n=17)	4 g oral QID for 8 days; serum GLN mean 0.53 µM (time 0), peak serum GLN seen in 1 patient of 1.33 µM at 15 min, basal GLN levels seen by 60 min	Jebb, 1994
Y (n=24)	16 g oral divided over 4 doses for 1 day	Jebb, 1995
Y (n=14)	4 g oral BID for 28 days or 4 days after resolution of post-chemotherapy mucositis	Skubitz, 1996
Y (n=33)	10 g oral TID for 8 days	Bozetti, 1997
Y (n=11)	6 g oral TID for median 31 days	Muscaritoli, 1997
Y (n=7)	30 g oral daily for 28 days; mean plasma GLN was 496.5 µmole/mL (pretreatment), 494.2 µmole/mL (day 7 and 14), 466.4 µmole/mL (day 28)	Yoshida, 1997
Y (n=24)	2 g/m^2 oral BID on days of chemotherapy and for ≥14 additional days	Anderson, 1998
N (n=7)	10 g oral TID for 4 weeks; whole blood NADH (nmol/mL RBC) 47.5 (baseline) vs. 72.1 (week 4), redox potential (%) 47.2 (baseline) vs. 62.1 (week 4)	Niihara, 1998
Y (n=66)	4 g oral BID for 14 days	Okuno, 1999
Y (n=66)	10 g oral TID, variable duration	Schloerb, 1999
Y (n=not reported)	20 g oral daily until neutrophil count >500 cells/mm^3 or TPN started	Canovas, 2000
N (n=8)	4 g oral QID for 25 days	Huang, 2000
Y (n=29)	6 g oral TID for 15 days	Daniele, 2001
Y (n=12)	10 g oral TID for 4 days	Vahdat, 2001
Y (n=36)	10 g oral TID for 5 days	Jacobson, 2003
Y (n=64)	4 g oral BID 7 days per week on RT then 2 weeks after RT	Kozelsky, 2003
N (n=10)	30 g oral daily for 4-8 weeks	Niihara, 2005
Y (n=17)	10 g oral TID for 4 days	Stubblefield, 2005
Y (n=75)	10 g oral TID until 15 days after RT	Algara, 2007
Y (n=22)	10 g oral TID for 15 days	Choi, 2007
N (n=16)	30 g oral daily for 14 days after surgery	Das, 2007
Y (n=163)	7.5 g oral daily for 14 days after last dose of chemotherapy, until mucositis resolved, or until end of treatment cycle; most common adverse events (AEs): nausea (8.8%), dry mouth (5.2%), vomiting (1.6%)	Peterson, 2007
Y (n=42)	15 g oral BID for 7 days every 2 weeks	Wang, 2007
Y (n=21)	30 g oral daily for at least 2 months; most common AEs grade ≥2: anorexia (38.1%), nausea (33.3%), neuropathy (23.8%)	Strasser, 2008
Y (n=22)	10 g oral TID for 2 weeks after RT; median weight change of 0.8 kg (-1.8-5.3)	Topkan, 2009
Y (n=20)	15 g oral daily during chemotherapy	Farias, 2011
Y (n=15)	3 g oral daily until 2 days after surgery	Mochiki, 2011
Y (n=33)	10 g oral TID for 5 weeks	Rotovnik Kozjek, 2011
Y (n=56)	10 g oral TID until 2 weeks after RT; grade 1-2 nausea (32.1%) and vomiting (19.6%)	Topkan, 2012
Y (n=23)	5 g oral TID until 1 week after RT	Kucuktulu, 2013
N (n=9)	0.5 g/kg oral daily for 7 weeks; whole blood GLN ~600 µmole/L (time 0), ~750 µmole/L (week 7)	Rubio, 2013
N (n=35)	10 g oral daily on days of RT	Chattopadhyay, 2014
N (n=33)	0.3 g/kg oral BID for 48 weeks, then 3-week taper; 8.1% adverse events (AEs) possibly L-GLN-related: sickle cell crisis most common (83.8%)	Niihara, 2014
N (n=22)	10 g oral TID for median 8 days	Azman, 2015
Y (n=16)	10 g oral TID until 2 weeks after RT	Gul, 2015
Y (n=20)	10 g oral TID during chemoRT	Tsujimoto, 2015
N (n=34)	30 g oral daily during RT	Vidal-Casariego, 2015
Y (n=81)	15 g oral BID during chemoRT; most common AEs (all-grade): pain (98.7%), dysphagia (82.6%), cough (40.7%)	Pattanayak, 2016
Y (n=10)	8.9 g oral daily for 2 cycles of chemotherapy; only grade ≥3 AE was neutropenia (30%)	Tanaka, 2016
Y (n=25)	10 g oral TID during chemoRT	Lopez-Vaquero, 2017
N (n=22)	10 g oral TID until the end of RT	de Urbina, 2017
Y (n=131)	10 g oral TID throughout treatment	Pachon Ibanez, 2018
N (n=152)	0.3 g/kg oral BID for 48 weeks, then 3-week taper; most common all-grade AEs: constipation (25.2%), nausea (22.5%), headache (21.2%)	Niihara, 2018
Y (n=30)	10 g oral TID during chemoRT	Chang, 2019
Y (n=36)	10 g oral TID until 2 weeks after RT	Huang, 2019
Y (n=30)	10 g oral daily 5 days/week during chemoRT	Pathak, 2019

13.2 APPENDIX B: EASTERN COOPERATIVE ONCOLOGY GROUP (ECOG) AND KARNOFSKY PERFORMANCE STATUS (KPS) SCALES

ECOG Performance Status Scale

ECOG PERFORMANCE STATUS SCALE GRADE	DESCRIPTION
0	Fully active, able to carry on all pre-disease activities without restriction.
1	Restricted in physically strenuous activities and able to carry out work of a light or sedentary nature, e.g. light housework, office work.
2	Ambulatory and capable of all self-care but unable to carry out any work activities. Up and about more than 50% of waking hours.
3	Capable of only limited self-care; confined to bed or chair more than 50% of waking hours.
4	Completely disabled. Cannot carry on any self-care. Totally confined to bed or chair.
5	Dead.

Oken, M.M., Creech, R.H., Tormey, D.C., Horton, J., Davis, T.E., McFadden, E.T., Carbone, P.P.: Toxicity And Response Criteria Of The Eastern Cooperative Oncology Group. Am J Clin Oncol 5:649-655, 1982.

Karnofsky Performance Status Scale

KARNOFSKY PERFORMANCE STATUS SCALE

Able to carry on normal activity and to work; no special care needed.	100	Normal no complaints; no evidence of disease.
	90	Able to carry on normal activity; minor signs or symptoms of disease.
	80	Normal activity with effort; some signs or symptoms of disease.
Unable to work; able to live at home and care for most personal needs; varying amount of assistance needed.	70	Cares for self; unable to carry on normal activity or to do active work.
	60	Requires occasional assistance, but is able to care for most of his personal needs.
	50	Requires considerable assistance and frequent medical care.
Unable to care for self; requires equivalent of institutional or hospital care; disease may be progressing rapidly.	40	Disabled; requires special care and assistance.
	30	Severely disabled; hospital admission is indicated although death not imminent.
	20	Very sick; hospital admission necessary; active supportive treatment necessary.
	10	Moribund; fatal processes progressing rapidly.
	0	Dead

Schag CC, Heinrich RL, Ganz PA. Karnofsky performance status revisited: reliability, validity, and guidelines. J Clin Oncol. 1984 Mar;2(3):187-93.

13.3 APPENDIX C: PLASMA METABOLOMICS LC/MS PROTOCOL

1. Retrieve frozen plasma samples from LN2 and transport to the CSMC Proteomics and Metabolomics Core.
2. Thaw and transfer at least 100 µL of plasma into a screw top tube and place into a Thermo Scientific matrix tube rack.
3. Load tube rack onto the Bravo deck for automated precipitation of plasma proteins and quenching of post-sampling enzymatic activity via the addition of 450 µL of methanol:ethanol (50:50) per 100 µL aliquot of plasma.
4. Transfer quenched plasma sample onto the Agilent Captiva EMR-Lipid plate for automated protein removal and lipid depletion and collect resulting metabolites in filtrate.
5. Prior to dry-down, resuspension, and injection of the eluate into the Agilent Triple Quad MS-HPLC system, turn on the MS-HPLC system and start the Data Acquisition program.
    a. In Windows, click Start > Agilent > MassHunter Workstation > Data Acquisition
6. In the Data Acquisition main window, configure set up acquisition methods, run samples interactively or automatically, monitor instrument status, monitor runs, and tune the instrument for LC/MS analysis.
7. Prepare the LC Module by switching LC stream to Waste and Purge the LC pump by following the directions for purging the pump in the User Guide.
8. Set up to condition or equilibrate the column.
    a. Type LC parameters and click the Apply button in the toolbar to download them to the LC.
    b. Right-click an LC module in the Instrument Status window and click one of the commands to change any non-method control parameters, if necessary.
    c. Monitor the baseline and adjust the plot to make sure the column is equilibrated and the baseline stable.
9. Set up to view real-time parameter values (actuals). As you prepare for a run and during a run, you want to see the actual values of the instrument parameters. You can do this in the Instrument Status window.
    a. Right-click the Actuals list to see the Setup command.
    b. Click Setup to bring up the list of Actuals available for monitoring.
    c. Add all the parameter values you intend to monitor and click the OK button.
10. Set up real-time plot displays to monitor the effluent.
    a. Right-click the chromatogram plot and click Change.
11. Prepare the Triple Quadrupole instrument.
    a. From the Context list, click Tune.
    b. Click Checktune to evaluate if the MS parameters are within the limits to produce the specified mass accuracy and resolution.
    c. If Checktune fails, then you can try using the Manual Tune tab to fix the problem.
    d. If Checktune fails again, you must run an Autotune.
    e. In the Context list, select Acquisition.
12. After conditioning the column and tuning the Triple Quadrupole MS, switch the LC stream from Waste to MS.
13. Set up and run an acquisition method according to the User Guide.
14. Set up and run interactive samples by clicking the Sample Run window.
15. Review results and analyze data with the Quantitative Analysis program.

13.4 APPENDIX D: RESPONSE EVALUATION CRITERIA IN SOLID TUMORS (RECIST VERSION 1.1)

Eligibility

Only patients with measurable disease at baseline should be included in protocols where objective tumor response is the primary endpoint.

- **Measurable disease** - the presence of at least one measurable lesion. If the measurable disease is restricted to a solitary lesion, its neoplastic nature should be confirmed by cytology/histology.
- **Measurable lesions** - lesions that can be accurately measured in at least one dimension (longest diameter in the plane of measurement is to be recorded) with a minimum size of:
  - 10 mm by CT scan (CT scan slice thickness no greater than 5 mm)
  - 10 mm caliper measurement by clinical exam (lesions which cannot be accurately measured with calipers should be recorded as non-measurable)
  - 20 mm by chest X-ray.

Malignant lymph nodes: to be considered pathologically enlarged and measureable, a lymph node must be ≥ 15 mm in short axis when assessed by CT scan (CT scan slice thickness recommended to be no greater than 5 mm). At Baseline and follow-up, only the short axis will be measured and followed.

- **Non-measurable lesions** - all other lesions, including small lesions (longest diameter <10 mm or pathological lymph nodes with ≥ 10 to <15 mm short axis) as well as truly non-measurable lesions. Lesions considered truly non-measurable include: leptomeningeal disease, ascites, pleural/pericardial effusion, inflammatory breast disease, lymphangitis cutis/pulmonis, abdominal masses/abdominal organomegaly identified by physical exam that is not measurable by reproducible imaging techniques.
  - For special considerations regarding lesion measurability for bone lesions, cystic lesions and lesions with prior local treatment, consult the RECIST 1.1 guidelines in the Study Reference Manual.
  - All measurements should be taken and recorded in metric notation using a ruler or calipers. All baseline evaluations should be performed as closely as possible to the beginning of treatment and never more than 4 weeks before beginning of treatment.
  - The same method of assessment and the same technique should be used to characterize each identified and reported lesion at baseline and during follow-up.
  - Clinical lesions will only be considered measurable when they are superficial (e.g., skin nodules and palpable lymph nodes). For the case of skin lesions, either a CT scan or documentation by color photography, including a ruler to estimate the size of the lesion, is to be done

Methods of Measurement

- CT and MRI are the best currently available and reproducible methods to measure target lesions selected for response assessment. Conventional CT and MRI should be performed with cuts of 10 mm or less in slice thickness contiguously. Spiral CT should be performed using a 5 mm contiguous reconstruction algorithm. This applies to tumors of the chest, abdomen and pelvis. Head and neck tumors and those of extremities usually require specific protocols.
- Lesions on chest X-ray are acceptable as measurable lesions when they are clearly defined and surrounded by aerated lung. However, CT is preferable.
- When the primary endpoint of the study is objective response evaluation, ultrasound (US) should not be used to measure tumor lesions. It is, however, a possible alternative to clinical measurements of superficial palpable lymph nodes, subcutaneous lesions and thyroid nodules. US might also be useful to confirm the complete disappearance of superficial lesions usually assessed by clinical examination.
- The utilization of endoscopy and laparoscopy for objective tumor evaluation has not yet been fully and widely validated. Their uses in this specific context require sophisticated equipment and a high level of expertise that may only be available in some centers. Therefore, the utilization of such techniques for objective tumor response should be restricted to validation purposes in specialized centers. However, such techniques can be useful in confirming complete pathological response when biopsies are obtained.
- Tumor markers alone cannot be used to assess response. If markers are initially above the upper normal limit, they must normalize for a patient to be considered in complete clinical response when all lesions have disappeared.
- Cytology and histology can be used to differentiate between PR and CR in rare cases (e.g., after treatment to differentiate between residual benign lesions and residual malignant lesions in tumor types such as germ cell tumors).

Baseline documentation of “Target” and “Non-Target” lesions

- **Target Lesions** - all measurable lesions up to a maximum of 2 lesions per organ and 5 lesions in total, representative of all involved organs should be identified as ***target lesions*** and recorded and measured at baseline.
  - Target lesions should be selected on the basis of their size (lesions with the longest diameter) and their suitability for accurate repeated measurements (either by imaging techniques or clinically).
  - A sum of the longest diameter (LD) for *all target lesions* will be calculated and reported as the baseline sum LD. The baseline sum LD will be used as reference by which to characterize the objective tumor.
- **Non-target lesions** - all other lesions (or sites of disease) should be identified as ***non-target lesions*** and should also be recorded at baseline. Measurements of these lesions are not required, but the presence or absence of each should be noted throughout follow-up.

Response Criteria Evaluation of Target Lesions

**Complete Response (CR):**	Disappearance of all target lesions. Any pathological lymph nodes (whether target or non-target) must have reduction in short axis to <10 mm).
**Partial Response (PR):**	At least a 30% decrease in the sum of the LD of target lesions, taking as reference the baseline sum LD
**Progressive Disease (PD):**	At least a 20% increase in the sum of the LD of target lesions from the smallest sum of the LD recorded since the treatment started; the sum must also demonstrate an absolute increase of at least 5 mm. The appearance of ≥1 new lesion is also considered progression.
**Stable Disease (SD):**	Neither sufficient shrinkage to qualify for PR nor sufficient increase to qualify for PD, taking as reference the smallest sum LD since the treatment started

Evaluation of non-target lesions

**Complete Response (CR):**	Disappearance of all non-target lesions and normalization of tumor marker level. All lymph nodes must be non- pathological in size (<10 mm short axis)
**Incomplete Response/ Stable Disease (SD):**	Persistence of one or more non-target lesion(s) or/and maintenance of tumor marker level above the normal limits
**Progressive Disease (PD):**	Appearance of one or more new lesions and/or unequivocal progression of existing non-target lesions*

* Although a clear progression of “non target” lesions only is exceptional, in such circumstances, the opinion of the treating physician should prevail and the progression status should be confirmed later on by the review panel (or study chair).

Evaluation of best overall response

- The best overall response is the best response recorded from the start of the treatment until disease progression/recurrence (taking as reference for PD the smallest measurements recorded since the treatment started). In general, the patient's best response assignment will depend on the achievement of both measurement and confirmation criteria

Target Lesions	Non-Target Lesions	New Lesions	Overall Response
CR	CR	No	CR
CR	Incomplete response/SD	No	PR

PR	Non-PD	No	PR
SD	Non-PD	No	SD
PD	Any	Yes or No	PD
Any	PD	Yes or No	PD
Any	Any	Yes	PD

- Patients with a global deterioration of health status requiring discontinuation of treatment without objective evidence of disease progression at that time should be classified as having "symptomatic deterioration". Every effort should be made to document the objective progression even after discontinuation of treatment.
- In some circumstances it may be difficult to distinguish residual disease from normal tissue. When the evaluation of complete response depends on this determination, it is recommended that the residual lesion be investigated (fine needle aspirate/biopsy) to confirm the complete response status.

Confirmation

- The main goal of confirmation of objective response is to avoid overestimating the response rate observed. In cases where confirmation of response is not feasible, it should be made clear when reporting the outcome of such studies that the responses are not confirmed.
- To be assigned a status of PR or CR, changes in tumor measurements must be confirmed by repeat assessments that should be performed no less than 4 weeks after the criteria for response are first met. Longer intervals as determined by the study protocol may also be appropriate.
- In the case of SD, follow-up measurements must have met the SD criteria at least once after study entry at a minimum interval (in general, not less than 6-8 weeks) that is defined in the study protocol.

Duration of overall response

- The duration of overall response is measured from the time measurement criteria are met for CR or PR (whichever status is recorded first) until the first date that recurrence or PD is objectively documented, taking as reference for PD the smallest measurements recorded since the treatment started.

Duration of stable disease

- SD is measured from the start of the treatment until the criteria for disease progression are met, taking as reference the smallest measurements recorded since the treatment started.
- The clinical relevance of the duration of SD varies for different tumor types and grades. Therefore, it is highly recommended that the protocol specify the minimal time interval

required between two measurements for determination of SD. This time interval should take into account the expected clinical benefit that such a status may bring to the population under study.

Response review

- For trials where the response rate is the primary endpoint it is strongly recommended that all responses be reviewed by (an) expert(s) independent of the study at the study's completion. Simultaneous review of the patients' files and radiological images is the best approach.

Reporting of results

- All patients included in the study must be assessed for response to treatment, even if there are major protocol treatment deviations or if they are ineligible. Each patient will be assigned one of the following categories: 1) complete response, 2) partial response, 3) stable disease, 4) progressive disease, 5) early death from malignant disease, 6) early death from toxicity, 7) early death because of other cause, or 8) unknown (not assessable, insufficient data).
- All of the patients who met the eligibility criteria should be included in the main analysis of the response rate. Patients in response categories 4-8 should be considered as failing to respond to treatment (disease progression). Thus, an incorrect treatment schedule or drug administration does not result in exclusion from the analysis of the response rate. Precise definitions for categories 4-8 will be protocol specific.

All conclusions should be based on all eligible patients.

- Subanalyses may then be performed on the basis of a subset of patients, excluding those for whom major protocol deviations have been identified (e.g., early death due to other reasons, early discontinuation of treatment, major protocol violations, etc.). However, these subanalyses may not serve as the basis for drawing conclusions concerning treatment efficacy, and the reasons for excluding patients from the analysis should be clearly reported.
- The 95% confidence intervals should be provided.

Definition of CT tumor response by RECIST 1.1 criteria:

The following table outlines the response categories by RECIST 1.1 criteria.

Target Lesions	Non-Target Lesions	New Lesions	Overall Response
Complete Response (sum of diameters=0 mm)	Complete response	No	Complete Response
Complete Response	Non-complete response, non-progressive disease	No	

Complete Response	Not evaluated	No	Partial Response
Partial Response (decrease in sum of target lesions by ≥30%)	Non-progressive disease OR Not evaluated	No	
Stable Disease	Non-progressive disease OR Not evaluated	No	Stable Disease
Not all evaluated	Non-progressive disease	No	Not Evaluable
Progressive Disease (increase in sum of target lesions by ≥ 20% with an absolute increase in summed diameters by 5mm)	Any	Yes or No	Progressive Disease
Any	Progressive Disease	Yes or No	
Any	Any	Yes	

(Eisenhauer et al. 2009)

13.5 APPENDIX E: CYP3A4 and CYP2C8 INHIBITORS/INDUCERS

CYP3A4 inhibitors and inducers include but are not limited to the following. There could also be additional new drugs and marketed drugs that could be identified as inhibitors/inducers with continued research.

**Strong Inducers**
Carbamazepine
Enzalutamide<br>Mitotane<br>Phenytoin
Rifampin
St. John's Wort

Note: Any additional strong CYP3A4 inducers that are identified or become commercially available while the clinical trial is ongoing are also prohibited.

FDA. "Drug Development and Drug Interactions: Table of Substrates, Inhibitors and Inducers" http://www.fda.gov/Drugs/DevelopmentApprovalProcess/DevelopmentResources/DrugInteractionsLabeling/ucm093664.htm#potency

CYP2C8 inhibitors and inducers include but are not limited to the following. There could also be additional new drugs and marketed drugs that could be identified as inhibitors/inducers with continued research.

Drug
**Strong Inhibitors**
Clopidogrel
Gemfibrozil
**Moderate Inducer**
Rifampin

Note: Any additional strong CYP2C8 inhibitors/inducers that are identified or become commercially available while the clinical trial is ongoing are also prohibited.

FDA. "Drug Development and Drug Interactions: Table of Substrates, Inhibitors and Inducers"http://www.fda.gov/Drugs/DevelopmentApprovalProcess/DevelopmentResources/DrugInteractionsLabeling/ucm093664.htm#potency

13.6 APPENDIX F: SUMMARY OF CHANGES

Amendment 1 (Protocol Version 2 dated 12MAY2021)

- Cover Page – protocol version update; addition of affiliate site THO
- Protocol Signature Page – protocol version update
- Table of Contents – updated
- Minor reformatting throughout
- Study Summary – addition of affiliate site THO
- Section 5.0: Patient Eligibility
    - Inclusion 5.1.1 revised with minor clarification to recurrent tumor not required to be histologically confirmed, and correction of typo from >12 months to ≥12 months (from Protocol Clarification Memo #1).
    - Inclusion 5.1.6 incorporates Protocol Clarification Memo #1 to clarify that pregnancy testing used for eligibility verification is required within 14 days prior to the start of study treatment with note that typically a 72 hour negative pregnancy test is confirmed prior to start of treatment for safety. If the 72 hour pregnancy test is used for screening, then it does not need to be repeated.
    - Exclusion 5.2.2 incorporates Protocol Clarification Memo #1 to clarify that prior chemotherapy treatment in the metastatic setting is allowed but must have been completed at least 12 months prior to recurrence.
    - Exclusion 5.2.11 adds clarity for definition of "other nutritional supplement" as any other nutritional supplement containing *any* amount of glutamine.
- Section 7.0: Study Procedures
    - Section 7.1.10 Pregnancy Testing for WOCBP revised to align with Inclusion 5.1.6 where screening pregnancy testing is required within 14 days prior to the start of study treatment for eligibility verification, and within 72 hours prior to treatment start for safety. If 72 hour window pregnancy testing is used for screening, it does not need to be repeated.
- Section 7.4: Time and Events Table
    - Footnote *f* revised to align with pregnancy testing requirements and study window as detailed in Inclusion 5.1.6 and Section 7.1.10

Amendment 2 (Protocol version 3 dated 09SEP2021)

- Cover Page: Removed Veronica Placencio-Hickok, PhD as a Co-I.
- Study Schema: Changed the length of the lead-in phase from 1 week to 3-7 days, per Investigator's discretion.
- Study Summary: Updated exploratory objective to include analysis of the stool microbiome from before and after L-glutamine supplementation
- Section 1.4: Added reference and rationale for stool microbiome analysis during L-glutamine lead-in
- Section 2.3.2: Added exploratory objective of stool microbiome analysis
- Section 3.3.2: Added exploratory endpoint of relative bacterial abundance as assessed by shotgun metagenomics from stool samples collected from enrolled subjects at baseline and after L-glutamine lead-in prior to initiation gemcitabine/nab-paclitaxel
- Section 4.0: Added to study design that we will evaluate changes in stool microbiome before and after L-glutamine to investigate the impact of specific gut bacteria on promoting gut health.
- List of Abbreviations & Section 5.1.4, Table 1, Inclusion criterion: Added PoCT creatinine.
- Section 6.1, 6.1.1, 6.3.1, 6.5, 7.1, 10.3.3: Changed the length of the lead-in phase from 1 week to 3-7 days, per Investigator's discretion.
- Section 6.1.1., 7.2.1, 7.2.2 & 7.2.4: Indicated that during the treatment phase only, oral compliance of <85% will be considered a protocol deviation which is reportable to the IRB.

- Section 6.2, Definition of Dose-Limiting Toxicity: Indicated that dose modifications and treatment delay guidelines for gemcitabine and nab-paclitaxel are described in Tables 3-5.
- Section 6.3, Toxicities and Dosing Delays/Dose Modifications: In the event that L-glutamine is held due to toxicity related to L-glutamine, Gemcitabine and Nab-Paclitaxel may continue as long as patient meets criteria for retreatment.
- Section 6.3.2, Dose Modification and Treatment Delay Guidelines for Gemcitabine and Nab-paclitaxel: Added additional dose modifications and treatment delay guidelines for DTLs:
    - After the DLT is complete, Cycle may be repeated when patient meets the criteria as described below:
        - ANC ≥ 1,500 cells/mm3
        - Platelet count ≥ 100,000 cells/mm3
        - Non-hematologic toxicity must have resolved to ≤ Grade 1
    - At the discretion of the investigator, L-Glutamine may be continued in the event that Gemcitabine and/or Nab-paclitaxel is held due to toxicity.
    - If L-Glutamine is permanently discontinued, patient will be taken off study and may continue treatment as standard of care at the discretion of the investigator.
    - If Gemcitabine and Nab-Paclitaxel is permanently discontinued, patient will be taken off study.
- Section 6.6 & 7.5.6, Removal of Patients from Protocol: Specified that patients who are not compliant with doses during the 3-7 day lead-in phase may be removed.
- Section 6.7, Replacement of Patients: Removed 75% compliance threshold. Revised to indicate that patients who do not complete a minimum of 3 days during the 3-7 day lead-in phase may be replaced, per Investigator discretion.
- Section 7.1.13: Added stool collections as part of baseline procedures (prior to initiation of L-glutamine lead-in and upon completion of glutamine lead-in but prior to initiation of chemotherapy)
- Section 7.4: Added stool collections to study event table
- Section 7.4: Time and Events table: Revised to indicate that the lead-in phase is 3-7 days.
- Section 8.2, Adverse Events: Updated definition of adverse event so it is consistent with 21 CFR § 312.32.
- Section 9.2, Correlatives/Special Studies: Updated Figure 2 (Schedule of blood collections for plasma metabolomics) so it is consistent with the Lab Manual.
- Section 9.4: Added stool collections, processing, and storage as well as SOP for shotgun metagenomics
- Section 10.2: Included stool microbiome in Data Sets Analyzed section
- Section 10.3.3: Included analyses of stool microbiome in correlative studies of Data Analysis/Endpoints section
- Section 6.1 & 7.4: Clarified that there will be a 3-7 day lead-in period of L-glutamine to align with other sections.

Statistical Analysis Plan

This is a single-institution, open-label, nonrandomized, single-arm, prospective phase I trial investigating the feasibility of combination L-glutamine, gemcitabine, and nab-paclitaxel in treatment-naïve, unresectable or metastatic pancreatic cancer. We propose to accrue 16 patients with advanced or unresectable pancreatic cancer to this dose-finding study exploring 3 doses for gemcitabine, 600, 800, and 1000 mg/m^2 IV over 30 minutes on days 1, 8, and 15, 3 doses of nab-paclitaxel, 75, 100, and 125 mg/m^2 IV over 30 minutes on days 1, 8, and 15, and 3 doses of L-glutamine powder, 0.1, 0.2, and 0.3 g/kg oral twice daily (rounded to nearest 5 g with an upper limit of 15 g twice daily (30 g/day) for any dose level), with L-glutamine starting 1 week (+/- 1 day) prior to initiation of gemcitabine and nab-paclitaxel. Our sample size will not be based on power of a study, instead we will enroll a total of n=16 patients into the dose-limiting toxicity (DLT) assessment cohort whereby the recommended phase II dose (RP2D) will be assessed by escalation with overdose control (EWOC), an adaptive Bayesian design as described below using the dosing schema proposed in **Table 1**.

This is a single-arm, open-label, phase I trial to determine the safety and feasibility of the combination of L-glutamine, gemcitabine, and nab-paclitaxel in untreated, metastatic pancreatic cancer.

Once patient is deemed eligible to be enrolled onto the study, the subject will proceed to Baseline for a 3–7-day lead-in, per Investigator's discretion, where patients will receive L-glutamine (0.1, 0.2, or 0.3 g/kg oral twice daily).

Table 1. Dosing schema for Phase I GlutaPanc Trial.

**Dose Level**[a]	**Gemcitabine**	**Nab-paclitaxel**	**L-glutamine**[b]
-2	600 mg/m^2 IV D1, 8, 15	75 mg/m^2 IV D1, 8, 15	0.1 g/kg oral BID
-1	800 mg/m^2 IV D1, 8, 15	100 mg/m^2 IV D1, 8, 15	0.1 g/kg oral BID
0	1000 mg/m^2 IV D1, 8, 15	125 mg/m^2 IV D1, 8, 15	0.1 g/kg oral BID
1	1000 mg/m^2 IV D1, 8, 15	125 mg/m^2 IV D1, 8, 15	0.2 g/kg oral BID
2	1000 mg/m^2 IV D1, 8, 15	125 mg/m^2 IV D1, 8, 15	0.3 g/kg oral BID

[a]Every 28-day cycles
[b]Rounded to nearest 5 g, upper limit of 15 g twice daily (30 g/day) for any dose level; started 1 week (+/- 1 day) prior to chemotherapy
BID: twice a day

This phase I study is designed to identify the maximum tolerated dose (MTD) using 5 dose levels as defined in **Table 1**. The MTD is defined as the dose level such that the probability of DLT at the MTD is $\theta=0.33$. We will use an extension of escalation with overdose control (EWOC) algorithm to design the trial.67 The first patient will receive study drug at dose level 0 and the dose for the next patient will be determined according to the EWOC algorithm (see **Operating Characteristics**). At any time during the trial, a patient may be enrolled to the trial if there are no more than 2 patients with unresolved DLT status in the trial. In other words, if there are 3 patients under treatment that did not experience DLT and have not finished their cycle 1 therapy, then we cannot enroll another patient.

Evaluation of the RP2D and DLTs will be based on the first 4 weeks (Cycle 1) of study drug treatment. The RP2D is defined as the dose level closest to the median of the posterior distribution of the MTD.

One treatment cycle of the combination of L-glutamine, gemcitabine, and nab-paclitaxel will consist of L-glutamine continuous oral daily dosing along with gemcitabine and nab-paclitaxel IV on days 1, 8, and 15 (every 28-day cycles) each given according to the dosing scheme (**Table 1**). A cycle will be defined around the start of gemcitabine and nab-paclitaxel i.e., Day 1 of each 28-day Cycle of gemcitabine and nab-paclitaxel. Treatment will be administered on an outpatient basis.

Post-treatment imaging and follow up will be performed 8 weeks after initiating therapy (C1D1). Patients removed from study for unacceptable adverse events will be followed until resolution or stabilization of the adverse event. Subjects who cannot complete a minimum of 3 days during the 3-7 day lead-in of L-glutamine alone may be replaced per Investigator's discretion. The start of a subsequent cycle can be delayed depending on toxicities of the prior cycle.

Operating Characteristics. For the purpose of deriving the operating characteristics of the study, we will assume that successive cohorts of 1 patient will be treated and the dose level for the first patient will be dose level 0. The dose for each subsequent patient will be determined so that, based on all available data, the probability that it exceeds the MTD is equal to a prespecified value α. In this trial, we start at α=0.4 and increase α in small increments of 0.05 until α=0.5, this value being a compromise between the therapeutic aspect of the agent and its toxic side effects. No dose skipping will be permitted. **The trial will be terminated if the posterior probability that the probability that DLT at dose level -2 exceeds θ=0.8 or more.**

A maximum of 16 patients will be accrued to the trial. Upon completion of the trial, the RP2D will be estimated as the median of the marginal posterior distribution of the MTD rounded to the nearest discrete dose level. The computation of the dose to be administered to each patient and the estimate of the MTD will be carried out by the biostatistician using R and JAGS.

We evaluated the design operating characteristics of this algorithm under 3 scenarios for the true value of the MTD. **Table 2** shows various summary statistics such as the percent of MTD recommendation using 1000 trial replicates, average % of DLTs across all trials, % of trials with an excessive rate of DLT, and % of patients allocated to each dose level. For each scenario, the true MTD is indicated in bold (probability of DLT=0.33). All 3 scenarios are misspecified so that the results of the simulations do not depend on the logistic model. In scenario 1, the MTD is dose level -2. In this case, the probability of recommending this dose level is 0.816 and 58% of the patients are allocated to that level. The average percent of DLT is 42.5%, about 10% higher than the target which is expected when the minimum dose level is the MTD. In scenario 2, the MTD is dose level 0. In this case, the percent of recommendation is 57.3% and the trial is safe. In scenario 3, the MTD is the last dose level. In this case, the percent MTD recommendation is 61.3% and 93.7% of the trial will recommend either dose level 1 or 2 as the true MTD. Most patients are allocated to the MTD and the trial is very safe. Based on these scenarios, we conclude that the design performs well with a sample size of 16 patients.

Table 2. Design Operating Characteristics for Safety and Efficiency (true MTD shown in bold)

	Dose Level				
**Scenario 1**	**-2**	-1	0	1	2
True Prob DLT	**0.33**	0.45	0.65	0.75	0.90
%MTD recommendation	**81.6**	17.7	7.0	0.0	0.0
% patients allocated	57.9	26.1	12.1	3.3	0.6
Ave % DLTs	42.5				
% Trials: rate DLT > θ+0.1	57.5				
	Dose Level				
**Scenario 2**	-2	-1	**0**	1	2
True Prob DLT	0.01	0.05	**0.33**	0.50	0.70
%MTD recommendation	0.0	28.0	**57.3**	13.9	0.8
% patients allocated	2.5	25.5	46.1	18.4	7.2

Ave % DLTs	30.1				
% Trials: rate DLT > θ+0.1	5.1				
	Dose Level				
**Scenario 3**	-2	-1	0	1	**2**
True Prob DLT	0.001	0.03	0.08	0.15	**0.33**
%MTD recommendation	0.00	0.20	0.61	32.4	**61.3**
% patients allocated	0.50	2.73	14.95	27.37	54.42
Ave % DLTs	23.08				
% Trials: rate DLT > θ+0.1	0.50				

On review of our annual Cedars-Sinai Medical Center (CSMC) gastrointestinal oncology (GI) Oncology Program census, the PI and co-investigators see at minimum 4 new pancreatic ductal adenocarcinoma (PDAC) cases per month where the majority (approximately 60%) are unresectable or metastatic at presentation in our high-volume GI oncology clinic at CSMC. Therefore, we expect to be able to accrue 16 patients with advanced pancreatic cancer over 12 months for DLT assessment and longitudinal collection of blood samples, accounting for screen failures. Survival and exploratory analyses would occur over the subsequent 12 months. We estimate the total study duration to be 24 months.

All eligible patients who are enrolled into the study and receive at least one dose of the study drug (the Safety Population) will be included in the safety and preliminary efficacy analysis. All enrolled subjects will undergo peripheral blood draws for plasma metabolomics and if available, stool collection for microbiome analyses. Those having received study drug will be included in the exploratory biomarker analyses.
