# Supplementary material for: l-Glutamine in combination with first-line gemcitabine and nab-paclitaxel in advanced pancreatic ductal adenocarcinoma: an open-label, single-arm, phase 1 GlutaPanc trial
Source: Nat Cancer. 2026 Aug 31;7(9):1406–18. doi: 10.1038/s43018-026-01225-z (PMC13600916; doi:10.1038/s43018-026-01225-z)
Supplement: Supplementary file 2 [file 43018_2026_1225_MOESM2_ESM.pdf]

nature portfolio

Corresponding author(s): Jun Gong, Neil Bhowmick

Last updated by author(s): May 15, 2026

Reporting Summary

Nature Portfolio wishes to improve the reproducibility of the work that we publish. This form provides structure for consistency and transparency in reporting. For further information on Nature Portfolio policies, see our Editorial Policies and the Editorial Policy Checklist.

Statistics

For all statistical analyses, confirm that the following items are present in the figure legend, table legend, main text, or Methods section.

n/a	Confirmed	
☐	☒	The exact sample size (*n*) for each experimental group/condition, given as a discrete number and unit of measurement
☐	☒	A statement on whether measurements were taken from distinct samples or whether the same sample was measured repeatedly
☐	☒	The statistical test(s) used AND whether they are one- or two-sided<br>*Only common tests should be described solely by name; describe more complex techniques in the Methods section.*
☐	☒	A description of all covariates tested
☐	☒	A description of any assumptions or corrections, such as tests of normality and adjustment for multiple comparisons
☐	☒	A full description of the statistical parameters including central tendency (e.g. means) or other basic estimates (e.g. regression coefficient) AND variation (e.g. standard deviation) or associated estimates of uncertainty (e.g. confidence intervals)
☒	☐	For null hypothesis testing, the test statistic (e.g. *F*, *t*, *r*) with confidence intervals, effect sizes, degrees of freedom and *P* value noted<br>*Give P values as exact values whenever suitable.*
☐	☒	For Bayesian analysis, information on the choice of priors and Markov chain Monte Carlo settings
☒	☐	For hierarchical and complex designs, identification of the appropriate level for tests and full reporting of outcomes
☒	☐	Estimates of effect sizes (e.g. Cohen's *d*, Pearson's *r*), indicating how they were calculated

Our web collection on statistics for biologists contains articles on many of the points above.

Software and code

Policy information about availability of computer code

Data collection	No software was used
Data analysis	No software was used

For manuscripts utilizing custom algorithms or software that are central to the research but not yet described in published literature, software must be made available to editors and reviewers. We strongly encourage code deposition in a community repository (e.g. GitHub). See the Nature Portfolio guidelines for submitting code & software for further information.

Data

Policy information about availability of data

All manuscripts must include a data availability statement. This statement should provide the following information, where applicable:

- Accession codes, unique identifiers, or web links for publicly available datasets
- A description of any restrictions on data availability
- For clinical datasets or third party data, please ensure that the statement adheres to our policy

All data needed to evaluate the conclusions in the paper are present in the paper and/or the Supplementary Materials. The metabolomics data generated in this study will be deposited into the MassIVE public repository (Dataset MSV000095856). The raw metagenomic sequencing data generated in this study have been deposited in the NCBI Sequence Read Archive (SRA) under project accession number PRJNA1463891. Source data for Fig. 1-7 and Extended Data Fig. 2-4 and Extended Data Tables 2-3 have been provided as Source Data files. Additional data related to this paper may be requested from the authors. De-identified data not

published including study protocol are available for transfer following submission of a data transfer agreement (DTA) to the PI (jun.gong@csmc.edu). Published data will be made available for as long as permitted within journal policies. Preserved but unshared data will be made available as long as permitted by the public repository and/or institutional policy. Proposals will be vetted by the Cedars-Sinai Medical Center Cancer Clinical Trials Office within a timeframe of 60 days. Investigators and institutions who consent to the terms of the DTA form, including but not limited to the use of these data for the purpose of a specific project and only for research purposes, and to protect the confidentiality of the data and limit the possibility of identification of participants for the duration of the agreement, will be granted access.

Research involving human participants, their data, or biological material

Policy information about studies with human participants or human data. See also policy information about sex, gender (identity/presentation), and sexual orientation and race, ethnicity and racism.

Reporting on sex and gender	Findings to this study apply for all sexes and genders. Sex and/or gender was determined by self-reporting. Number and age of participants are reported in the source data for every experiment, when collected and available. Similarly, information on disaggregated sex and gender data has been provided in the source data, when collected. Informed consent was obtained by participants. There was no participant compensation provided. Sex- and gender-based analyses not performed due to nature of phase I trial design with a limited cohort of patients to be enrolled to meaningfully conclude sex- and gender-driven factors.
Reporting on race, ethnicity, or other socially relevant groupings	Race but not ethnicity is reported in this study. Race was determined by self-reporting.
Population characteristics	Population characteristics are reported in Table 1. Median age was 71, prior history of cachexia at study entry was reported including past surgery and site of metastases have been reported, but no other comorbidities are reported.
Recruitment	Eligible subjects with metastatic pancreatic cancer were referred to our gastrointestinal (GI) oncology team at CSMC. Patients referred to our high-volume GI oncology clinic represent an economically, racially, and ethnically diverse group of participants from within the CSMC health network and outside the network from providers in the community.
Ethics oversight	Approved by Cedars-Sinai IRB with regulatory oversight provided by the Cedars-Sinai Medical Center Cancer Clinical Trials Office. This study has been approved by the Institutional Review Board of Cedars-Sinai Medical Center and informed consent was obtained from all subjects involved in the study. This study and its design was conducted according to the guidelines of the Declaration of Helsinki.

Note that full information on the approval of the study protocol must also be provided in the manuscript.

Field-specific reporting

Please select the one below that is the best fit for your research. If you are not sure, read the appropriate sections before making your selection.

☒ Life sciences ☐ Behavioural & social sciences ☐ Ecological, evolutionary & environmental sciences

For a reference copy of the document with all sections, see nature.com/documents/nr-reporting-summary-flat.pdf

Life sciences study design

All studies must disclose on these points even when the disclosure is negative.

Sample size	Cancer phase I trials are typically designed to estimate the maximum tolerated dose (MTD) or recommended phase 2 dose (RP2D). As such, they are not designed to test a specific hypothesis or powered to favor a particular alternative hypothesis. The performance of such designs is evaluated using operating characteristics under different scenarios for the location of the true MTD. In our trial, the operating characteristics were reasonable for a sample size of 16, which established the sample size of 16 for this dose-finding phase I trial based on adaptive Bayesian design.
Data exclusions	18 subjects were enrolled into this phase I clinical trial, however, 2 of the subjects were deemed non-evaluable for the dose-limiting toxicity assessment and were replaced with 2 new subjects, bringing final cohort to n=16.
Replication	Dose-finding nature of this phase I trial where single patients are enrolled into various dosing combinations preclude replication, however, advancement of these positive findings into subsequent phase II or III trials would be warranted to confirm safety and efficacy signals seen in this trial.
Randomization	No randomization was performed as this was a single-arm, phase I dose-finding trial
Blinding	No blinding was performed as this was an open-label, single-arm, phase I dose-finding trial

Reporting for specific materials, systems and methods

We require information from authors about some types of materials, experimental systems and methods used in many studies. Here, indicate whether each material, system or method listed is relevant to your study. If you are not sure if a list item applies to your research, read the appropriate section before selecting a response.

Materials & experimental systems

n/a	Involved in the study	
☐	☒	Antibodies
☒	☐	Eukaryotic cell lines
☒	☐	Palaeontology and archaeology
☒	☐	Animals and other organisms
☐	☒	Clinical data
☒	☐	Dual use research of concern
☒	☐	Plants

Methods

n/a	Involved in the study	
☒	☐	ChIP-seq
☒	☐	Flow cytometry
☒	☐	MRI-based neuroimaging

Antibodies

Antibodies used	Lipopolysaccharide/LPS (Sandwich ELISA) ELISA Kit - LS-F17912 (LifeSpan Biosciences/ LS-BIO, WA) and Human XL Cytokine Luminex® Performance Assay 46-plex Fixed Panel (Cat# LKTM014B) from R&D Systems Inc. (Minneapolis, MN) for Luminex 200
Validation	A sandwich enzyme-linked immunosorbent assay (ELISA) was performed using the Lipopolysaccharide/LPS (Sandwich ELISA) ELISA Kit - LS-F17912 (LifeSpan Biosciences/ LS-BIO, WA). Controls, standards, blanks, and samples, following ten-fold dilution. Sample concentrations were calculated following standard curve generation using a 4-parameter logistic regression and compared by the paired samples Wilcoxon test ($P < 0.05$ being statistically significant). A panel of 46 cytokines were analyzed using a multiplex bead-based immunoassay, Human XL Cytokine Luminex® Performance Assay 46-plex Fixed Panel (Cat# LKTM014B) from R&D Systems Inc. (Minneapolis, MN) for Luminex 200. Plasma samples were passively thawed, centrifuged at 16,000 x g for 4 minutes immediately prior to diluting two-fold with R&D Assay Diluent RD6-65 and tested in duplicate. A seven-point standard curve across the 5-log dynamic range of the assays was included in the current assay design. Both the ELISA LPS assay and multiplex bead-based immunoassay have undergone extensive validation testing and can be referred to based on the individual product data sheets per the manufacturer.

Clinical data

Policy information about clinical studies

All manuscripts should comply with the ICMJE guidelines for publication of clinical research and a completed CONSORT checklist must be included with all submissions.

Clinical trial registration	NCT04634539
Study protocol	Full study protocol uploaded with submission per Editor's request
Data collection	Single-institution, Cedars-Sinai Medical Center. Period of enrollment. May 13, 2021 to June 2, 2023
Outcomes	Recommended phase II dose was primary outcome; efficacy and safety were secondary outcomes; all pre-defined by adaptive Bayesian design and efficacy by RECIST criteria, safety by CTCAE criteria

Plants

Seed stocks	No plants were used in this study.
Novel plant genotypes	No plants were used in this study.
Authentication	No plants were used in this study.
