# Supplementary material for: l-Glutamine in combination with first-line gemcitabine and nab-paclitaxel in advanced pancreatic ductal adenocarcinoma: an open-label, single-arm, phase 1 GlutaPanc trial
Source: Nat Cancer. 2026 Aug 31;7(9):1406–18. doi: 10.1038/s43018-026-01225-z (PMC13600916; doi:10.1038/s43018-026-01225-z)
Supplement: Supplementary file 3 [file 43018_2026_1225_MOESM3_ESM.pdf]

nature portfolio

Peer Review File

L-glutamine in combination with first-line gemcitabine and nab-paclitaxel in advanced pancreatic ductal adenocarcinoma: an open-label, single-arm, phase 1 GlutaPanc trial

Corresponding Author: Dr Jun Gong

This manuscript has been previously reviewed at another journal. This document only contains information relating to versions considered at Nature Cancer.

This file contains all reviewer reports in order by version, followed by all author rebuttals in order by version.

Version 0:

Reviewer comments:

Reviewer #1

(Remarks to the Author)
The authors performed a phase 1 dose finding study identifying the maximum tolerated dose of L-glutamine in combination with gemcitabine and abraxane for patients with advanced pancreatic cancer. They discovered that the amino acid is well-tolerated with chemotherapy and observed correlative signals indicating activity in the form of changed circulating plasma and stool levels of metabolites and other markers. This is a novel study and very relevant. The secondary endpoints of survival were impressive and promising compared to historical controls. I commend the trial investigators on the hypothesis, study design and execution of the study. The statistics and analysis are sound.

I have several questions and comments which I consider to be mostly minor.

Major
1) Were trial participants metastatic only or locally advanced- this is confusing. I thought it was only metastatic based on the results but then 1A implies locally advanced was included. This was also mentioned in the methods. If so, how many were locally advanced? And the expected survival is different in this cohort, such that the reference used for historical controls should be changed and the interpretation of the secondary endpoint may need to be adjusted.

Minor

1) An editing suggestion: "Broadly, those patients having longer survival to glutamine based therapy had reduced abundance of gram-negative bacteria at baseline and after L glutamine treatment" might want to be edited to "....experiencing longer survival on glutamine based therapy ..."

2) Is 7 days enough to see changes in microbiome? What if you did a 2 week or 3 week lead-in? Would that be ethical to delay chemo by that long and if so, would that have made a difference in your alpha diversity effects?

3) An editing suggestion: I know what your saying here but you could write a better sentence: "However, with higher gram-negative bacteria content in the gut of short OS patients under basal conditions, glutamine being the preferred nitrogen source resulted in greater respiration." Along those lines, please be specific as to what you mean by respiration and how you determined that.

4) It is confusing when you write about microbiome changes 'in the presence of GA' because only blood was taken after GA. There were no stool samples taken at after the first dose of GA.

5) Anything special about GA? Why not FOLFIRINOX? Why did you choose GA in this trial?

6) Do you have drug diaries or data on compliance with L-glutamine receipt? If so, please report. If patients were not compliant, the data would be interpreted differently.

7) What is your interpretation or understanding why short survivors had greater alpha diversity? Please explain.

8) No difference in Shannon index (Fig. 5A) but there does seem to be a consistent trend across each analysis towards an increase with L-glutamine (interestingly in the overall and in the long-term survivors, but not the short-term). Please comment whether you believe this is worthy of further analysis and I would recommend if so, you point this out in the text. There is nothing magical about the threshold of p=0.05. While accepted as statistical significant, it's arbitrary, so this may be a type 2 error that could be supported with a larger sample size and would that support your hypothesis?

9) Do the bars in Ex Fig 5C correspond on a per patient level? That is, is the first bar in baseline and and L-glut the same patient and so on? Same question for D. If so, doesn't it show microbiome migration in all cases? Please comment.

10) Please explain Ext Data Fig 7 better. Why are many gene families replicated in the list?

Reviewer #2

(Remarks to the Author)
The authors address an important and timely clinical question, and the overall study is of potential significance. Nevertheless, despite the authors' revisions, several statistical aspects of the analyses presented in Extended Data Figs. 4 and 5 remain insufficiently clarified, particularly with respect to sample definition, subgroup stratification, and analytical transparency.

First, the effective sample sizes contributing to each analysis are still not clearly reported. While Extended Data Fig. 5 indicates n = 12, it is not explicitly stated how these patients relate to the full evaluable cohort (n = 16), nor whether inclusion was determined solely by biospecimen availability. In addition, the numbers of patients contributing to each subgroup comparison (e.g., long vs. short OS or PFS) are not specified. Explicit reporting of subgroup sizes in the figures or legends is necessary for proper interpretation, particularly given the small cohort size.

Second, in Extended Data Fig. 4, plasma metabolites were selected and stratified into "high" and "low" groups based on associations with overall survival, followed by Kaplan–Meier visualization. While Kaplan–Meier methods are appropriate for descriptive estimation of PFS and OS in this phase I study, the outcome-informed nature of metabolite selection and stratification should be more clearly acknowledged, as this approach is inherently exploratory and may introduce optimistic bias in small cohorts. The absence of multiple-testing adjustment further underscores the need to frame these analyses as hypothesis-generating rather than prognostic.

Overall, clearer reporting of sample selection, subgroup sizes, and analytical boundaries—together with consistently cautious framing of outcome-associated findings as exploratory—would substantially strengthen the rigor and interpretability of the results presented in Extended Data Figs. 4 and 5.

Version 1:

Reviewer comments:

Reviewer #1

(Remarks to the Author)
The authors have addressed all concerns of this reviewer.

Reviewer #2

(Remarks to the Author)
I have no further specific comments on the manuscript. One remaining point concerns the presentation of sample sizes in the figures. Given the very limited sample size used in these analyses (only 15 and 12 patients in the respective cohorts), it is important that the exact number of samples in each subgroup be explicitly reported in the relevant figures rather than only providing the total number analyzed. Although the authors note that a larger cohort will be required to further assess the biomarker potential of these metabolites, the current presentation still needs to be more transparent. For example, the Kaplan–Meier curves in Extended Data Fig. 4 should indicate the group sizes directly in the labels (e.g., High (n = xx), Low (n = xx)), and the same applies to the grouped boxplots in Extended Data Fig. 5a. Clear reporting of per-group sample sizes is necessary for readers to evaluate the strength and stability of the observed associations.

Open Access This Peer Review File is licensed under a Creative Commons Attribution 4.0 International License, which permits use, sharing, adaptation, distribution and reproduction in any medium or format, as long as you give appropriate credit to the original author(s) and the source, provide a link to the Creative Commons license, and indicate if changes were made.
In cases where reviewers are anonymous, credit should be given to 'Anonymous Referee' and the source.
The images or other third party material in this Peer Review File are included in the article's Creative Commons license, unless indicated otherwise in a credit line to the material. If material is not included in the article's Creative Commons license and your intended use is not permitted by statutory regulation or exceeds the permitted use, you will need to obtain permission directly from the copyright holder.
To view a copy of this license, visit https://creativecommons.org/licenses/by/4.0/

<u>Reviewer #1 (Revision History)</u>

NMED-BC132311D

Reviewer #1 (Remarks to the Author):

Description of clinical trial:

This was a single-arm, dose-finding phase I trial where all subjects completed a 1-week oral L-81 glutamine lead-in prior to addition of GA and evaluation of the primary endpoint (RP2D). All 16 subjects enrolled completed the L-glutamine lead-in and received a median of 8 cycles (range 2-83 15) of GA and L-glutamine.

The primary objective was to determine the recommended phase II dose (RP2D) using escalating doses of L-glutamine in combination with standard gemcitabine and nab-paclitaxel (GA). Secondary objectives included assessing early signals of efficacy and impact on the gut microbiome composition, inflammatory response, and metabolomic profile

Overall, this manuscript is well written. The statistical components (design and analysis plan) agree well with clinical protocol. The interpretation of statistical analysis results is well supported. No formal statistical testing was performed. No major issues have been discovered.

1. Regarding endpoints:

The Primary Endpoint in clinical protocol is the number of dose-limiting toxicities (DLTs), defined as the rate of drug-related grade ≥ 3 adverse events (AEs) experienced within the first 4 weeks (1 cycle) of study treatment. The RP2D is defined as the dose level closest to the median of the posterior distribution of the maximum tolerated dose (MTD). The MTD is defined as the dose level such that the probability of DLT at the MTD is $\theta = 0.33$.

The Secondary Endpoints in clinical protocol are 1) Adverse events as summarized in terms of type and severity by NCI CTCAE version 5.0; 2) Clinical activity of the combination including objective response rate (ORR), progression-free survival (PFS), and overall survival (OS) evaluated every 8 weeks (every 2 cycles ±1 week) according to the revised RECIST guidelines (version 1.1)

The study endpoints (both the primary and the secondary) reported in the manuscript are as specified as in the protocol.

2. Regarding the appropriateness of statistical analysis and suitability of the descriptions of statistical analysis plan:

The recommended phase II dose (RP2D) was assessed by EWOC, an adaptive Bayesian design, using the dosing schema proposed in Table S2. EWOC, known as “escalation with overdose control”, is a phase I design introduced in recent years (Tighiouart and Bogatko, Statistical Science, 2010). The Bayesian design and analysis have been increasingly used in clinical trials. **The use of EWOC in this trial design is appropriate and the statistical analysis plan for the primary end point is suitable.**

Regarding the secondary endpoints, as the goal was to characterize the preliminary therapeutic efficacy of the combination of gemcitabine, nab-paclitaxel, and L-glutamine, no formal hypothesis testing was conducted. The sample estimates of complete response (CR), partial response (PR), median overall survival, median progression-free survival (PFS) are reported along with confidence intervals. **Such statistical analysis plan for the secondary end points is appropriate and the description of statistical analysis is suitable.**

3. Regarding whether the interpretation of the results is supported by the data and statistics:

For dose-finding and safety (primary endpoint): “The trial was closed once 16 patients were evaluable for DLT and the RP2D was estimated as the median of the posterior distribution of the MTD, which was found to be dose level 2 corresponding to maximum doses of oral L-glutamine (30 g/day 96 orally) with full standard doses of gemcitabine (1000 mg/m2) and nab-paclitaxel (125 mg/m2). ” For efficacy outcomes (secondary endpoint), no formal hypothesis testing was conducted. **Such interpretation of the results is supported by the data and statistics.**

We thank the reviewer for this thorough critique of our manuscript. We are appreciative of the reviewer’s overall approval of our study design, endpoints, statistical analysis, and interpretation of the results.

NMED-BC132311E-Z

Reviewer #1 (Remarks to the Author):

I have no further comments regarding statistical components.

We thank the reviewer for approving our revised manuscript.

Reviewer #2 (Revision History)

NMED-BC132311D

Reviewer #2 (Remarks to the Author):

The investigators conduced a single-armed, phase 1, dose finding study evaluating administration of 1 week of oral L-glutamine in combination with gemcitabine and nab-paclitaxel, as a first line treatment for metastatic pancreatic cancer. There were no treatment related study discontinuations. The primary endpoint was to find the RPIID. Secondary endpoints included efficacy analyses and microbiome related studies. Patients were eligible if they had unresectable or advanced disease.
The optimal dose was found to be 30 g L glutamine per day. Most treatment related adverse events were related to the chemotherapy.

We thank the reviewer for taking the time to review our manuscript. We provide a point-by-point response to critiques and comments below.

B. The study's originality is modest, with numerous studies in multiple cancers evaluating the varied impact of L-glutamine.

Glutamine Supplementation as an Anticancer Strategy: A Potential Therapeutic Alternative to the Convention. Cancers (Basel). 2024 Mar 5;16(5):1057. doi: 10.3390/cancers16051057

We appreciate the reviewer for their comments on study originality and for citing our review of glutamine supplementation as an anticancer strategy. Although we highlight in our review the numerous studies across multiple cancers for which L-glutamine has been evaluated, we would like to point out that majority of prospective trials investigated L-glutamine supplementation as an adjunctive therapy to mitigate toxicities in cancer patients receiving chemotherapy and radiation therapy. Clinical translation of L-glutamine administration within the context of cancer therapy has not been formally attempted. Furthermore, none of these former studies investigated an FDA-approved, clinical grade L-glutamine as we have, for which we believe adds to the originality of our study.

C. D. The data are comprehensive and the correlatives measured are deep in the assessments. They are presented in a confusing way, generally with the presentation of - and + L-glutamine combined with PFS and OS responder data really confusing and difficult to understand and interpret. Statistical analyses are appropriately presented.

We thank the reviewer for commenting on the comprehensiveness of the data and appropriateness of the statistical analyses. In Extended Data Fig. 4a (updated to Extended Data Fig. 5a in final version), we have corrected to include a figure depicting alpha diversity. In Extended Data Figure 5 (Extended Data Figure 6 in final version), we have made clearer the comparisons were between the long and short PFS subjects in Extended Data Figure 5a (Extended Data Figure 6a in final version) and between the long and short OS subjects in Extended Data Figure 5b (Extended Data Figure 6b in final version) that significantly differed among those administered glutamine. We have included a description of these findings in the main text with reference to these figures for more clarity.

E. The primary endpoint of safety seems to be reached. The extraordinary efficacy outcomes achieved are impossible to attribute to the l-glutamine and it seems implausible based on the prior literature and the modest rationale presented in the text. It is more likely a sample size and selection bias phenomenon. If the authors feel otherwise, the rationale of how L-glutamine will improve chemo-response needs to be explained much more deeply.

We understand the reviewer's concerns on the extraordinary efficacy outcomes demonstrated in our trial. We agree that the small sample size and single-arm, phase I nature of our study limits definitive conclusions regarding the efficacy of the study combination. However, the rationale of how L-glutamine improves treatment outcomes appears multifold. An aspect of the mechanisms of glutamine's effects on improving chemotherapy efficacy appears to occur through amino acid depletion in cancer cells. The biochemistry of glutamine transporter occupation of tumor cells by high dose glutamine resulting in amino acid depletion was deemed to be outside the scope of this brief report; but we have included a copy for the reviewers along with a key figure shown below. Given that serial stool and blood specimens were collected as part of preplanned correlatives in this study, we focused the results on the role of stool bacterial metagenomics and glutamine supplementation's impact on systemic metabolomic response that seem to be key to our observations of extended OS of the patients here. We note an additional potential mechanism of L-glutamine's benefit as an anti-cachexia therapy that warrants further exploration. Specifically, a post-hoc analysis at the request of another reviewer identified that increases in weight and BMI as well as weight stability was achieved in 50% of patients treated with L-glutamine in this study (median duration of treatment 8.6 months), the majority of whom were cachectic at study entry. It stands to reason that the achieving weight stability would enable greater chemotherapy tolerability, contributing to greater tumor responsivity.

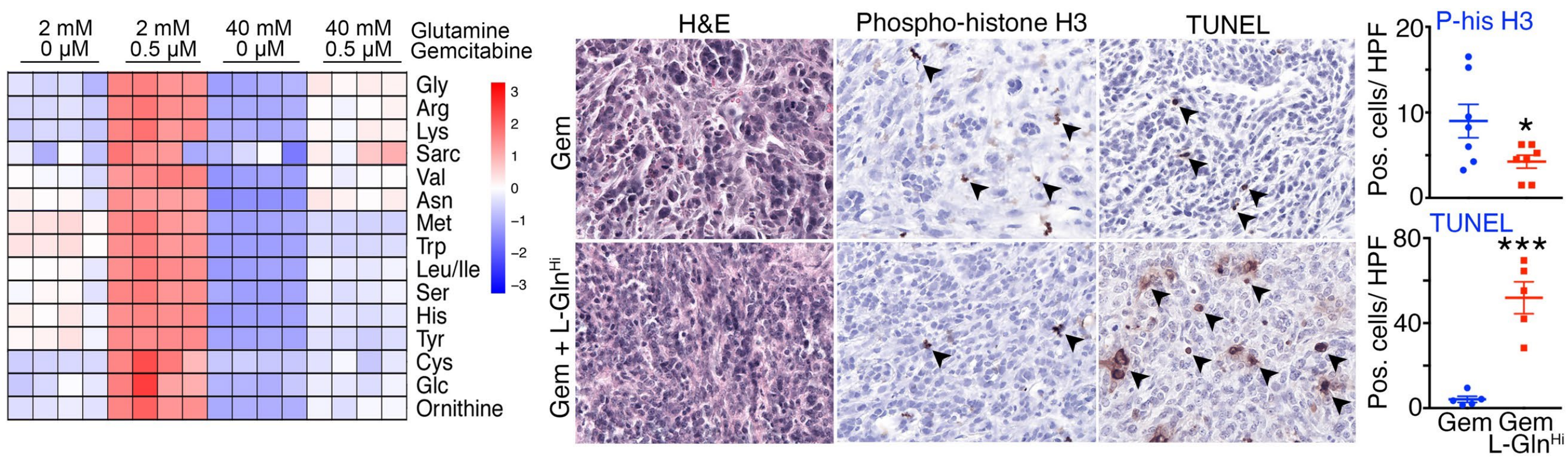

Glutamine supplementation to gemcitabine treatment results in amino acid depletion and tumor regression. Heatmap shows an enrichment of amino acids in cultured PDAC cells (MIAPaCa2) surviving gemcitabine treatment that is restored to near control levels when high glutamine levels are administered in combination (Left). Syngeneic mice orthotopically grafted with PDAC cells (KPC961) were treated with gemcitabine alone or in combination with glutamine. Combination therapy resulted in a significant increase in apoptosis and decrease in mitosis compared to gemcitabine alone (Right).

In looking at the study population for other possible explanations to these results and the possibility of selection bias, we note that the demographics and baseline characteristics appear reflective of what we see in the clinic. For example, the median age being 71, the majority (81%) meeting consensus criteria for cachexia at study entry, the majority (75%) having de novo unresectable or metastatic disease, and the sites of metastases with liver and lung being most common at presentation (68%). We have also provided additional data on post-progression therapies in our cohort at the request of the reviewer (see Table below). We believe this data further reinforces the benefit driven by first-line study treatment in our cohort, rather than that driven by post-progression therapies. Ultimately, we were careful to provide a closing statement in the manuscript that these findings warrant advancement to a randomized controlled trial to validate our phase I results. The dismal prognosis of advanced pancreatic cancer and the high unmet need for more effective systemic therapies prompted the decision to communicate our promising results as a brief report for rapid dissemination. This is more relevant given the exquisite safety and non-overlapping toxicity observed with L-glutamine to standard chemotherapy and the availability of a pharmaceutical grade, FDA-approved compound that is readily available for translation into late-phase clinical trials.

F. Generally, the data should be explained better in the supplemental figures to better support the authors' premise. They are difficult to follow and interpret in a way that drives home the hypothesis that L-glutamine supplementation is a promising therapy. It is unclear why glutamine and glutamic acid levels should not have changed with treatment. The most helpful data and compelling data would be simple changes in diversity with L-glutamine lead in treatment, but I don't see those data simply presented but rather the response data mixed in make it more difficult to understand.

We understand the reviewer's concerns regarding our initial finding that plasma glutamine/glutamate levels were unchanged to treatment. We initially presented one concern based on recent prospective data from healthy subjects and subjects with sickle cell anemia receiving up to 0.3 g/kg of oral L-glutamine twice daily (consistent with our maximum doses of oral L-glutamine) that showed rapid absorption of L-glutamine from the bloodstream after administration. For example, the mean half-life of plasma L-glutamine was 1 hour, mean peak plasma concentrations of L-glutamine occurred at 1.2 hours, and L-glutamine plasma concentrations returned to baseline within a few hours across all doses of oral L-glutamine (PMID: 38401036). We did not find that glutamine affected stool bacterial diversity, however, glutamine impacted stool metabolites and systemic inflammation.

Of note, we subsequently presented further analysis involving a primary comparison of amino acid levels in our patient blood samples that were not previously available at the time of this revision. Although not available to the reviewer during this revision round, we later were able to demonstrate that L-glutamine therapy significantly elevated plasma glutamine concentrations (please see response to Reviewer #2 NMED-BC132311E-Z below where this new data was presented).

How can median PFS be 8.5 months, but the 12 month PFS rate be 69%?

We thank the reviewer for pointing out this mistake. This was meant to be a 6-month PFS rate of 69% and 12-month PFS rate of 25%. We have corrected this in the manuscript.

The dosing schedule should be better detailed and explained in the body of text.

We have provided a more detailed explanation of the dosing schedule in the "Dose-finding and safety" section in the main body of text, as suggested.

Data related to second line and third line treatments should be detailed, with PFS rates in those lines. The reason is it is that readers will want to understand if the surprising
OS rates could be driven by the second line data.

Data on second-line and beyond treatments have been included in the following table, which is Extended Data Table 4 in the revised manuscript:

Post-progression therapies and progression-free survival

**Characteristic (n=14)[a]**	**n (%)**
Lines of therapy	
1[b]	2 (14%)
2	5 (36%)
3	2 (14%)
4	5 (36%)
Post-progression regimens	
<u>Second-line (n=12):</u>	
FOLFIRINOX	5 (42%)

5-FU/liposomal irinotecan	4 (33%)
Gemcitabine/capecitabine	2 (17%)
NALIRIFOX	1 (8%)
Third-line (n=7):	
Gemcitabine/nab-paclitaxel	1 (14%)
FOLFIRINOX	1 (14%)
IROX	1 (14%)
Clinical trial	4 (58%)
Fourth-line (n=5):	
Cisplatin/gemcitabine	2 (40%)
Cisplatin/gemcitabine/nab-paclitaxel	1 (20%)
FOLFOX	1 (20%)
Clinical trial	1 (20%)
Post-progression PFS (median, range)	
Second-line PFS	2 months (0.7-13.2 months)
Third-line PFS	1.6 months (0.5-4.3 months)
Fourth-line PFS	1.2 months (0.7-3.0 months)

[a]Two subjects did not have disease progression at time of data cut off
[b]Two subjects did not receive subsequent systemic therapies upon progression to study treatment
FOLFIRINOX, 5-fluorouracil or 5-FU, leucovorin, irinotecan, oxaliplatin; NALIRIFOX, liposomal irinotecan, 5-FU, leucovorin, oxaliplatin; IROX, irinotecan and oxaliplatin; FOLFOX, 5-FU, leucovorin, oxaliplatin; PFS, progression-free survival

As evidenced by the post-progression data, although 50% of the subjects were able to receive third- and fourth-line therapies, the benefit was short-lived with median PFS < 2 months. The majority of the benefit was driven by first-line study treatment (median PFS 8.5 months). Of note, one subject had the longest PFS of 25 months to study treatment with a KRAS^{Q61H} mutation that arguably confers the worst prognosis out of all KRAS mutation subsets in PDAC (PMID: 38310130). The second longest PFS (19.8 months) to L-glutamine, gemcitabine, and nab-paclitaxel was seen in a patient with a KRAS wild-type and BRAF^{V600R}-mutant tumor. Two other subjects with >12 months PFS had KRAS^{G12V} and KRAS^{G12R} mutations, with one of these two subjects having co-occurring mutations in BRCA1/2. The median PFS of the study combination may underestimate the duration of benefit given that the mean PFS to first-line study treatment in our cohort was 10.7 months. We have added a brief discussion on this important topic in the revised manuscript.

The interpretation “The addition of oral 150 L-glutamine to GA improved PFS, nearly doubled the ORR and quadrupled the 24-month OS compared to historical first-line GA alone in metastatic PDAC” must be tempered. In this single armed study, one can not conclude causality and state that the intervention ‘improved’ anything. It was associated with longer…. then historical experience in this relatively small cohort.

We have modified the conclusion to temper the interpretations of our data, as suggested.

I believe that the chemotherapy dosing schedules below standard are just dose reductions in the event of toxicity however; if so, the authors should just make that more clear in the text. It is clarified I believe in the figures S1, but the text is a little less clear and makes it seem like the dosing is variable for chemo, even in the absence of toxicity.

We have provided an explanation in the Methods “Study design and treatment” section on the dosing schedules and rationale for dose reductions that are based on toxicity.

Greater introduction and summary of expected toxicities of L-glutamine oral supplementation should be communicated.

We have expanded on the expected toxicities of oral L-glutamine based on phase III registrational trials in the Methods "Study design and treatment" section, as requested.

Greater explanation of rationale of the study is warranted. The mechanism of action of L-glutamine as a chemo supplement or enhancer was not well-described.

We acknowledge the need for greater clarity on the mechanism of action for the extraordinary response observed with L-glutamine and a chemo-supplement. There are clearly tumor cell-intrinsic and cell-extrinsic factors at play. This report primarily describes the host response to L-glutamine supplementation with standard chemotherapy. We were unable to address the biochemical impact on tumor cells here in the absence of tumor tissue access during/following treatment. However, we have a manuscript under review that details our understanding of the biochemistry, where high dose glutamine seemed to occupy the many redundant cell surface transporters (18) to deplete the tumor cells of other amino acids – resulting in tumor regression. However, the extensive work resulting in observed tumor regression in the preclinical models cannot be appropriately described here. Our results here focus on the role of stool bacterial metagenomics in patients and impact of glutamine supplementation on systemic metabolomics/inflammatory response. Specifically, we found that CCL11 and IL-33 to be elevated in the nonresponsive subjects (Extended Data Figure 8 [updated to Extended Data Figure 9 in final version]). An additional potential mechanism of L-glutamine's benefit may lie in its efficacy as an anti-cachexia therapy that warrants separate investigation. We have better described these findings in the discussion section to address this concern. However, due to the limited patient number, we cannot make conclusions as to a mechanism of action, rather these serve as newly identified biomarkers that require further validation in subsequent clinical studies.

In the methodology, it may be that the shotgun metagenomics and Dna extraction and shotgun sequencing describe similar library preps. Please clarify the difference.
The beta diversity comparisons of long and short survival, before and after l-glutamine are really confusing and the significance of the findings are not presented in a clear way making it difficult to assess.

We thank the reviewer for identifying this redundancy in the methodology. We have deleted the "DNA extraction and shotgun sequencing" in its entirety and renamed the section to "DNA extraction, shotgun sequencing, and bioinformatics analysis." Extended Data Fig 4b (updated to Extended Data Fig 5a in final version) demonstrated significantly reduced Shannon index (alpha diversity) in the gut microbiome for patients with short PFS. There was no significant difference in beta diversity across in response to glutamine treatment (Extended Data Fig 4c [Extended Data Fig 5b in final version]). We have included a description of these findings in the main text with reference to these figures for more clarity.

NMED-BC132311E-Z

Reviewer #2 (Remarks to the Author):

The investigators conduced a single-armed, phase 1, dose finding study evaluating administration of 1 week of oral L-glutamine in combination with gemcitabine and nab-paclitaxel, as a first line treatment for metastatic pancreatic cancer. There were no treatment related study discontinuations. The primary endpoint was to find the RPIID. Secondary endpoints included efficacy analyses and microbiome related studies. Patients were eligible if they had unresectable or advanced disease.
The optimal dose was found to be 30 g L glutamine per day. Most treatment related adverse events were related to the chemotherapy.

The authors highlight the originality in their responses. The paper is unique in that it seeks to characterize the anti-PDAC effects of glutamine, which has not been done.

The approach is rigorous, as are use of statistics.

The conclusions that the L-gln supplement is safe at the indicated dose is robust. The paper is well written and clear.

We thank the reviewer for their support of the study's originality and robustness of the statistical design and safety of L-Gln is appreciated. We provide a point-by-point response to the latest critiques and comments below.

I do think that the impact of this study remains unclear. There is no way to really know about the PK effects at the dosing, and therefore causality is a challenge to discern. The metabolites are variably effected by L-gln, and in some instances not at all. Glutamine levels are not affected, and while the authors explain that away, there is no clear substitute to demonstrate robust levels in the plasma and effects on tumors. It is possible that all of the effects are microbiome mediated, but that doesn't seem to be proven either. Some of the microbiome changes may just be random variation over time, attributable to diet. There are no controls showing what the variation should look like. Many of the changes are observed in responders vs. non-responders, but those may be attributable to response to the GA.

We thank the reviewer for raising this concern. A phase I dose-finding trial of this nature is not designed to have control arms, given the primary endpoint is establishing the RP2D of the study combination. We designed the preplanned correlatives such that each subject could serve as an internal control for multi-analyte profiling given the unique 1-week lead-in phase of L-glutamine alone allowing for biomarker assessments across pre- and post-glutamine treatment conditions. Plasma and stool were collected under fasted, timed morning collections to minimize heterogeneity. We understand the reviewer's main concern regarding the lack of effects on glutamine levels, especially since there is no clear substitute to demonstrate the effects of L-glutamine from a pharmacodynamic perspective. We have now included additional metabolomics data involving a primary comparison of amino acid levels in our patient blood samples that were not previously available at the time of the last revision. As shown in new Extended Data Figure 1 with corresponding addition in the main text, when expressed by actual concentration, we did observe a significant effect on glutamine levels when comparing baseline and post-glutamine treatment conditions, reinforcing the on-target effects of L-glutamine supplementation in the absence of gemcitabine/Abraxane (GA) chemotherapy. Notably, elevations in plasma glutamine represented the highest levels of all essential and non-essential amino acids measured in the cohort.

We previously demonstrated that glutamine-downstream metabolites were elevated following the glutamine lead-in time point inclusive of nucleotide metabolites and TCA cycle, occurring before the addition of GA chemotherapy and thus independent of GA chemotherapy as well (Extended Data Figure 2, 4, 8b). When putting this new data together with the downstream intermediary metabolite changes we previously showed, <u>we believe this supports that L-glutamine treatment does indeed impact L-glutamine levels and related intermediary pathways.</u>

Additionally, we had hoped to identify significant changes in the microbiome that could point to another potential pharmacodynamic effect of glutamine. Rather, as glutamine is arguably the most important nutrient utilized by gut bacteria, glutamine treatment demonstrated <u>bacterial metabolic reprogramming</u>. Bacterial phospholipid biosynthesis was elevated by glutamine treatment (Extended Data Figure 6c). More specifically, fatty acid oxidation was reduced by glutamine treatment in subjects with long OS, potentially contributing to improved gut health (Extended Data Figure 7). These novel findings, independent of GA chemotherapy, demonstrated glutamine administration could impact microbial metabolism.

We have emphasized in the revised manuscript that the pharmacodynamic effects of glutamine addition are likely multifactorial and include: 1) the impact on bacterial metabolism genes (not traditional microbiome composition due to the manner in which glutamine is used as a nutrient by gut bacteria), 2) impact on glutamine intermediary metabolism with demonstrated on-target effects on glutamine levels leading to uptake into intermediary pathways), and 3) impact on gut barrier integrity and malabsorption.

The OS and PFS are impressive, but very difficult to interpret in a single-phase study. The OR is roughly what we would expect with GA.

*We thank the reviewer for highlighting the limitations of a phase I dose-finding trial, which is not designed as head-to-head comparison of efficacy but rather to establish the safety and RP2D of the study combination. We do intend to advance this combination to a randomized controlled trial comparing glutamine + GA vs. GA + placebo, which would be the appropriate design for comparisons of therapeutic benefit. Within the context of this phase I trial, there was a secondary objective of signal finding for clinical benefit in which we did observe a preliminary but promising signal of efficacy. We have emphasized the need for validation of our phase I trial efficacy results in the main text. We argue that the overall response rate observed with our study combination of glutamine, gemcitabine/Abraxane (44%) was nearly double that (23%) of the registration trial for gemcitabine/Abraxane (*Von Hoff *NEJM* 2013 369:1691*). The CR rate of 12.5% is provocative, as a CR rate >1% has not been seen in recent phase III trials involving GA as an arm (Von Hoff NEJM* 2013 369:1691; *Wainberg Lancet* 2023 402:1272*).*

Reviewer #3 (Revision History)

NMED-BC132311D

Reviewer #3 (Remarks to the Author):

This is a single arm phase I study aimed at finding the RP2D of L-glutamine in combination with gemcitabine/abraxane in treatment naïve, metastatic PDAC patients. Oral L glutamine was found to be safe with standard doses of chemotherapy. With 16 enrolled patients, L-glutamine and GA combination achieved impressive clinical benefit. Exploratory studies further identified plasma metabolites and gut microbiome that are correlated with therapeutic response or patient survival. Overall, this is a well-designed single arm phase I trial with promising efficacy outcome. The use of glutamine for PDAC treatment is innovative. The statistical plan for primary and secondary endpoints are appropriate. Since it has been generally believed that glutamine is critical to support the growth of PDAC cells, the strong clinical benefit achieved with L-glutamine in combination with chemotherapy is somewhat unexpected. It provides strong rationale for phase II/III combinatory trials and additional preclinical/translational studies to further characterize the underlying mechanisms. There are few major concerns need to be further addressed/clarified.

We thank the reviewer for their comments and suggestions that strengthen the quality of our manuscript. Please find a point-by-point response to the reviewer below.

1. There lacks information on the general nutritional status of the patients before and after the treatment. It's unclear if any of the patients have underlying cachexia. Is there any correlation between therapeutic response/survival and changes in body weight, muscle or body mass?

We thank the reviewer for this suggestion. In a post-hoc analysis of this phase I trial cohort wherein the majority (81%) met consensus criteria for cachexia at study entry, we noted the following changes regarding body weight and BMI (of note, muscle and body mass composition assessments were not performed as part of the study design):

Baseline characteristics and evidence of weight stabilizing effects from L-glutamine and first-line gemcitabine and nab-paclitaxel

Characteristic (n=16)	Frequency (%) or mean ± SD
Median age in years (range)	71 (55-92)
Female	11/16 (69%)
Cachexia at study entry[a]	13/16 (81%)
Mean weight (kg) at study entry	73.1 (21.3)
Mean BMI (kg/m^2) at study entry	26.2 (6.4)
Weight stability[b]	8/16 (50%)
Median duration of treatment	8.6 months (95% CI 6-15)
Weight increase from baseline	8/16 (50%)
BMI increase from baseline	8/16 (50%)
Mean weight change (kg) from baseline	-0.39 (6.7)
Mean BMI change (kg/m^2) from baseline	-0.17 (2.7)

[a]Defined as >5% unexplained weight loss within 6 months prior to screening
[b]Defined as weight change less than 0.1 kg/baseline BMI-unit from cycle 1, day 1 of study treatment to end of treatment (median duration of treatment 8.6 months)
SD, standard deviation; CI, confidence interval

Specifically, we noted weight and BMI increases from baseline in 50% of the patients in this study. This corresponded to weight stability having been achieved in 50% of the subjects over a median duration of treatment of 8.6 months. Correlative analyses were performed for weight change, BMI change, and weight stability according to efficacy outcomes as shown below and found as Extended Data Tables 6-8 in the revised submission:

Association between weight stability and efficacy end points (n=16).

	**p-value from Fisher's exact test**	**p-value from exact log-rank test**	
	**Best overall response** CR+PR vs. SD+PD	**OS** dead vs. alive	**PFS** PD vs. PF
**Weight stability**[a] Yes vs. No	>0.999	0.682	0.742

[a]Defined as weight change less than 0.1 kg/baseline BMI-unit from cycle 1, day 1 of study treatment to end of treatment (median duration of treatment 8.6 months)
CR, complete response; PR, partial response; SD, stable disease; PD, progressive disease; OS, overall survival; PFS, progression-free survival; PF, progression free

Association between weight stability and tumor shrinkage; weight/BMI change and efficacy endpoints (n=16)

	**Mean ± standard deviation**		**p-value (Mann-Whitney U test)**	**p-value (Cox regression)**
**Tumor shrinkage**[a], %	Weight stability[b] **Yes** n=8	Weight stability **No** n=8	0.958	.
	-36.09 ± 45.47	-32.04 ± 31.13		
**Weight change**, kg	Best overall response **CR+PR** n=7	Best overall response **SD+PD** n=9	0.289	.
	-2.39 ± 7.11	1.17± 6.77		
	OS **dead** n=10	OS **alive** n=6	0.871	0.790
	-0.97 ± 7.74	0.58 ± 5.86		
	PFS **PD** n=14	PFS **PF** n=2	0.382	0.515
	-1.06 ± 6.88	4.30 ± 7.35		
**BMI change**	Best overall response **CR+PR** n=7	Best overall response **SD+PD** n=9	0.091	.
	-1.30 ± 2.57	0.72± 2.86		
	OS **dead** n=10	OS **alive** n=6	0.492	0.966
	-0.52 ± 3.22	0.42 ± 2.20		
	PFS **PD** n=14	PFS **PF** n=2	0.600	0.470
	-0.36 ± 2.89	1.21 ± 2.80		

[a]Tumor shrinkage defined as any reduction in tumor target lesions from baseline
[b]Defined as weight change less than 0.1 kg/baseline BMI-unit from cycle 1, day 1 of study treatment to end of treatment (median duration of treatment 8.6 months)
CR, complete response; PR, partial response; SD, stable disease; PD, progressive disease; OS, overall survival; OS, overall survival; PF, progression free; BMI, body mass index; PFS, progression-free survival

Overall, there were no statistically significant associations with efficacy outcomes and nutritional parameters, which could be explained by the smaller sample size of our cohort. However, we believe the reviewer raises an important question given the encouraging weight stability achieved (50%) across this predominantly cachectic cohort of advanced PDAC patients. The weight stability observed over a meaningful duration of treatment of >8 months with L-glutamine and gemcitabine/nab-paclitaxel is hypothesis generating, particularly in this disease subset which is highly aggressive and where cachexia contributes greatly to morbidity and mortality. We note multiple prior studies of exogenous L-glutamine's potential as an anti-cachexia strategy across preclinical cancer models (PMID: 1975037; 27529497; 11979506; 27635657; 28345452). As such, we strongly believe that separate and further investigation of L-glutamine's anti-cachexia potential is warranted.

2. There are some minor issues with the classification of metabolism pathways. It's unclear why myo-inositol and D-mannose are part of nucleotide metabolism pathway. Similarly, the inclusion of nicotinic acid in fat/cholesterol pathway and the inclusion of lactate in TCA.

We agree the two are not directly linked to nucleotide biosynthesis. However, myo-inositol is part of the pentose phosphate pathway (PPP) and D-mannose is part of the UDP-GlcNAc pathway (Extended Data Fig 1 [updated to Extended Data Fig 2 in final version]). PPP provides the ribose-5-phosphate (R5P) backbone for nucleotide synthesis. UDP-GlcNAc is derived from uridine diphosphate, as a nucleotide sugar. Incidentally, the global elevation in the two metabolites by L-glutamine treatment was in line with the observed nucleotide biosynthesis (uridine, GlcNAc-phosphate, 5-Deoxy-5(methylthio)adenosine) elevation significantly associated with greater OS (Extended Data Fig 3 [Extended Data Fig 4 in final version]). We have highlighted this connection in the results section of the revised submission.

3. It is inaccurate to claim that "amino acid biosynthesis……. were elevated with L-glutamine treatment" (line 117-118) although some amino acid metabolism intermediates are elevated.

We agree. In fact, only a couple were identified to be elevated with glutamine treatment: N-carbamoyl aspartic acid and 3-methylglutamic acid (Extended Data Fig 1 [updated to Extended Data Fig 2 in final version]). However, stool metabolite analysis in the revised submission (Extended Data Fig 7 [Extended Data Fig 8 in final version]), demonstrated seven amino acids were significantly elevated in the non-responsive subjects, compared to the responsive subjects following glutamine administration (not found at baseline for the same patients). This further supported that malabsorption in these non-responsive subjects would likely lead to the observed weight loss and reduced chemotherapy responsivity (Extended Data Table 6-8). Circulating levels of LPS was significantly suppressed by glutamine administration (for both responders and non-responders; $P < 0.0001$, Extended Data Fig 8a [Extended Data Fig 9a in final version]) to suggest the restoration of barrier function by glutamine supplementation. Incidentally, gram negative bacterial expansion is observed to negatively impact neutral amino acid absorption. We have added this to the Results/Discussion section.

NMED-BC132311E-Z

Reviewer #3 (Remarks to the Author):

I agree with the authors' rebuttal and I have no additional concern.

We thank the reviewer for approving our revised manuscript.

Reviewer #4 (Revision History)

NMED-BC132311D

Reviewer #4 (Remarks to the Author):

Gong and colleagues provide novel insights into the increase treatment efficacy of L-glutamine supplementation in combination with gemcitabine/nab-paclitaxel in pancreatic ductal adenocarcinoma. The authors demonstrated that L-glutamine administration was well tolerated and resulted in improved patient outcomes, shown by OS and PFS. The study also demonstrated that L-glutamine supplementation influenced the levels of metabolites (amino acids, nucleotides) present in the bloodstream. However, no alterations were observed in the concentrations of glutamine and glutamate in the blood. Interestingly, the authors demonstrated a reduction in the prevalence of gram-negative bacteria in patients who exhibited either a complete or partial response after glutamine administration. In addition, LPS level were reduced in patients' stool after L-glutamine co-treatment.

Overall, this is a very well-written and presented manuscript describing an interesting and highly relevant mechanism of cancer cell metabolism. The authors present their findings clearly and describe them accurately within the text. I really enjoyed reading this paper. A real strength of this study is the metabolic intervention in patients. Nevertheless, the current set of data raise some open questions, which, at this stage, maintain the study's descriptive nature.

We appreciate the reviewer's time and effort spent to help us strengthen our manuscript. Please find a point-by-point response to the reviewer below.

1. The authors should provide data for the same metabolites in Figure S2 (Metabolite changes following oral L-glutamine lead-in period) and Figure S3 (Metabolite changes following the introduction of the first dose of gemcitabine and nab-paclitaxel). This will ensure the comparability of the two important data sets.

We agree with the reviewer regarding showing the same metabolites in the baseline and glutamine lead-in timepoints. As there were over 30 metabolites involved, we opted to add Extended Data Table 5 to convey the data better in the revised manuscript.

2. In line with the first point, the authors should try to provide the most significant metabolites of each metabolite's classes, right now this is not presented in the current manuscript. For example, lactate and pyruvate, which were presented in Figure S2, are absent from Figure S3. As two of the most abundant metabolites, the authors should provide this information for Figure S3 as well.

We added Extended Data Table 5 to address the requested information in the revised manuscript.

3. A further crucial aspect is the presentation of the metabolites with concentration (quantification), as opposed to the current version of the manuscript, which presents them with area under the curve (AUC). For a better insight the author should provide among others most importantly glutamine and glutamate quantification in the metabolic analysis of the blood samples.

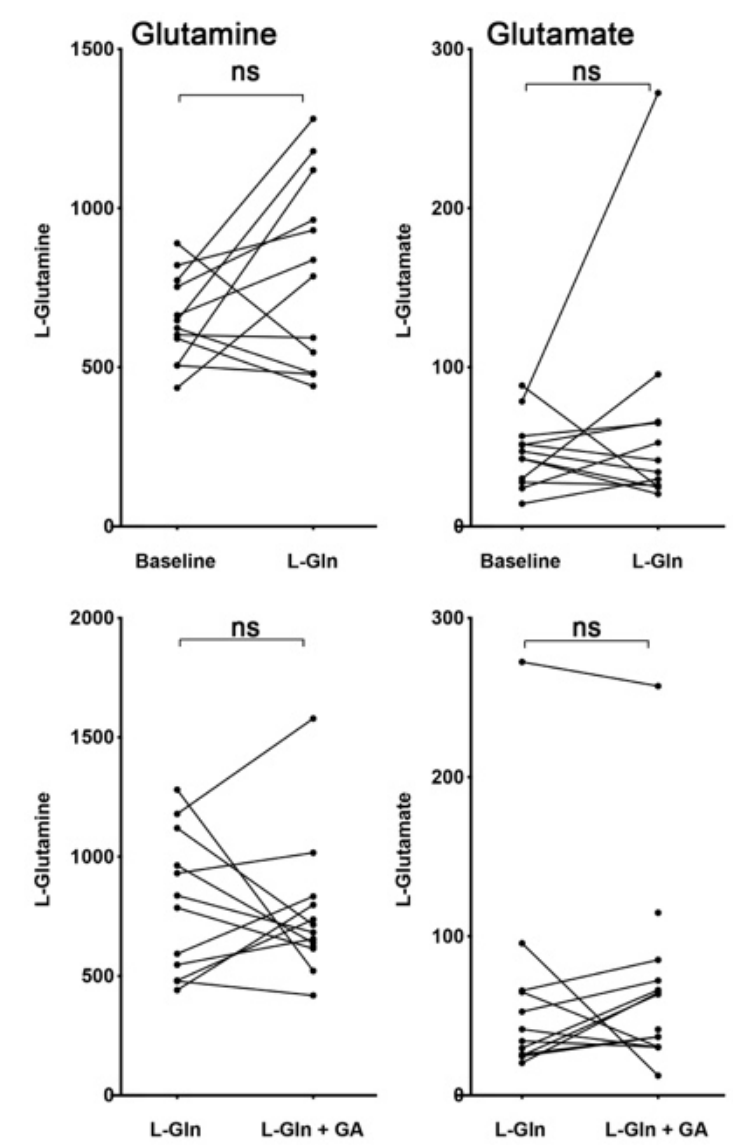

We initially quantitated the glutamine and glutamate content in the plasma from subjects at baseline and following glutamine treatment (right Figure) using relative abundance measured by mass spectrometry; specific quantitation similarly yielded no statistical significance. Of note, we provided a subsequent analysis involving a primary comparison of amino acid levels in our patient blood samples that were not previously available at the time of this revision. We did identify a significant elevation in plasma glutamine levels from L-glutamine administration. This is described in response to Reviewer #2 above and was not available to this reviewer during this revision round.

4. Can the authors provide additional PCA, PLS-DA analysis of the metabolites in patients following oral L-glutamine lead-in period and following the introduction of the first dose of gemcitabine and nab-paclitaxel.

We have added a new panel to Extended Data Table 5 to illustrate the metabolites in the 3 time points as requested. Additional PCA analysis of the metabolites across the 3 time points has been incorporated into Extended Data Figure 1 (updated to Extended Data Figure 2 in final version).

5. The authors should provide, if possible, a metabolic analysis of the stool samples in order to allow for comparison with the changes observed in the patients' blood.

We have added new metabolic data from the stool specimens in Extended Data Fig. 7 (updated to Extended Data Fig. 8 in final version). These data supported the role of glutamine administration impacting nutrient absorption with the identification of significant elevation in seven amino acids of the less responsive subjects. Malabsorption due to dysregulation of the microbiome would support the absence of weight stability observed in the responders of glutamine administration (Extended Data Table 6-8; also discussed above for Reviewer #3).

6. Did the patients receive any antibiotic therapy in the weeks and months before the L-glutamine therapy?

A second review of available clinical data shows that 3 subjects received an antibiotic course of >7 days 1 month before study start, while 1 subject received a 7-day antibiotic course 2 weeks before study start. There was no clear pattern in the among the four subjects in terms of responsiveness.

7. The authors should expand the discussion of why they did not observe any changes in glutamine/glutamate levels upon L-glutamine administration.

Regarding our initial finding that glutamine/glutamine levels did not change with L-glutamine administration, we cited one possibility based on recent prospective clinical trial data from healthy subjects and subjects with sickle cell anemia receiving up to 0.3 g/kg of oral L-glutamine twice daily (consistent with our maximum doses of oral L-glutamine) that showed rapid absorption of L-glutamine from the bloodstream after administration. For example, the mean half-life of plasma L-glutamine was 1 hour, mean peak plasma concentrations of L-glutamine occurred at 1.2 hours, and L-glutamine plasma concentrations returned to baseline within a few hours across all doses of oral L-glutamine (PMID: 38401036). However, we subsequently presented further analysis involving a primary comparison of amino acid levels in our patient blood samples that were not

available at the time of this revision. We were able to demonstrate that L-glutamine therapy significantly elevated plasma glutamine concentrations (please see response to Reviewer #2 NMED-BC132311E-Z above where this new data was presented).

NMED-BC132311E-Z

Reviewer #4 (Remarks to the Author):

This is a revised version of the previously submitted manuscript from Gong and colleagues. The authors have performed a thorough revision of the manuscript and included extended series of novel observations and data.

The authors have fully addressed all of my comments, and therefore I recommend this work to be accepted for publication.

We thank the reviewer for approving our revised manuscript.

<u>Reviewer #5 (Revision History)</u>

NMED-BC132311D

Reviewer #5 (Remarks to the Author):

This manuscript presents interesting findings on the use of L-glutamine in combination with standard chemotherapy for metastatic pancreatic ductal adenocarcinoma (PDAC). Their observations seem to be very significant as L-glutamine administration in combination with GA nearly doubled the ORR and tripled the OS to historical GA alone. However, several issues temper this reviewer's enthusiasm for the study's conclusions.

We thank the reviewer for taking the time to help us to improve our paper. A point-by-point response is provided below.

Major:
1, Single-arm design: Without a control group, it is challenging to attribute the observed effects solely to L-glutamine. External factors, patient variability, or even the placebo effect could influence outcomes.
This is particularly true for patient variability: the cohort includes patients with varied metastatic sites and different histories of prior surgeries, potentially impacting outcomes and confounding results.

We agree with the reviewer that the single-arm design and smaller sample size of our clinical trial limits definitive conclusions to the efficacy our study treatment. This phase I study was designed primarily to determine the safety and RP2D of the combination of L-glutamine, gemcitabine, and nab-paclitaxel as a first-line treatment in metastatic PDAC patients. The efficacy outcomes were secondary endpoints but essential to determine preliminary signals of efficacy warranting advancement to a larger, prospective clinical trial. Selection bias and heterogeneity of study subjects in a phase I trial of this nature are certainly limitations as well. We do note that the demographics and baseline characteristics of our cohort appear reflective of what we see in the clinic. For example, the median age being 71, the majority (81%) meeting consensus criteria for cachexia at study entry, the majority (75%) having de novo unresectable or metastatic disease, and the sites of metastases with liver and lung being most common at presentation (68%). We have also provided additional data on post-progression therapies in our cohort at the request of another reviewer (see Extended Data Table 4 on post-progression therapies included in the revised manuscript). We believe this data further reinforces the benefit driven by first-line study treatment in our cohort, rather than that driven by post-progression therapies. We have also tempered our conclusions to ensure that the take-away message is that validation of our findings in a randomized controlled trial would be the logical next step. We do strongly believe though that although preliminary, these efficacy signals are promising enough to justify a randomized controlled trial, particularly given the high unmet need for improvements in outcomes for patients with metastatic PDAC. This is more relevant given the exquisite safety and non-overlapping toxicity observed with L-glutamine to standard chemotherapy and the availability of a pharmaceutical grade, FDA-approved compound that is readily available for translation into late-phase clinical trials.

2, The sample size is small: With only 16 evaluable subjects, the study's findings may lack sufficient statistical power, and the results might not generalize well to a broader PDAC population. Specifically, while the study concludes that the RP2D (Recommended Phase 2 Dose) was safe, DLT (Dose-Limiting Toxicity) data might be underpowered due to the small sample size, which could miss rare or delayed toxicities.

We thank the reviewer for raising this concern. Cancer phase I trials are typically designed to estimate the maximum tolerated dose (MTD) or recommended phase 2 dose (RP2D). As such, they are not designed to test a specific hypothesis or powered to favor a particular alternative hypothesis. The performance of such designs is evaluated using operating characteristics under different scenarios for the location of the true MTD. In our trial, the operating characteristics were reasonable for a sample size of 16; the probabilities of correctly

selecting the MTD are 0.82, 0.57, and 0.613 when the true MTD is the lowest dose, middle dose, and last dose level, respectively. The MTD is usually defined as the dose with a pre-specified target probability of DLT after 1 or 2 cycles of therapy. Hence, delayed toxicities are not considered when the algorithm recommends dose escalation or de-escalation. These types of toxicities are monitored in larger phase II/III trials.

3, The investigators compared survival outcomes to historical GA-treated cohorts, but differences in baseline characteristics and treatment environments might confound the comparison.

A comparison to historical GA-treated cohorts is a valid limitation, but the goal was to characterize any preliminary signals of efficacy to the study combination as a secondary endpoint. The comparison to historical GA outcomes was presented to underscore these preliminary efficacy signals albeit in a single-arm, phase I trial that is designed without a control arm. The primary endpoint was to determine the RP2D instead, which we demonstrated for maximum doses of L-glutamine with maximum standard doses of GA. As described above, heterogeneity in patient characteristics is not unexpected in a phase I trial of this nature, but we reassuringly found that baseline characteristics of our cohort were reflective of what we routinely see in the oncology clinic (i.e., older population, predominantly cachectic, the majority with metastatic disease at presentation, and liver and lung metastases being the most common sites of disease). We have also provided more data on post-progression therapies in our cohort at the request of another reviewer (see Extended Data Table 4 on post-progression therapies included in the revised manuscript). As evidenced, the benefits to second-, third-, and fourth-line therapies following progression to study treatment was short lived with a median PFS < 2 months. This is reassuring that the survival benefits were more likely driven by our first-line study combination rather than post-progression therapies. Several patients experienced median PFS benefits of well over 1 year to study treatment that were likely underappreciated by our median PFS in the overall cohort as well. We have nonetheless tempered our conclusions that although our results are promising, they warrant further validation in a larger randomized controlled trial.

4, While changes in the microbiome and metabolome are noted, the study doesn't clearly establish causation or mechanistic links between these shifts and patient outcomes.

We have added stool metabolomic data in the revised submission (Extended Data Fig 7 [updated to Extended Data Fig 8 in final version]), and added a discussion of the findings to address this concern:

We found that a general induction of fatty acid oxidation with progressive disease and elevated fatty acid biosynthesis pathways with patients showing responsivity at baseline in patients receiving glutamine administration with standard-of-care gemcitabine and nab-paclitaxel. With respect to the gram-negative bacteria enriched in the less responsive subjects, like Klebsiella, Vescimonas, and Dysosmobacter (Extended Data Fig 6), all seem to upregulate mechanisms of fatty acid oxidation with elevated enzyme, enoyl CoA hydratase (Extended Data Fig 6 [Extended Data Fig 7 in final version]), required for processing fatty acyl-CoA intermediates derived from ethanolamine breakdown into short chain fatty acids. As discussed above (see response to Reviewer #3), amino acid enrichment in the stool of the non-responsive subjects was suggestive of malabsorption as it was accompanied by greater levels of amino acids in the stool of the non-responsive subjects. The negative impact on intestinal amino acid transporters by the specific bacteria found to be elevated could contribute to the observed nutrient malabsorption and weight loss in the non-responsive subjects.

5, Gemcitabine and nab-paclitaxel might independently affect the microbiome, metabolome, and inflammatory markers, complicating the attribution of these changes solely to L-glutamine.

As this was a single arm study, only the role of glutamine and the combination with chemotherapy was assessable. While there are previous reports on gemcitabine induced microbiome changes (PMID: 36734518, PMID: 28912244), we are not aware of any in the context of nab-paclitaxel. Interestingly, the bacteria enriched

in the non-responsive subjects did not change appreciably with glutamine administration (Extended Data Fig 4 [updated to Extended Data Fig 6 in final version]). Glutamine administration, however, seemed to significantly limited gut permeability of LPS (Extended Data Fig 8 [Extended Data Fig 9 in final version]). We addressed this limitation in the revised discussion section.

Minor:
1, Introduction: "The disruption of L-glutamine uptake and metabolism are reported to sensitize PDAC cells..." "are" should be "is," as "disruption" is singular.

We have made this correction in the Introduction. We have extensively revised the manuscript.

2, Results: Last sentence: “reinforces” should be “reinforce”.

We have made this correction in the last sentence.

3、Lines 127-129, The description of microbial diversity analysis does not match the figures: Fig S5b & c show alpha diversity (Shannon index) rather than beta diversity. Additionally, the impact of L-glutamine on gut microbiota is not clearly explained. Did the L-glutamine lead-in alter specific microbial changes or ecological niches to facilitate GA activity? In addition, the significance markers in Fig S5b & c do not clearly indicate which groups are significantly different from each other.

We have made the correction, where the alpha diversity is depicted in the revised submission Extended Data Fig 4a (updated to Extended Data Fig 5a in final version). The pathway analysis in Extended Data Fig. 6b (Extended Data Fig. 6c in final version) suggests alteration in bacterial gene content related to polyamine metabolism, so we could speculate that bacteria capable of metabolizing glutamine were favored. Since stool sampling was performed before GA administration, the microbial changes reported in the manuscript did not reflect interaction of glutamine with GA in the gut. Extended Data Fig 4b (Extended Data Fig 5a in final version] demonstrated significantly reduced Shannon index (alpha diversity) in the gut microbiome for patients with short PFS. There was no significant difference in beta diversity (Extended Data Fig 4c [Extended Data Fig 5b in final version]). We have included a description of these findings in the main text with reference to these figures for more clarity. There were no other apparent changes in alpha or beta bacterial diversity in response to glutamine.

4、It is strongly recommended to indicate the sample size for each group in the figures, such as in Fig S5b & c and Fig S6. More importantly, differential analysis requires multiple testing correction to avoid false positives.

We thank the reviewer for this suggestion. We have addressed this in the respective figure legends.

5、In Fig S6a, the names of the first two taxa in the top row are the same, but their abundance compositions differ—could this be a typo? Additionally, why are the colors in the first column different from those in the bottom two columns?

We understand the confusion in Extended Data Fig 5 [updated to Extended Data Fig 6 in final version]. In the revised submission, we have divided panel 5A into two separate panels to indicate differential microbiota abundance distinguishing short and long PFS (Extended Data Fig 5a [Extended Data Fig 6a in final version]) and short and long OS (Extended Data Fig 5b [Extended Data Fig 6b in final version]). The differential coloring of the bars is associated with changes impacting OS and PFS.

6、 There should be a clear and logical framework explaining the rationale behind the microbial analyses. Specifically, it should clarify when comparing long OS and short OS is relevant, and when comparisons between L-glutamine baseline and after treatment are necessary.

The rationale for the examination of the microbiome stems from the well described gut-supportive role and reduction of gut permeability by the administration of glutamine. Bacteria like, Lactobacillus and Bifidobacterium enhance tight junction function of intestinal epithelia. Similarly, Akkermansia muciniphila strengthens the mucus barrier and butyrate producing Faecalibacterium prausnitzii fuels colonocytes. While dysbiosis can be associated with gram negative bacteria that releases LPS. The administration of glutamine universally suppressed circulatory LPS (Extended Data Fig 8a [updated to Extended Data Fig 9a in final version]). However, those that were responsive to glutamine treatment with chemotherapy had significantly reduced CCL11 and IL-15 compared to the less responsive subjects (Extended Data Fig 8b [Extended Data Fig 9b in final version]).

Importantly, stool microbiota differed among the responders and non-responder subjects. For example, Klebsiella, Dysosmobacter, and Veilonella were significantly elevated in patients with a short overall survival. These bacteria are reported to inhibit intestinal transporter expression, highlighted by stool metabolic analysis, the non-responders had significant enrichment of 7 amino acids to further the evidence for dysbiosis. Since this was a single arm study where all subjects received glutamine treatment, we could only compare metagenomics at baseline and post-glutamine treatment (see explanation for Reviewer #3).

7、 Is there redundant information in the Methods section between Lines 350-357 and the DNA extraction and shotgun sequencing section (Lines 368-376)?

We thank the reviewer for identifying this redundancy in the methodology. We have deleted the "DNA extraction and shotgun sequencing" in its entirety and renamed the section to "DNA extraction, shotgun sequencing, and bioinformatics analysis."

8, Could you provide an explanation as to why the initial pre-specified value α is set at 0.4, given that α is typically set between 0.2-0.33? Additionally, please explain the rationale behind the three scenarios designed in Table 2.

It is true that the starting value of the feasibility bound α is between 0.2 and 0.3. However, this is the case when the starting dose is the minimum dose level available in the trial. In this trial, there are 5 dose levels ordered as -2, -1, 0, 1 and 2. The starting dose level for this trial is dose level 0. To maintain the coherence principle of EWOC (or CRM) designs, if there is no DLT at dose level 0, the next patient should not be given a lower dose, -1 or -2. Using the prior distribution of the MTD, if we chose an α between 0.2 and 0.3, then the next dose level would be -1. Therefore, a starting feasibility bound of 0.4 was selected for the algorithm to recommend dose level 1 for the next patient if the first patient treated at dose level 0 has no DLT. The resulting operating characteristics in the protocol achieve good performance with respect to percent selection and patients treated at the MTD.

NMED-BC132311E-Z

Reviewer #5 (Remarks to the Author):

1. The authors have not yet adequately addressed key issues related to microbiome data analysis in Extended Data Fig. 4. Notably, there appears to be a persistent confusion between the concepts of alpha and beta diversity. The Shannon index, which measures within-sample microbial diversity by accounting for both

richness and evenness, is a well-established metric of alpha diversity—as is the Simpson index, which was referenced in the original manuscript.

We understand the reviewer's confusion as these issues were largely due to retained text from a prior version of the figure, for which we have corrected in both the main text, methods, and figures of the microbiome data analysis. We agree the microbiome effects should be clarified. We contend that there are significant changes in Shannon index in patients with short PFS. However, unlike many traditional microbiome studies we did not find any appreciable change in the overall microbiome populations in response to treatment. Given that glutamine is a critical nutrient utilized by gut bacteria, we next sought to investigate whether its impact is likely after glutamine uptake on bacterial metabolism and metabolism genes. Contributing to the novelty of this study, there were marked changes in microbiome metabolism in response to glutamine, such as gluconeogenesis, polyamine and phospholipid biosynthesis, observed independent of the impact of chemotherapy with GA. Involvement of bacterial fatty acid metabolism and gluconeogenesis pathways were also significantly associated with efficacy outcomes to our study combination as well. We have expanded on the specific impact glutamine had on bacterial metabolism in the main text.

Moreover, the figure panels and accompanying legends remain inconsistent and require revision:
• Extended Data Fig. 4a: The NMDS (non-metric multidimensional scaling) plot lacks essential information on the distance metric used (e.g., Bray-Curtis, Jaccard), which is necessary for proper interpretation of beta diversity patterns.

We have now indicated in the figure legend that Bray-Curtis dissimilarity was used. The NMDS plot has also been relabeled as Extended Data Fig 4b (updated to Extended Data Fig 5b in final version).

• Extended Data Fig. 4b and 4c: These panels clearly present alpha diversity data (Shannon index), yet the authors incorrectly refer to a reduction in "beta diversity" in patients with short progression-free survival (PFS) in their response. This mischaracterization must be corrected to avoid conceptual confusion.

The figure legend has been clarified to indicate that these figure panels are showing results of alpha diversity analysis (Shannon index, which reflects richness and evenness). This figure has also been relabeled as Extended Data Fig 4a (updated to Extended Data Fig 5a in final version).

• Group comparison ambiguity: The authors state that a "significantly reduced Shannon index" was observed, but do not clarify the groups being compared—whether this refers to differences between L-Gln^{+} and L-Gln^{-} arms, responders vs. non-responders, or across timepoints. Precise labeling of comparison groups (e.g., baseline vs. post-treatment, stratified by clinical outcomes) is essential to ensure interpretability.

We have now clarified in the main text that significantly reduced Shannon index was seen in patients with short PFS after adjusting for timepoint and considering subject as a random effect. The labeling of the timepoints in Extended Data Fig. 4b and 4c (updated to Extended Data Fig 5a in final version) was also further explained in the figure legend. A new plot has been added that stratifies alpha diversity (Shannon index) by baseline vs. post-treatment. This new plot along with the prior plots stratifying by clinical outcome has been relabeled as Extended Data Fig 4a (Extended Data Fig 5a in final version).

• Inconsistency in analytical methods: The Methods section indicates that beta diversity was assessed using Bray-Curtis dissimilarity and visualized via Principal Coordinates Analysis (PCoA), whereas Extended Data Fig. 4a employs NMDS for dimensionality reduction. This discrepancy between the stated and applied methods compromises reproducibility and should be reconciled.

The Methods section has been corrected to indicate that NMDS was used for visualization (now referring to the newly relabeled Extended Data Fig 4b [updated to Extended Data Fig 5b in final version]).

We strongly recommend revising Extended Data Fig. 4, its legends, and the relevant manuscript text (Lines 142–145) to ensure consistent and accurate use of diversity terminology, analytical methodology, and figure labeling.

Extended Data Fig. 4 (updated to Extended Data Fig 5 in final version), its legend, and the associated manuscript text have been revised to provide further clarity.

2. While the authors report the use of MaAsLin2 with "linear mixed-effects models to confirm microbial differential signatures," essential methodological details are missing. To ensure transparency and reproducibility, the model specification should be clearly stated, including the fixed effects (e.g., treatment group, clinical outcomes such as PFS and OS) and random effects (e.g., patient ID, batch effects) incorporated in the analysis.

We have expanded the figures legends for Extended Data Fig. 5 and Fig. 6 (updated to Extended Data Fig 6 and 7 in final version) as well as the Methods section to indicate the model specification for MaAsLin2 analyses, including fixed effects and random effects.

3. The inclusion of post-progression treatment data (Extended Data Table 4) supports the interpretation that the observed survival benefit is primarily associated with first-line therapy rather than subsequent interventions, thus addressing prior concerns about potential confounding. However, to further assess the robustness of this conclusion, a direct comparison of baseline characteristics—such as age, performance status, and metastatic burden—between the study cohort and historical controls is necessary. The statement that the cohort is "consistent with clinically representative populations" is insufficient to rule out selection bias. We recommend incorporating sensitivity analyses (e.g., survival comparisons adjusted for baseline imbalances) into the Discussion or, alternatively, providing explicit details about the historical controls—such as their institutional origin and whether they were drawn from a contemporaneous time period.

We thank the reviewer for raising this concern. The efficacy outcomes were stated as a comparison to the historical efficacy outcomes of the phase III registrational trial of gemcitabine/Abraxane (GA) by Von Hoff et al. (*NEJM* 2013 369:1691)*. A direct comparison is beyond the scope of a dose-finding phase I trial. We do intend to conduct a placebo-controlled, randomized clinical trial of our study combination glutamine + GA vs. placebo + GA, which would be the more recognized and appropriate design for this comparison as the next step.*

4. It should be noted, however, that the authors have not provided a version of the manuscript with tracked changes. As a result, it is difficult to assess the specific modifications made to the main text. I recommend that the authors include a tracked-changes version in their revised submission to facilitate further review.

We are unclear why tracked changes were not seen by the Reviewer as both tracked and cleaned versions were uploaded to the submission portal. An updated tracked and clean version of the final version of the manuscript in PDF has now been provided.

Reviewer #1 (Remarks to the Author):

The authors performed a phase 1 dose finding study identifying the maximum tolerated dose of L-glutamine in combination with gemcitabine and abraxane for patients with advanced pancreatic cancer. They discovered that the amino acid is well-tolerated with chemotherapy and observed correlative signals indicating activity in the form of changed circulating plasma and stool levels of metabolites and other markers. This is a novel study and very relevant. The secondary endpoints of survival were impressive and promising compared to historical controls. I commend the trial investigators on the hypothesis, study design and execution of the study. The statistics and analysis are sound.

I have several questions and comments which I consider to be mostly minor.

We thank the reviewer for taking the time to review our manuscript. We provide a point-by-point response to critiques and comments below.

Major

1) Were trial participants metastatic only or locally advanced- this is confusing. I thought it was only metastatic based on the results but then 1A implies locally advanced was included. This was also mentioned in the methods. If so, how many were locally advanced? And the expected survival is different in this cohort, such that the reference used for historical controls should be changed and the interpretation of the secondary endpoint may need to be adjusted.

We appreciate the reviewer for raising this concern regarding stage at enrollment and how this may affect expected survival. The study protocol allowed enrollment of patients with both locally advanced, unresectable or metastatic PDAC so long as they were treatment naïve. On study entry, 15/16 patients were stage IV or metastatic and only 1 patient was locally advanced, unresectable. This lone patient with locally advanced, unresectable disease was a primary progressor on first-line L-glutamine and GA (i.e., PD was best overall response) and experienced an OS of 7.2 months, which is below the median 8.5 months of GA in the historical reference. As a result, we are confident that our study cohort is overall representative of a metastatic cohort without substantial impact by the 1 patient with locally advanced, unresectable disease. It could be argued that inclusion of this 1 patient instead brought down the ORR and survival outcomes of this cohort. We have revised the main text, figure legends, and methods section accordingly to clarify that an advanced PDAC cohort of predominantly metastatic patients with 1 locally advanced, unresectable patient were enrolled.

Minor

1) An editing suggestion: "Broadly, those patients having longer survival to glutamine based therapy had reduced abundance of gram-negative bacteria at baseline and after L glutamine treatment" might want to be edited to "....experiencing longer survival on glutamine based therapy ..."

We have revised that sentence according to the reviewer's suggestion.

2) Is 7 days enough to see changes in microbiome? What if you did a 2 week or 3 week lead-in? Would that be ethical to delay chemo by that long and if so, would that have made a difference in your alpha diversity effects?

The effect of longer L-glutamine lead-in periods on the microbiome and alpha diversity remains unknown to us and we can only speculate. However, we do agree with the reviewer that delaying chemotherapy any further beyond the 1-week L-glutamine lead-in phase may present ethical challenges, particularly in the context that prescreening and screening adds additional time in between diagnosis and initiation of standard-of-care chemotherapy in a fairly sick patient population.

3) An editing suggestion: I know what your saying here but you could write a better sentence: "However, with higher gram-negative bacteria content in the gut of short OS patients under basal conditions, glutamine being the preferred nitrogen source resulted in greater respiration." Along those lines, please be specific as to what you mean by respiration and how you determined that.

We thank the reviewer for this editing suggestion. Both gram-positive and gram-negative bacteria utilize glutamine as a respiratory energy source, however gram-negative bacteria might preferentially use glutamine to support fatty acid oxidation rather than anaplerotic metabolism, being more adaptive of the two bacterial types. Gram-positive bacteria generally rely on short chain fatty acids and use the TCA cycle primarily for biosynthesis rather than energy. Gram-positive bacteria rely more on fermentation or glycolysis and have less TCA flux in many species. The higher gram-negative bacterial populations in the short OS subjects accordingly had elevation in enoyl CoA activity in their stool specimens, suggestive of fatty acid oxidation. A revision of this statement, to this effect, has now been included in the main text.

4) It is confusing when you write about microbiome changes 'in the presence of GA' because only blood was taken after GA. There were no stool samples taken at after the first dose of GA.

The reviewer is correct, no stool samples were taken after the first dose of GA. The statement "in the presence of GA" is made in reference to circulating LPS levels that were measured in blood samples taken before and after GA (Extended Data Fig. 9a).

5) Anything special about GA? Why not FOLFIRINOX? Why did you choose GA in this trial?

We thank the reviewer for raising this question. The rationale for GA was based on PDAC preclinical studies whereby L-glutamine supplementation improved gemcitabine-based chemotherapy efficacy through amino acid depletion of the pancreatic cancer cells (currently a preclinical manuscript under review in another journal). The biochemistry of glutamine transporter occupation of tumor cells by high dose glutamine resulting in amino acid depletion was deemed to be outside the scope of this brief report; but we have included a key figure shown below.

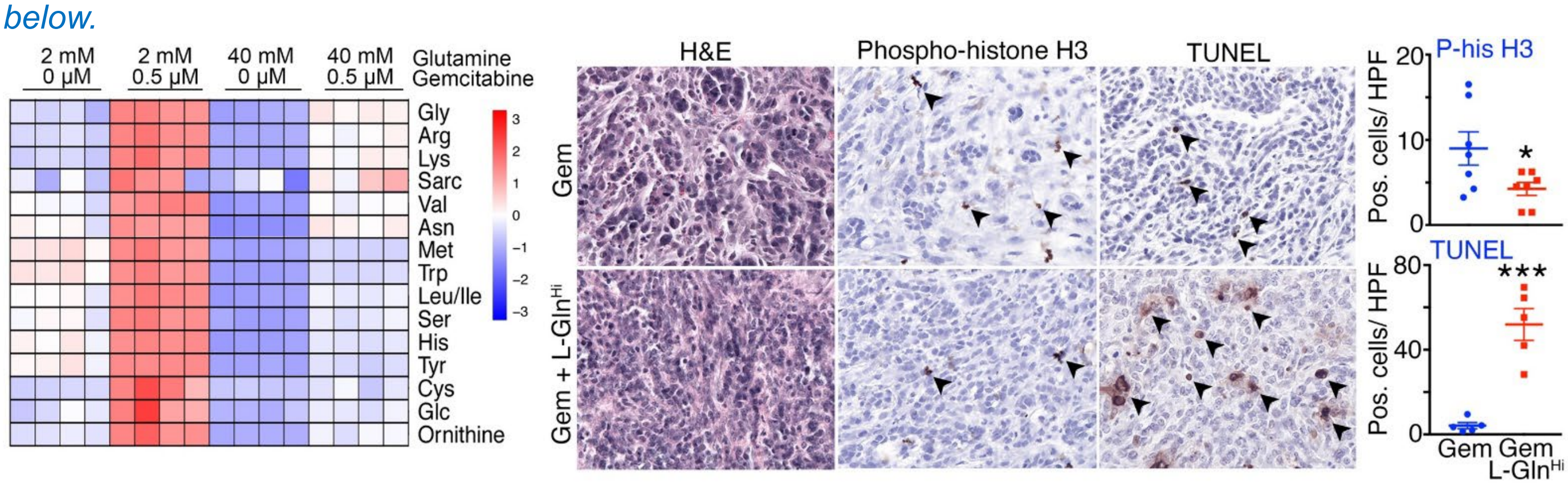

Glutamine supplementation to gemcitabine treatment results in amino acid depletion and tumor regression. Heatmap shows an enrichment of amino acids in cultured PDAC cells (MIAPaCa2) surviving gemcitabine treatment that is restored to near control levels when high glutamine levels are administered in combination (Left). Syngeneic mice orthotopically grafted with PDAC cells (KPC961) were treated with gemcitabine alone or in combination with glutamine. Combination therapy resulted in a significant increase in apoptosis and decrease in mitosis compared to gemcitabine alone (Right). *This data is from a manuscript under review.*

6) Do you have drug diaries or data on compliance with L-glutamine receipt? If so, please report. If patients were not compliant, the data would be interpreted differently.

We thank the reviewer for highlighting the importance of drug compliance. Oral drug diaries were captured on study and by review, the average compliance to study drug was 98.5% for the collected diaries from all patients during the study period. This reinforces the exquisite tolerability of oral L-glutamine and the highly motivated nature of our study patient population.

7) What is your interpretation or understanding why short survivors had greater alpha diversity? Please explain.

Thank you for highlighting this finding. While higher alpha diversity typically correlates with favorable outcomes in microbiome studies, our data shows short PFS patients had significantly higher basal alpha diversity (Shannon index, p=0.01) versus long PFS patients. This counterintuitive finding could stem from expansion of the truly healthy microbiome (although unlikely in these patients), or due to the opportunistic, pro-inflammatory taxa, inflating Shannon index without conferring benefit. Further studies with higher number of participants are needed to determine if long PFS patients experience microbiome loss over time.

8) No difference in Shannon index (Fig. 5A) but there does seem to be a consistent trend across each analysis towards an increase with L-glutamine (interestingly in the overall and in the long-term survivors, but not the short-term). Please comment whether you believe this is worthy of further analysis and I would recommend if so, you point this out in the text. There is nothing magical about the threshold of p=0.05. While accepted as statistical significant, it's arbitrary, so this may be a type 2 error that could be supported with a larger sample size and would that support your hypothesis?

Thank you for noting this trend. While L-glutamine showed no significant effect on Shannon index (p=0.24, Extended Data Fig. 5A), we agree the consistent directional increase – particularly in overall, long-PFS, and long-OS patients – suggests a potential type II error (false negative) due to limited sample size and low statistical power. This detail has been added to the revised submission.

9) Do the bars in Ex Fig 5C correspond on a per patient level? That is, is the first bar in baseline and and L-glut the same patient and so on? Same question for D. If so, doesn't it show microbiome migration in all cases? Please comment.

The bars in Extended Data Figs. 5C and 5D represent per-patient data, with each patient's baseline and L-glutamine timepoints paired. These plots show patient-specific microbiome shifts pre- and post-L-glutamine, with heterogeneous changes in magnitude of relative abundances of microbial phyla and genera. This is clarified in the figure legend of the revised submission.

10) Please explain Ext Data Fig 7 better. Why are many gene families replicated in the list?

Thank you for this question on Extended Data Fig. 7. Multiple entries for the same gene families, like Glycogen phosphorylase or Enoyl CoA hydratase reflect distinct metabolic reactions that are involved as identified by unique RXN IDs, which reflect their role in pathway-specific reactions. We have revised the results to explicitly note: "Gene families listed by reaction ID and enzyme name; identical enzymes appearing multiple times represent distinct reactions/pathways."

Reviewer #2 (Remarks to the Author):

The authors address an important and timely clinical question, and the overall study is of potential significance. Nevertheless, despite the authors' revisions, several statistical aspects of the analyses presented in Extended Data Figs. 4 and 5 remain insufficiently clarified, particularly with respect to sample definition, subgroup stratification, and analytical transparency.

We thank the reviewer for their comments and suggestions that strengthen the quality of our manuscript. Please find a point-by-point response to the reviewer below.

First, the effective sample sizes contributing to each analysis are still not clearly reported. While Extended Data Fig. 5 indicates n = 12, it is not explicitly stated how these patients relate to the full evaluable cohort (n = 16), nor whether inclusion was determined solely by biospecimen availability. In addition, the numbers of patients contributing to each subgroup comparison (e.g., long vs. short OS or PFS) are not specified. Explicit reporting of subgroup sizes in the figures or legends is necessary for proper interpretation, particularly given the small cohort size.

We thank the reviewer for this suggestion. We have added Extended Data Table 6 in the revised submission to make clear the patient numbers in each group for the long OS/PFS vs. short OS/PFS comparisons in Extended Data Fig. 5a. We refer the reader to the Methods subsection "DNA extraction, shotgun sequencing, and bioinformatics" for clarification that the effective sample sizes were determined by biospecimen availability. We have included sample sizes in the figure legend to clarify the sample sizes for Extended Data Fig. 5b-d.

Second, in Extended Data Fig. 4, plasma metabolites were selected and stratified into "high" and "low" groups based on associations with overall survival, followed by Kaplan–Meier visualization. While Kaplan–Meier methods are appropriate for descriptive estimation of PFS and OS in this phase I study, the outcome-informed nature of metabolite selection and stratification should be more clearly acknowledged, as this approach is inherently exploratory and may introduce optimistic bias in small cohorts. The absence of multiple-testing adjustment further underscores the need to frame these analyses as hypothesis-generating rather than prognostic.

Overall, clearer reporting of sample selection, subgroup sizes, and analytical boundaries—together with consistently cautious framing of outcome-associated findings as exploratory—would substantially strengthen the rigor and interpretability of the results presented in Extended Data Figs. 4 and 5.

We thank the reviewer for raising these valid points. Overall, when relevant, we have indicated the exploratory nature of the metabolomic and microbiome analyses in the abstract and main text to caution the reader while strengthening the rigor and interpretability of our results in Extended Data Figs. 4 and 5. We have included a discussion following the description of Extended Data Fig. 4 results in the main text to emphasize the exploratory and outcome-informed nature of metabolite selection and stratification that underscores the analytic boundaries and renders our findings hypothesis generating. We additionally caution that further evaluation in a larger cohort is needed to inform the biomarker potential of these metabolites. We have clarified subgroup sizes for Extended Data Fig 4 and Extended Data Fig 5 in the revised submission, and we refer the reader to the Methods subsections "Plasma metabolomics" and "DNA extraction, shotgun sequencing, and bioinformatics" for clarification of 1) the number of patients and samples selected for plasma metabolomics and microbiome analyses and 2) that the effective sample sizes were determined by biospecimen availability.

Reviewer #1 (Remarks to the Author):

The authors have addressed all concerns of this reviewer.

We thank the reviewer for approving our revised manuscript.

Reviewer #2 (Remarks to the Author):

I have no further specific comments on the manuscript. One remaining point concerns the presentation of sample sizes in the figures. Given the very limited sample size used in these analyses (only 15 and 12 patients in the respective cohorts), it is important that the exact number of samples in each subgroup be explicitly reported in the relevant figures rather than only providing the total number analyzed. Although the authors note that a larger cohort will be required to further assess the biomarker potential of these metabolites, the current presentation still needs to be more transparent. For example, the Kaplan–Meier curves in Extended Data Fig. 4 should indicate the group sizes directly in the labels (e.g., High (n = xx), Low (n = xx)), and the same applies to the grouped boxplots in Extended Data Fig. 5a. Clear reporting of per-group sample sizes is necessary for readers to evaluate the strength and stability of the observed associations.

We thank the reviewer for taking the time to review our revised manuscript. Although there are no further specific comments on the manuscript, we appreciate the reviewer's recommendation to clearly report the number of samples in each subgroup in the mentioned figures for readers to evaluate the strength and stability of the observed associations. We have accordingly made modifications to include per-group sample sizes for the high- and low-level metabolites (revised Main Fig. 4) and grouped boxplot comparisons (revised Extended Fig. 3), as well as the preceding revised Main Fig. 2 and 3, respectively, where each individual line plot or dot in scatter plot represents n=1 subject for each metabolite. We have updated the Plasma metabolomics section in the Methods to further clarify that although metabolites were collected from n=15 subjects, smaller sample sizes occurred due to biospecimen availability and missing metabolite measurements were excluded from the metabolomic analyses.
