# Supplementary material for: l-Glutamine in combination with first-line gemcitabine and nab-paclitaxel in advanced pancreatic ductal adenocarcinoma: an open-label, single-arm, phase 1 GlutaPanc trial
Source: Nat Cancer. 2026 Aug 31;7(9):1406–18. doi: 10.1038/s43018-026-01225-z (PMC13600916; doi:10.1038/s43018-026-01225-z)
Supplement: Supplementary file 4 [file 43018_2026_1225_MOESM4_ESM.pdf]

Supplementary Tables

Supplementary Table 1. Dosing levels for dose-finding portion of trial

Dose Level[a]	Gemcitabine	Nab-paclitaxel	L-glutamine[b]
-2	600 mg/m^2 IV D1, 8, 15	75 mg/m^2 IV D1, 8, 15	0.1 g/kg oral BID
-1	800 mg/m^2 IV D1, 8, 15	100 mg/m^2 IV D1, 8, 15	0.1 g/kg oral BID
0	1000 mg/m^2 IV D1, 8, 15	125 mg/m^2 IV D1, 8, 15	0.1 g/kg oral BID
1	1000 mg/m^2 IV D1, 8, 15	125 mg/m^2 IV D1, 8, 15	0.2 g/kg oral BID
2	1000 mg/m^2 IV D1, 8, 15	125 mg/m^2 IV D1, 8, 15	0.3 g/kg oral BID

Supplementary Table 2. Treatment-related adverse events

	Event-level (N=169)		Patient-level (N=18)	
**All grade AEs**	169 (100.0%)		17 (94.4%)	
**Any grade ≥3 AEs**	43 (25.4%)		12 (66.7%)	
**Adverse event type**	**All grade**	**Grade ≥3**	**All grade**	**Grade ≥3**
Abdominal pain	1 (0.6%)	0	1 (5.6%)	0
Alanine aminotransferase increased	1 (0.6%)	0	1 (5.6%)	0
Alopecia	3 (1.8%)	0	3 (16.7%)	0
Anemia	22 (13.0%)	11 (6.5%)	8 (44.4%)	5 (27.8%)
Anorexia	5 (3.0%)	1 (0.6%)	5 (27.8%)	1 (5.6%)
Anxiety	1 (0.6%)	0	1 (5.6%)	0
Arthralgia	1 (0.6%)	0	1 (5.6%)	0
Aspartate aminotransferase increased	3 (1.8%)	0	2 (11.1%)	0
Blood bilirubin increased	2 (1.2%)	0	1 (5.6%)	0
Constipation	2 (1.2%)	0	2 (11.1%)	0
Cough	1 (0.6%)	0	1 (5.6%)	0
Dehydration	1 (0.6%)	0	1 (5.6%)	0
Diarrhea	6 (3.6%)	1 (0.6%)	5 (27.8%)	1 (5.6%)
Dizziness	1 (0.6%)	0	1 (5.6%)	0
Dysgeusia	1 (0.6%)	0	1 (5.6%)	0
Dyspnea	1 (0.6%)	0	1 (5.6%)	0
Edema limbs	9 (5.3%)	0	6 (33.3%)	0
Fall	1 (0.6%)	0	1 (5.6%)	0
Fatigue	15 (8.9%)	2 (1.2%)	10 (55.6%)	2 (11.1%)
Febrile neutropenia	2 (1.2%)	2 (1.2%)	2 (11.1%)	2 (11.1%)
Flatulence	1 (0.6%)	0	1 (5.6%)	0
Heart failure	1 (0.6%)	0	1 (5.6%)	0
Hypokalemia	3 (1.8%)	0	3 (16.7%)	0
Hyponatremia	1 (0.6%)	0	1 (5.6%)	0
Muscle weakness lower limb	1 (0.6%)	0	1 (5.6%)	0
Nausea	3 (1.8%)	0	3 (16.7%)	0
Neutrophil count decreased	19 (11.2%)	10 (5.9%)	9 (50.0%)	7 (38.9%)
Peripheral sensory neuropathy	9 (5.3%)	1 (0.6%)	5 (27.8%)	1 (5.6%)
Platelet count decreased	19 (11.2%)	7 (4.1%)	7 (38.9%)	4 (22.2%)
Pneumonitis	1 (0.6%)	1 (0.6%)	1 (5.6%)	1 (5.6%)
Pruritus	1 (0.6%)	0	1 (5.6%)	0
Rash maculo-papular	7 (4.1%)	0	4 (22.2%)	0
Scalp pain	1 (0.6%)	0	1 (5.6%)	0
Superficial thrombophlebitis	1 (0.6%)	0	1 (5.6%)	0
Syncope	1 (0.6%)	1 (0.6%)	1 (5.6%)	1 (5.6%)
Thrush	2 (1.2%)	0	2 (11.1%)	0
Vomiting	3 (1.8%)	0	3 (16.7%)	0
Weight loss	1 (0.6%)	0	1 (5.6%)	0
White blood cell decreased	15 (8.9%)	6 (3.6%)	8 (44.4%)	5 (27.8%)

AE, adverse event

Supplementary Table 3. All adverse events treatment-related or treatment-unrelated

	Event-level (N=245)		Patient-level (N=18)	
**Any AEs**	245 (100.0%)		17 (94.4%)	
**Any grade ≥3 AEs**	61 (24.9%)		15 (83.3%)	
**Adverse event type**	**All grade**	**Grade ≥3**	**All grade**	**Grade ≥3**
Abdominal pain	2 (0.8%)	0	2 (11.1%)	0
Alanine aminotransferase increased	1 (0.4%)	0	1 (5.6%)	0
Alkaline phosphatase increased	4 (1.6%)	1 (0.4%)	2 (11.1%)	1 (5.6%)
Alopecia	3 (1.2%)	0	3 (16.7%)	0
Anemia	23 (9.4%)	12 (4.9%)	9 (50.0%)	6 (33.3%)
Anorexia	5 (2.0%)	1 (0.4%)	5 (27.8%)	1 (5.6%)
Anxiety	2 (0.8%)	0	2 (11.1%)	0
Arthralgia	1 (0.4%)	0	1 (5.6%)	0
Ascites	2 (0.8%)	0	1 (5.6%)	0
Aspartate aminotransferase increased	3 (1.2%)	0	2 (11.1%)	0
Back pain	1 (0.4%)	0	1 (5.6%)	0
Bloating	1 (0.4%)	0	1 (5.6%)	0
Blood bilirubin increased	5 (2.0%)	0	2 (11.1%)	0
Cardiac troponin I increased	1 (0.4%)	0	1 (5.6%)	0
Chest pain - cardiac	1 (0.4%)	0	1 (5.6%)	0
Colitis	1 (0.4%)	0	1 (5.6%)	0
Constipation	3 (1.2%)	0	3 (16.7%)	0
Cough	2 (0.8%)	0	2 (11.1%)	0
Creatinine increased	1 (0.4%)	0	1 (5.6%)	0
Dehydration	2 (0.8%)	0	2 (11.1%)	0
Delirium	1 (0.4%)	0	1 (5.6%)	0
Diarrhea	7 (2.9%)	1 (0.4%)	6 (33.3%)	1 (5.6%)
Disseminated intravascular coagulation	1 (0.4%)	0	1 (5.6%)	0
Dizziness	1 (0.4%)	0	1 (5.6%)	0
Dysgeusia	1 (0.4%)	0	1 (5.6%)	0
Dysphagia	1 (0.4%)	1 (0.4%)	1 (5.6%)	1 (5.6%)
Dyspnea	4 (1.6%)	0	4 (22.2%)	0
Edema limbs	11 (4.5%)	0	7 (38.9%)	0
Epistaxis	1 (0.4%)	0	1 (5.6%)	0
Fall	1 (0.4%)	0	1 (5.6%)	0
Fatigue	16 (6.5%)	2 (0.8%)	11 (61.1%)	2 (11.1%)

Febrile neutropenia	2 (0.8%)	2 (0.8%)	2 (11.1%)	2 (11.1%)
Fever	1 (0.4%)	0	1 (5.6%)	0
Flatulence	1 (0.4%)	0	1 (5.6%)	0
Heart failure	1 (0.4%)	0	1 (5.6%)	0
Hypertension	5 (2.0%)	3 (1.2%)	3 (16.7%)	3 (16.7%)
Hypocalcemia	1 (0.4%)	0	1 (5.6%)	0
Hypoglycemia	1 (0.4%)	0	1 (5.6%)	0
Hypokalemia	5 (2.0%)	1 (0.4%)	4 (22.2%)	1 (5.6%)
Hyponatremia	2 (0.8%)	0	2 (11.1%)	0
Infections and infestations - Other, specify	1 (0.4%)	0	1 (5.6%)	0
Insomnia	3 (1.2%)	0	3 (16.7%)	0
Ischemia cerebrovascular	1 (0.4%)	0	1 (5.6%)	0
Leukocytosis	1 (0.4%)	1 (0.4%)	1 (5.6%)	1 (5.6%)
Lung infection	1 (0.4%)	0	1 (5.6%)	0
Muscle weakness lower limb	1 (0.4%)	0	1 (5.6%)	0
Myocardial infarction	1 (0.4%)	1 (0.4%)	1 (5.6%)	1 (5.6%)
Nasal congestion	1 (0.4%)	0	1 (5.6%)	0
Nausea	5 (2.0%)	1 (0.4%)	5 (27.8%)	1 (5.6%)
Neck pain	1 (0.4%)	0	1 (5.6%)	0
Neutrophil count decreased	19 (7.8%)	10 (4.1%)	9 (50.0%)	7 (38.9%)
Obstruction gastric	1 (0.4%)	1 (0.4%)	1 (5.6%)	1 (5.6%)
Pain	1 (0.4%)	0	1 (5.6%)	0
Peripheral sensory neuropathy	10 (4.1%)	1 (0.4%)	6 (33.3%)	1 (5.6%)
Platelet count decreased	19 (7.8%)	7 (2.9%)	7 (38.9%)	4 (22.2%)
Pleural effusion	1 (0.4%)	0	1 (5.6%)	0
Pneumonitis	1 (0.4%)	1 (0.4%)	1 (5.6%)	1 (5.6%)
Presyncope	1 (0.4%)	1 (0.4%)	1 (5.6%)	1 (5.6%)
Pruritus	1 (0.4%)	0	1 (5.6%)	0
Rash maculo-papular	7 (2.9%)	0	4 (22.2%)	0
Rhinorrhea	1 (0.4%)	0	1 (5.6%)	0
Scalp pain	1 (0.4%)	0	1 (5.6%)	0
Sepsis	3 (1.2%)	3	2 (11.1%)	2

		(1.2%)		(11.1%)
Skin and subcutaneous tissue disorders - Other, specify	1 (0.4%)	0	1 (5.6%)	0
Skin infection	1 (0.4%)	1 (0.4%)	1 (5.6%)	1 (5.6%)
Skin ulceration	1 (0.4%)	0	1 (5.6%)	0
Stroke	1 (0.4%)	0	1 (5.6%)	0
Superficial thrombophlebitis	1 (0.4%)	0	1 (5.6%)	0
Syncope	2 (0.8%)	2 (0.8%)	2 (11.1%)	2 (11.1%)
Thromboembolic event	2 (0.8%)	1 (0.4%)	2 (11.1%)	1 (5.6%)
Thrush	2 (0.8%)	0	2 (11.1%)	0
Upper respiratory infection	1 (0.4%)	0	1 (5.6%)	0
Urinary tract infection	1 (0.4%)	0	1 (5.6%)	0
Vaginal infection	1 (0.4%)	0	1 (5.6%)	0
Vomiting	5 (2.0%)	0	4 (22.2%)	0
Weight loss	1 (0.4%)	0	1 (5.6%)	0
White blood cell decreased	15 (6.1%)	6 (2.4%)	8 (44.4%)	5 (27.8%)

AE, adverse event

Supplementary Table 4. Post-progression therapies and progression-free survival.

**Characteristic (n=14)[a]**	**n (%)**
Lines of therapy	
1[b]	2 (14%)
2	5 (36%)
3	2 (14%)
4	5 (36%)
Post-progression regimens	
<u>Second-line (n=12):</u>	
FOLFIRINOX	5 (42%)
5-FU/liposomal irinotecan	4 (33%)
Gemcitabine/capecitabine	2 (17%)
NALIRIFOX	1 (8%)
<u>Third-line (n=7):</u>	
Gemcitabine/nab-paclitaxel	1 (14%)
FOLFIRINOX	1 (14%)
IROX	1 (14%)
Clinical trial	4 (58%)
<u>Fourth-line (n=5):</u>	
Cisplatin/gemcitabine	2 (40%)
Cisplatin/gemcitabine/nab-	1 (20%)
paclitaxel	1 (20%)
FOLFOX	1 (20%)
Post-progression PFS (median, range)	
Second-line PFS	2 months (0.7-13.2 months)
Third-line PFS	1.6 months (0.5-4.3 months)
Fourth-line PFS	1.2 months (0.7-3.0 months)

[a]Two subjects did not have disease progression at time of data cut off

[b]Two subjects did not receive subsequent systemic therapies upon progression to study treatment

FOLFIRINOX, 5-fluorouracil or 5-FU, leucovorin, irinotecan, oxaliplatin; NALIRIFOX, liposomal irinotecan, 5-FU, leucovorin, oxaliplatin; IROX, irinotecan and oxaliplatin; FOLFOX, 5-FU, leucovorin, oxaliplatin; PFS, progression-free survival

Supplementary Table 5. Plasma metabolites that exhibited significant changes either between baseline and C1D1 or between C1D1 and C1D8 are bolded.

		**Baseline**			**Wilcoxon matched-pairs signed rank test – two-sided P Value**	
		Baseline	C1D1	C1D8	Baseline vs C1D1	C1D1 vs C1D8
1	**2-Deoxy-D-glucose 6-phosphate**	158	365	465	**0.0276**	0.9097
2	**2-Ketobutyrate**	486	290	478	**0.0479**	0.1294
3	2-Methyl-1-butanol	2609	4488	1676	0.1465	**0.0161**
4	**3-2-Hydroxyethylindole**	34	22	22	**0.0386**	0.5771
5	**3-Methylglutaric acid**	15032	24336	10660	**0.0215**	**0.0005**
6	alpha-Ketoglutaric acid	28679	39224	20901	0.0681	**0.0049**
7	**Cellobiose**	1068	1705	1202	**0.0479**	0.1348
8	cis-Aconitic acid	771321	780217	680450	0.7354	**0.0425**
9	Cytidine-5-monophosphate	33	41	25	0.2439	**0.0269**
10	**D-Mannose**	360349	470598	502376	**0.0002**	0.3013
11	Glyoxylic acid	140	256	43	0.3396	**0.0186**
12	Inosine 5-monophosphate	531	636	127	0.5879	**0.02**
13	L-Arabitol	1554	3578	1450	0.3757	**0.0425**
15	L-Hydroxyglutaric acid	3116	4451	3180	0.1099	**0.021**
16	L-Malic acid	236776	273681	230564	0.0574	**0.0068**
17	**L-Sorbose**	360349	470598	502376	**0.0002**	0.3013
18	**Lactic acid**	462716	551143	484296	**0.0479**	0.2036
19	**Mevalonic acid**	264	1572	233	**0.0398**	**0.0449**
20	**Myo-Inositol**	9287040	10788618	11126479	**0.0007**	0.6221
21	**N-Carbamoyl-DL-aspartic acid**	786	1191	1455	**0.0266**	0.5693
22	**Nicotinic acid mononucleotide**	182	104	107	**0.0425**	0.7334
23	Phenylpyruvic acid	1282	2074	1194	0.1099	**0.021**
24	Pyridoxal hydrochloride	1060	1501	426	0.3757	**0.0034**
25	**Pyruvic acid**	1435	3898	1869	**0.0398**	0.2334
26	Quinic acid	5971	8570	3300	0.1677	**0.0122**
27	**Taurocholic acid**	5952	890	2988	**0.0046**	**0.0161**
28	trans-Aconitic acid	607771	631649	573170	0.3054	**0.0122**
29	**Trehalose**	155	262	250	**0.0171**	0.8501
30	Uridine 5-diphosphate	129	131	92	0.7354	**0.0171**

Supplementary Table 6. The impact of L-glutamine on overall survival (OS) and progression free survival (PFS) based on alpha diversity of the gut microbiome in patients treated with L-glutamine and gemcitabine plus nab-paclitaxel (in reference to Extended Data Figure 5a comparisons). Long OS was defined by patients whose median OS surpassed the historical median OS (258 days), while long PFS was defined by those patients having surpassed historical median PFS (165 days) of first-line gemcitabine and nab-paclitaxel alone.

	N	Baseline N = 12 (%)	L-Glutamine N = 11 (%)
**OS**	23		
**Long OS**		10 (83%)	9 (82%)
**Short OS**		2 (17%)	2 (18%)
**PFS**	23		
**Long PFS**		9 (75%)	8 (73%)
**Short PFS**		3 (25%)	3 (27%)

Supplementary Table 7. Baseline characteristics and evidence of weight stabilizing effects from L-glutamine and first-line gemcitabine and nab-paclitaxel (n = 16).

Characteristic	Frequency (%) or mean ± SD
Median age in years (range)	71 (55-92)
Female	11/16 (69%)
Cachexia at study entry[a]	13/16 (81%)
Mean weight (kg) at study entry	73.1 (21.3)
Mean BMI (kg/m^2) at study entry	26.2 (6.4)
Weight stability[b]	8/16 (50%)
Median duration of treatment	8.6 months (95% CI 6-15)
Weight increase from baseline	8/16 (50%)
BMI increase from baseline	8/16 (50%)
Mean weight change (kg) from baseline	-0.39 (6.7)
Mean BMI change (kg/m^2) from baseline	-0.17 (2.7)

[a]Defined as >5% unexplained weight loss within 6 months prior to screening
[b]Defined as weight change less than 0.1 kg/baseline BMI-unit from cycle 1, day 1 of study treatment to end of treatment (median duration of treatment 8.6 months)
SD, standard deviation; CI, confidence interval

Supplementary Table 8. Association between weight stability and overall survival (OS) and progression free survival (PFS; n=16).

	**p-value from Fisher's exact two-sided test**	**p-value from exact log-rank two-sided test**	
	**Best overall response** CR+PR vs. SD+PD	**OS** dead vs. alive	**PFS** PD vs. PF
**Weight stability**[a] Yes vs. No	>0.999	0.682	0.742

[a]Defined as weight change less than 0.1 kg/baseline BMI-unit from cycle 1, day 1 of study treatment to end of treatment (median duration of treatment 8.6 months)
CR, complete response; PR, partial response; SD, stable disease; PD, progressive disease; OS, overall survival; PFS, progression-free survival; PF, progression free

Supplementary Table 9. Association between weight stability and efficacy end points (n=16).

	**Mean ± standard deviation**		**p-value (Mann-Whitney U two-sided test)**	**p-value (Cox regression, two-sided test)**
**Tumor shrinkage**[a], %	Weight stability[b] **Yes** n=8	Weight stability **No** n=8	0.958	.
	-36.09 ± 45.47	-32.04 ± 31.13		
**Weight change**, kg	Best overall response **CR+PR** n=7	Best overall response **SD+PD** n=9	0.289	.
	-2.39 ± 7.11	1.17± 6.77		
	OS **dead** n=10	OS **alive** n=6	0.871	0.790
	-0.97 ± 7.74	0.58 ± 5.86		
	PFS **PD** n=14	PFS **PF** n=2	0.382	0.515
	-1.06 ± 6.88	4.30 ± 7.35		
**BMI change**	Best overall response **CR+PR** n=7	Best overall response **SD+PD** n=9	0.091	.
	-1.30 ± 2.57	0.72± 2.86		
	OS **dead** n=10	OS **alive** n=6	0.492	0.966
	-0.52 ± 3.22	0.42 ± 2.20		
	PFS **PD** n=14	PFS **PF** n=2	0.600	0.470
	-0.36 ± 2.89	1.21 ± 2.80		

[a]Tumor shrinkage defined as any reduction in tumor target lesions from baseline
[b]Defined as weight change less than 0.1 kg/baseline BMI-unit from cycle 1, day 1 of study treatment to end of treatment (median duration of treatment 8.6 months)
CR, complete response; PR, partial response; SD, stable disease; PD, progressive disease; OS, overall survival; OS, overall survival; PF, progression free; BMI, body mass index; PFS, progression-free survival
